# Positive feedback loop involving AMPK and CLYBL acetylation links metabolic rewiring and inflammatory responses

**DOI:** 10.1038/s41419-025-07362-0

**Published:** 2025-01-25

**Authors:** Wenke Wang, Boquan Wu, Mingjun Hao, Sichong Chen, Ruiting Cong, Wenjie Wu, Pengbo Wang, Qiaoyi Zhang, Pengyu Jia, Yuequn Song, Bo Liu, Siyao Qu, Jian-Fei Pei, Da Li, Naijin Zhang

**Affiliations:** 1 Center of Reproductive Medicine, Shengjing Hospital of China Medical University, Shenyang, 110004 China; 2 NHC Key Laboratory of Advanced Reproductive Medicine and Fertility (China Medical University), National Health Commission, Shenyang, 110004 China; 3 Department of Cardiology, The First Hospital of China Medical University, Shenyang, Liaoning 110001 China; 4 Department of Pharmaceutical Toxicology, School of Pharmacy, China Medical University, Shenyang, 110122 China; 5 China Medical University School of Public Health, Shenyang, 110122 China; 6 Department of Neurosurgery, the Fourth Affiliated Hospital of China Medical University, Shenyang, Liaoning 110032 China; 7 Department of Cardiac Surgery, The First Hospital of China Medical University, Shenyang, Liaoning 110001 China; 8 Department of Medical Genetics, China Medical University, Shenyang, Liaoning 110122 China; 9 Institute of Health Sciences, China Medical University, Shenyang, Liaoning 110122 China

**Keywords:** Heart failure, Acetylation

## Abstract

Metabolic rewiring underlies effective macrophages defense to respond disease microenvironment. However, the underlying mechanisms driving metabolic rewiring to enhance macrophage effector functions remain unclear. Here, we demonstrated that the metabolic reprogramming in inflammatory macrophages depended on the acetylation of CLYBL, a citramalyl-CoA lyase, at lysine 154 (K154), and blocking CLYBL-K154 acetylation restricted the release of pro-inflammatory factors. Mechanistically, we found a crucial AMPK-CLYBL acetylation positive feedback loop, triggered by toll-like receptors (TLRs), involving AMPK hypophosphorylation and CLYBL hyperacetylation. The deacetylase enzyme SIRT2 acted as the bridge between AMPK phosphorylation and CLYBL acetylation, thereby regulating macrophage polarization and the release of pro-inflammatory cytokines. Furthermore, CLYBL hypoacetylation decreased monocyte infiltration, thereby alleviating cardiac remodeling. These findings suggest that the AMPK-CLYBL acetylation positive feedback loop serves as a metabolic switch driving inflammatory response and inhibiting CLYBL-K154 acetylation may offer a promising therapeutic strategy for inflammatory response-related disorders.

## Introduction

Inflammation is a dynamic process orchestrated by immune cells that prevent the spread of pathogens and promote tissue repair [1]. While moderate immune response activation is usually a protective response triggered by a stimulus, dysregulated immune signaling can cause excessive cytokine production, resulting in tissue damage [2]. In response to pathogen- or damage-associated molecular patterns (PAMPs or DAMPs, respectively), bone marrow-derived monocytes are recruited to mammalian tissues and differentiate into macrophages to remove pathogens and damaged cells [3, 4]. Macrophages are highly plastic, and act as pro-inflammatory, homeostatic, and anti-inflammatory agents when polarize [5]. Pro-inflammatory macrophages drive inflammatory responses and activate adaptive immunity, whereas excessive pro-inflammatory cytokines released by macrophages can also drive disease progression through cell-to-cell cooperation [6]. Therefore, controlling the release of excessive pro-inflammatory cytokines could be an effective therapy for inflammatory response-related disorders.

Recent studies have shown that metabolic reprogramming underlies the function of macrophages [7]. The polarization of macrophages to the pro-inflammatory phenotype is driven by TLRs and this process accompanies with remodeling of the tricarboxylic acid (TCA) cycle [8, 9]. Metabolites of the TCA cycle, such as succinate, fumarate, and the by-product itaconate, are crucial for controlling the immunophenotype of macrophages [7, 10–12]. With the advancements in research on itaconate and immunometabolites, the ubiquitously expressed mitochondrial enzyme CLYBL has garnered attention. CLYBL is a citramalyl-CoA lyase that completes the C5-dicarboxylate metabolic pathway. This pathway metabolizes TCA cycle by-products, such as itaconate, to acetyl-CoA and pyruvate [13]. Therefore, CLYBL may lead to itaconate depletion and subsequent inflammation. Although CLYBL carries double allele, the homozygous loss of CLYBL is tolerated in healthy individuals [14]. A clinical study reported that CLYBL levels are increased in the macrophages of patients with COVID-19 [15]. These studies revealed that CLYBL may be an important target in therapeutic strategies involving the immunometabolic control of macrophages, without side effects.

Previous studies have focused on the metabolites involved in metabolic reprogramming and the mechanisms by which they regulate cytokine production [7]. However, the mechanisms that trigger metabolic alterations in macrophages upon stimulation remain unclear. TLR activation in macrophages directs pro-inflammatory cytokine production through a transcriptional cascade response [16], but changes in TLR-mediated metabolic pathways require the regulation of metabolism-related signaling. AMP-activated protein kinase (AMPK) is a critical regulator of the TCA cycle and regulates cellular energy homeostasis by sensing the metabolic status of the cell [17]. The classical pathway by which AMPK regulates metabolism is through the direct phosphorylation of key proteins, but AMPK also regulates the acetylation of proteins by increasing the nicotinamide adenine dinucleotide (NAD^+^) content [18–21]. AMPK acts as a pivotal negative immunometabolic regulator of macrophage activation. AMPKα1 knockout leads to not only a striking M1 hyperpolarization but also the increased expression of rate-limiting enzymes from multiple metabolic pathways in macrophages [22]. In addition, the AMPK activator, metformin, decreases the secretion of pro-inflammatory cytokines by macrophages and is known to suppress cardiovascular inflammation in humans [23]. Therefore, AMPK may be a key metabolic switch in TLR-mediated metabolic reprogramming. However, the regulatory paradigms underlying TLR-AMPK signaling-mediated immunometabolism require further investigation.

Here, we uncovered an AMPK-CLYBL acetylation positive feedback loop that serves as a metabolic switch to induce an inflammatory response. This mechanism is a pivotal determinant of the metabolic rewiring of inflammatory macrophages. Quantitative acetylome profiling revealed that CLYBL is acetylated at lysine 154, CBP is the acetyltransferase and SIRT2 is the deacetylase of CLYBL. The acetylation mimetic CLYBL-K154R mutant attenuates TLR-induced inflammatory reactions in macrophages, and CLYBL acetylation is regulated by the AMPK signaling pathway. After exposure to stimuli, macrophages infiltrate the heart. Decreased AMPK phosphorylation in activated macrophages induces increased CLYBL acetylation, and the positive feedback loop involving the hypophosphorylation of AMPK and hyperacetylation of CLYBL exacerbates the release of pro-inflammatory cytokines. The sustained activation of the AMPK-CLYBL acetylation axis aggravates cardiac remodeling through cell-to-cell interactions. These findings demonstrate the crucial role of the TLR-AMPK-CLYBL acetylation axis in inflammatory reactions and highlight CLYBL-K154 as a potential therapeutic target for inflammatory response-related disorders.

## Results

### Quantitative acetylome profiling identifies CLYBL as a physiological substrate of SIRT2

**Fig. 1:**
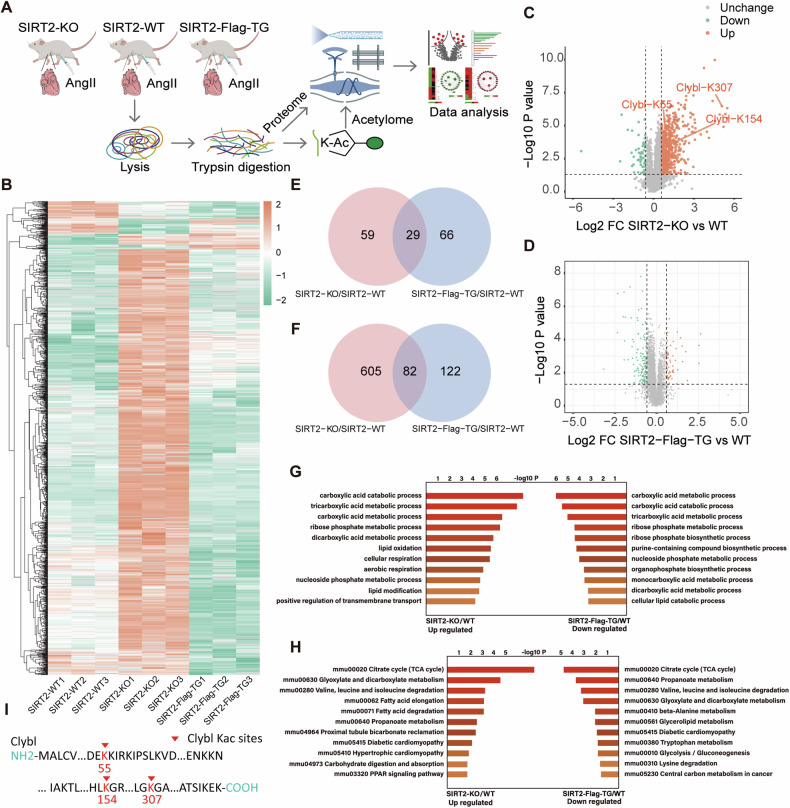
Quantitative acetylome profiling identifies CLYBL as a physiological substrate of SIRT2. **A** Proteomics and modification analyses among cardiac tissues of SIRT2-KO, SIRT2-WT, and SIRT2-Flag-TG mice treated with Ang II. **B** Heatmap of different acetylated proteins among SIRT2-KO, SIRT2-WT, and SIRT2-Flag-TG mouse heart samples. **C** Volcano plot showing differential acetylation sites in SIRT2-KO versus SIRT2-WT (*p* < 0.05, fold change>1.5). **D** Volcano plot showing differential acetylation sites in SIRT2-Flag-TG versus SIRT2-WT (*p* < 0.05, fold change>1.5). **E** Venn diagram showing the shared and specific acetylation-modified proteins among SIRT2-KO versus SIRT2-WT and SIRT2-Flag-TG versus SIRT2-WT. **F** Venn diagram showing the shared and specific acetylated sites among SIRT2-KO versus SIRT2-WT and SIRT2-Flag-TG versus SIRT2-WT. **G** Histogram showing enrichment of biological process pathways of differential proteins, whose acetylation levels were downregulated in SIRT2-Flag-TG and upregulated in SIRT2-KO. **H** Histogram showing the KEGG pathways enrichment of distinct proteins, whose acetylation levels were downregulated in SIRT2-Flag-TG and upregulated in SIRT2-KO. **I** Lysine residues, highlighted in red text, demonstrating possible sites modified by SIRT2 in amino acid sequences of CLYBL.

To identify the roles of protein acetylation in mouse heart tissue, a comprehensive quantitative acetylome profiling study was conducted among wild-type (WT), SIRT2 knockout (SIRT2-KO), and SIRT2-Flag-TG (transgenic) mice treated with angiotensin II (Ang II) (Fig. 1A and Fig. S1A–S1G). Acetylome profiling identified and quantified 6,101 acetylation sites and 1,888 acetylated proteins in the cardiac tissues of WT, SIRT2-KO, and SIRT2-Flag-TG mice (Fig. 1B). The differentially expressed and acetylated proteins in the SIRT2-KO and SIRT2-Flag-TG groups compared to those in the corresponding WT tissues are shown in volcano plots with statistical significance (*p* < 0.05, fold change>1.5). Compared to that in the WT tissues, significant changes in the acetylation sites were observed in CLYBL-K154 (*p* < 0.001), K307 (*p* < 0.001) and K55 (*p* < 0.001) in SIRT2-KO group. Therefore, SIRT2 may strongly regulate CLYBL acetylation (Fig. 1C, D). In the comparative analysis of SIRT2-KO, SIRT2-Flag-TG, and their corresponding WT tissues, 29 proteins overlapped in differentially acetylated proteins. Within this set, 59 proteins were exclusively observed in the KO/WT comparison, whereas 66 proteins exhibited specificity in the TG/WT comparison. Remarkably, 82 acetylation sites were concurrently present in both groups, with 605 sites unique to the KO/WT comparison and 122 sites unique to the TG/WT comparison (Fig. 1E, F). Biological processes such as the carboxylic acid metabolic process, TCA metabolic process, and carboxylic acid catabolic process were significantly enriched in SIRT2-KO and downregulated in SIRT2-Flag-TG compared to those in the corresponding WT (Fig. 1G). The KEGG result showed that pathways such as the mmu00020 TCA cycle, mmu00630 glyoxylate and dicarboxylate metabolism were significantly enriched in SIRT2-KO and downregulated in SIRT2-Flag-TG compared with those in the corresponding WT (Fig. 1H). Quantitative acetylome profiling identified CLYBL as a SIRT2 substrate with three acetylation sites (Fig. 1I). Direct mutations at each site were introduced to investigate the regulation of CLYBL by SIRT2.

### SIRT2/CBP deacetylates/acetylates CLYBL at K154

**Fig. 2:**
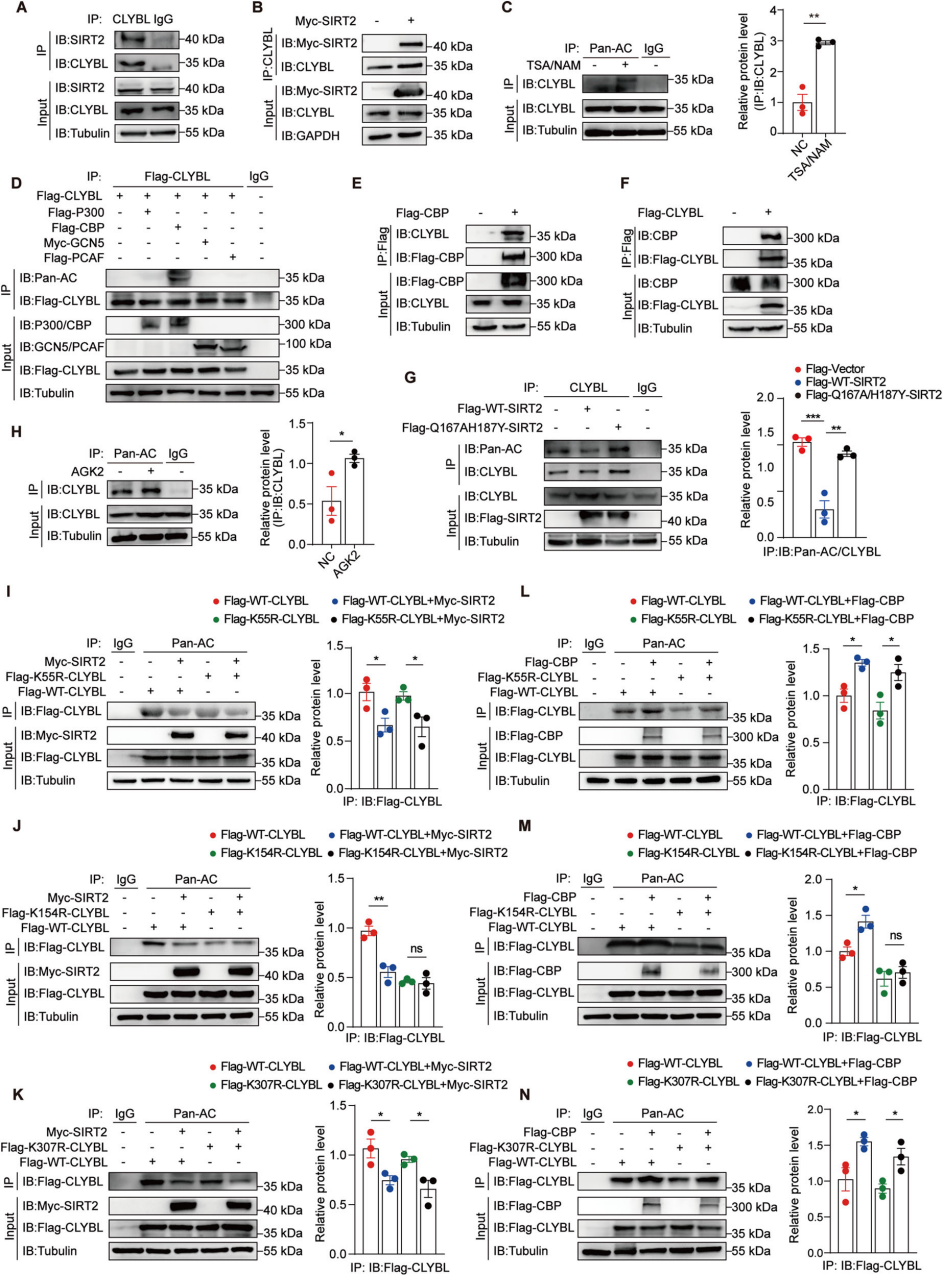
SIRT2/CBP deacetylates/acetylates CLYBL at K154. **A**, **B** Co-immunoprecipitation assay showing the interaction of CLYBL with SIRT2 in 293 T cells. **C** 293 T cells treated with or without trichostatin A (TSA; 0.5 μM, 16 h) and nicotinamide (NAM; 5 mM, 4 h) to detect the acetylation of CLYBL (**p < 0.01). **D** Immunoprecipitation assay determining the acetyltransferase of CLYBL in 293 T cells. **E**, **F** Co-immunoprecipitation assay showing the interaction of CBP with CLYBL in 293 T cells. **G** 293 T cells transfected with Flag-WT-SIRT2, Flag-Q167AH187Y-SIRT2 or Flag-Vector to confirm the suppression of CLYBL acetylation by SIRT2 (**p < 0.01; ***p < 0.001). **H** In 293 T cells, acetylation of CLYBL was detected following treatment with or without AGK2 (20 μM, 24 h) (*p < 0.05). 293 T cells transfected with Flag-WT-CLYBL or Flag-K55R-CLYBL (Mut) (**I**), Flag-WT-CLYBL or Flag-K154R-CLYBL (Mut) (**J**), Flag-WT-CLYBL or Flag-K307R-CLYBL (Mut) (**K**), and with the presence or absence of Myc-SIRT2 to determine the degree of CLYBL acetylation (*p < 0.05; **p < 0.01; ns, not significant). 293 T cells were transfected with Flag-WT-CLYBL or Flag-K55R-CLYBL (Mut) (**L**), Flag-WT-CLYBL or Flag-K154R-CLYBL (Mut) (**M**), Flag-WT-CLYBL or Flag-K307R-CLYBL (Mut) (**N**), and in the presence or absence of Flag-CBP to determine the degree of CLYBL acetylation (*p < 0.05; ns, not significant).

To confirm the regulatory mechanisms underlying CLYBL acetylation, we assessed both endogenous and exogenous interactions between SIRT2 and CLYBL using co-immunoprecipitation assays (Fig. 2A, B). Given that SIRT2 interacts with CLYBL, we confirmed that CLYBL was acetylated and that the acetylation status of CLYBL was regulated by trichostatin A (TSA), an inhibitor of histone deacetylases I and II, and nicotinamide (NAM), an inhibitor of the sirtuin family (Fig. 2C). To identify the acetyltransferase of CLYBL, we transfected cells with P300, CBP, PCAF, and GCN5. Figure 2D showed that only CBP enhanced CLYBL acetylation, whereas the other acetyltransferases had little effect. Interactions between CBP and CLYBL were also observed (Figs. 2E, F). Thus, CLYBL is a target of the deacetylase SIRT2 and the acetyltransferase CBP.

Next, we determined whether CLYBL acetylation is affected by SIRT2. Indeed, SIRT2-WT significantly reduced CLYBL acetylation, but unvaried in SIRT2 inactivation mutation (SIRT2-Q167AH187Y) (Fig. 2G). Moreover, CLYBL acetylation levels were higher in the SIRT2 inhibitor (AGK2)-treated cells than without (Fig. 2H). Thus, SIRT2 regulates CLYBL via deacetylation, further supporting the notion that CLYBL is a SIRT2 substrate.

Given that the acetylation levels of the K55, K154, and K307 sites on CLYBL were increased in the SIRT2-KO mice, we assessed the changes in the degree of acetylation of these sites on CLYBL under physiological conditions using co-immunoprecipitation assays. We mutated the lysine (K) 55, 154, and 307 residues to arginine (R; a non-acetylatable mutant). SIRT2 overexpression markedly reduced the acetylation levels of CLYBL-WT and was also reduced in both CLYBL-K55R and CLYBL-K307R. However, only CLYBL-K154R was maintained at a very low level, regardless of SIRT2 overexpression (Fig. 2I, J, K). Similarly, the degree of acetylation was nearly unvaried in CLYBL-K154R with CBP overexpression but elevated in both CLYBL-K55R and CLYBL-K307R observed with CBP overexpression (Fig. 2L, M, N). These results indicate that CLYBL-K154 is the key site of CLYBL acetylation and is modulated by the deacetylase SIRT2 and acetyltransferase CBP.

### CLYBL acetylation is negatively regulated by AMPK in macrophages

TLRs are crucial in regulating the catabolic program of mitochondrial oxidative phosphorylation (OXPHOS) for energy generation in immunocytes, and this process is partially mediated by the conserved energy sensor AMPK [24]. AMPK activation increases glucose uptake, lipid oxidation, and mitochondrial biogenesis, which are dampened by activated TLRs [25]. In addition, AMPK inhibits immunocyte activation [22]. Given that CLYBL is a citramalyl-CoA lyase involved in the TCA cycle, we speculated that the acetylation level of CLYBL in macrophages may be correlated with AMPK activity.

**Fig. 3:**
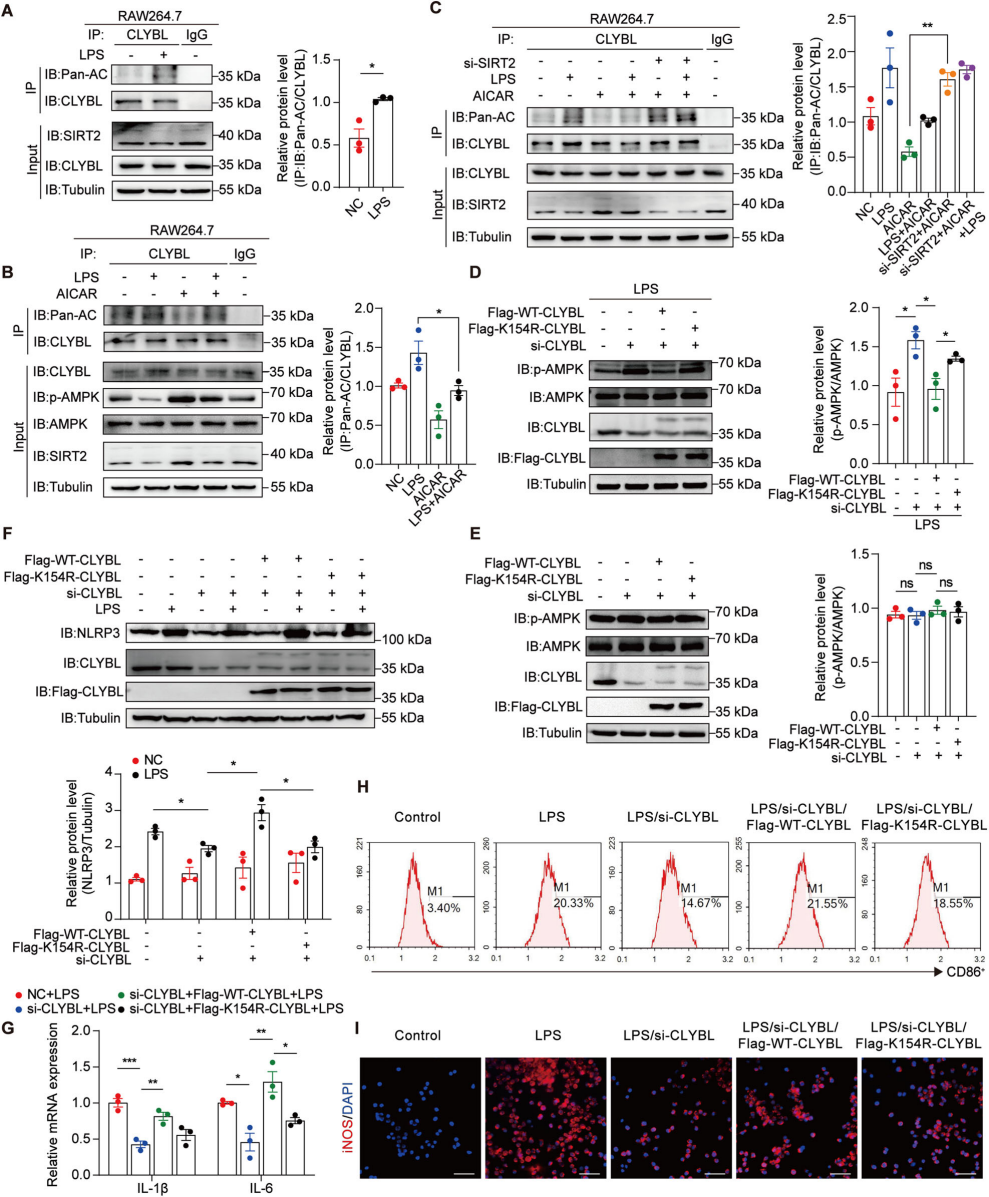
CLYBL acetylation is negatively regulated by AMPK and the inactivation of CLYBL-K154 hinders the activation of pro-inflammatory macrophages. **A** Co-immunoprecipitation detected the acetylation of CLYBL with or without LPS (1 μg/mL, 8 h) in RAW264.7 cells (*p < 0.05). **B** After stimulation with PBS, LPS, AICAR, or a combination of LPS and AICAR (1 μg/mL, 8 h; 500 μM, 6 h), co-immunoprecipitation was used to detect the acetylation of CLYBL in RAW264.7 cells (*p < 0.05). **C** RAW264.7 cells transfected NC or siSIRT2 with or without PBS, LPS, AICAR, or a combination of LPS and AICAR (1 μg/mL, 8 h; 500 μM, 6 h), immunoprecipitation was used to detect the acetylation of CLYBL (**p < 0.01). **D** In vitro, western blotting showed that the CLYBL K154R mutant had higher expression of p-AMPK than Flag-WT-CLYBL in the presence of LPS (*p < 0.05). **E** Western blot showed no significant differences in p-AMPK expression between CLYBL K154R mutant and Flag-WT-CLYBL under basal conditions (ns, not significant). **F** Western blot showed reduced NLRP3 expression in CLYBL K154R mutant compared to Flag-WT-CLYBL in the presence of LPS (*p < 0.05). **G** Quantitative real-time PCR (qPCR) was used to measure the levels of pro-inflammatory factors IL-1β and IL-6 (*p < 0.05; **p < 0.01; ***p < 0.001). **H** Percentages of CD86-positive fractions were assessed using flow cytometry to determine the anti-inflammatory effect of CLYBL-K154R. **I** The expression of iNOS was examined using immunofluorescence staining (iNOS red, DAPI blue; Scale bar, 50 μm).

To test this hypothesis, we used lipopolysaccharide (LPS) to mimic the activation of TLR and found that the phosphorylation of AMPK at Thr-172 diminished after exposure to LPS (Fig. S2A), but the degree of CLYBL acetylation was markedly increased (Fig. 3A). To further assess whether AMPK activity is associated with CLYBL acetylation, the acetylation level of CLYBL was detected in LPS-stimulated cells with or without AICAR, a commonly used AMPK-specific agonist. AMPK activation inhibited the LPS-mediated increase in CLYBL acetylation (Fig. 3B, S2B). Moreover, AICAR-treated macrophages rescued the LPS-stimulated depletion of SIRT2, indicating that the acetylation level of CLYBL may simply be the result of AMPK-regulated SIRT2 (Fig. 3A, B). Next, SIRT2 small interfering RNAs (siRNAs) were used to test whether AMPK activation-mediated deacetylation of CLYBL rely on the upregulation of SIRT2 (Fig. S2C). Our results showed that when SIRT2 is knocked down, the activation of AMPK using AICAR can no longer cause deacetylation of CLYBL compared to stimulated with AICAR alone (Fig. 3C). Collectively, these data suggest that SIRT2 acts as the bridge between AMPK phosphorylation and CLYBL acetylation.

Unexpectedly, the suppression of the acetylation status of CLYBL triggered by CLYBL-K154R increased the phosphorylation of AMPK following LPS treatment but did not change in the absence of LPS (Fig. 3D, E). Taken together, these results demonstrate that the inhibition of AMPK induces the hyperacetylation of CLYBL, which further facilitates the hypophosphorylation of AMPK driven by inflammatory stimuli. Our results reveal a previously undescribed positive feedback loop between AMPK and CLYBL acetylation mediated by TLRs.

### Inactivation of CLYBL-K154 hinders the activation of pro-inflammatory macrophages

As AMPK is known to regulate inflammation through macrophage metabolic reprogramming [26] and CLYBL-K154R could increase AMPK phosphorylation, we further investigated the functional significance of CLYBL acetylation in macrophages. We treated RAW264.7 macrophages with CLYBL siRNA to eliminate basal endogenous expression and compare the effects of exogenous CLYBL-WT and CLYBL-K154R more accurately. Under physiological conditions, NLRP3, an inflammasome in pro-inflammatory macrophages, is not affected by CLYBL acetylation. However, with LPS treatment, compared with CLYBL-WT, CLYBL-K154R markedly suppressed NLRP3 activation (Fig. 3F), with a concomitant decrease in IL-1β and IL-6 levels (Fig. 3G). Similarly, the decline in endogenous CLYBL via si-CLYBL attenuated the levels of NLRP3 and pro-inflammatory cytokines after treatment with LPS, further indicating the significant role of CLYBL in pro-inflammatory effects.

Activated macrophages produce pro-inflammatory cytokines, such as IL-6 and IL-1β, to initiate inflammation. Elevated NLRP3 activation is also observed in M1 macrophages [27]. To assess whether the anti-inflammatory effects of CLYBL-K154R are associated with the polarization of macrophages, macrophages were treated with LPS, and flow cytometry and immunofluorescence staining were used to detect the surface antigens CD86 and iNOS, both markers of the macrophage M1 type. The LPS-stimulated macrophages showed an upregulation of CD86, but CLYBL-K154R profoundly suppressed the expression of CD86 (Fig. 3H and Fig. S2D). Consistent with the flow cytometry results, the inactivation of CLYBL-K154 alleviated the increase in macrophage activation, as measured by iNOS expression (Fig. 3I and Fig. S2E). Collectively, these data suggest that the inactivation of CLYBL-K154 acetylation antagonizes the LPS-induced inflammatory response and does so, in part, by inhibiting macrophage polarization.

### Increased monocyte infiltration mediated by CLYBL hyperacetylation aggravates cardiac remodeling

**Fig. 4:**
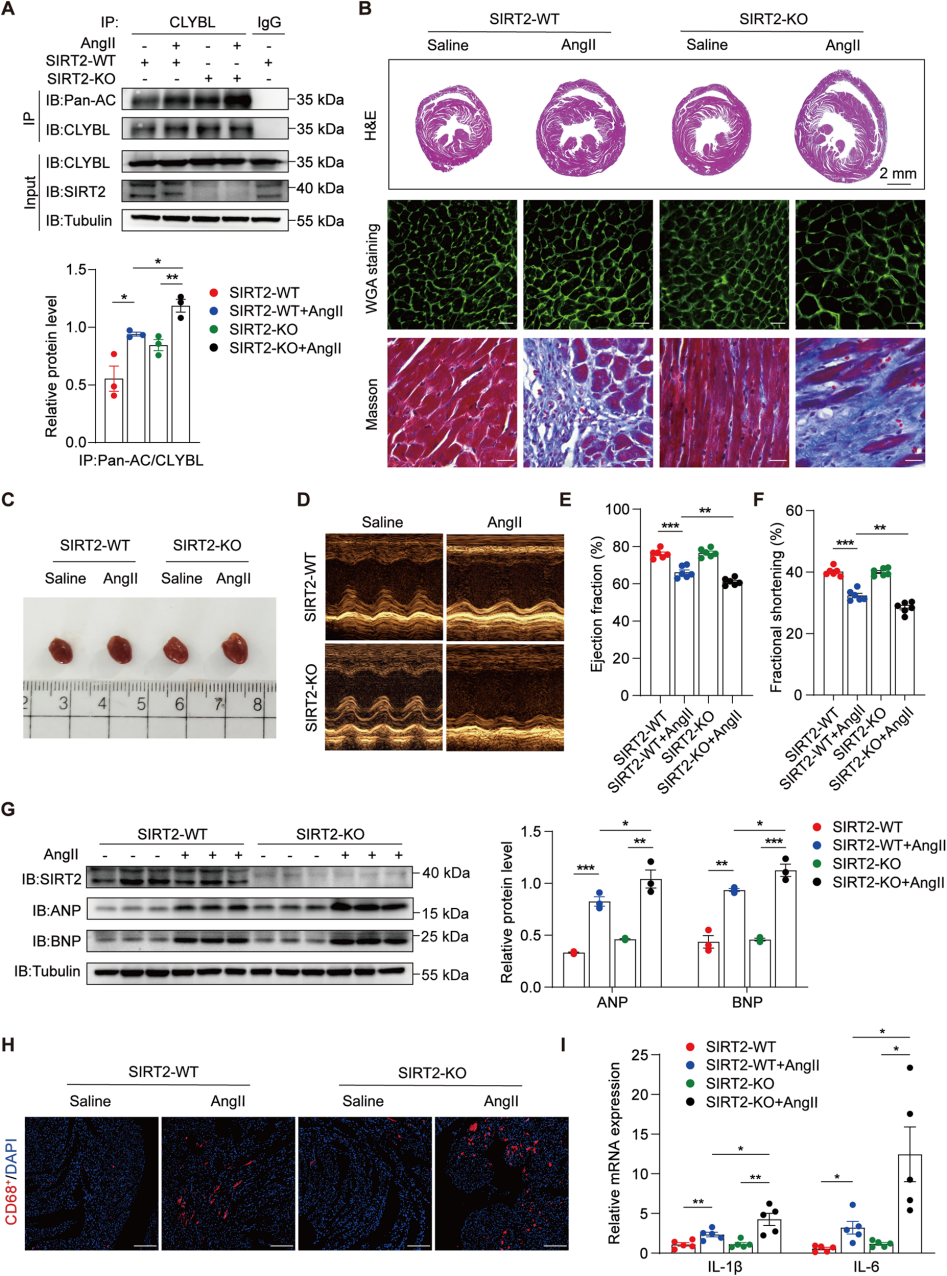
Increased monocyte infiltration mediated by CLYBL hyperacetylation aggravates cardiac remodeling. **A** Hearts from SIRT2-WT and SIRT2-KO mice injected with saline or Ang II (1.4 mg/kg/d) for 4 weeks were used to detect the acetylation of CLYBL (*p < 0.05; **p < 0.01). **B** H&E (upper), Scale bar, 2 mm; WGA (centered), Scale bar, 20 μm; and Masson’s trichrome staining (lower), Scale bar, 20 μm. **C** Measurements of representative heart specimens. **D** Echocardiogram was used to assess cardiac function. **E**, **F** Ejection fraction and fractional shortening in WT and SIRT2-KO mice treated with saline or Ang II (**p < 0.01; ***p < 0.001). **G** Expression levels of myocardial injury markers (ANP, BNP) were evaluated using western blotting (*p < 0.05; **p < 0.01; ***p < 0.001). **H** Immunofluorescence was used to detect CD68 (CD68 red, DAPI blue; Scale bar, 100 μm). **I** qPCR was used to assess the relative levels of IL-6 and IL-1β (*p < 0.05; **p < 0.01).

As hypertensive cardiac remodeling is often accompanied by macrophage infiltration and chronic inflammation, we investigated whether the hyperacetylation of CLYBL aggravates cardiac remodeling by promoting infiltration and associated inflammatory reactions. SIRT2-KO mice were used to mimic the hyperacetylation of CLYBL in vivo. After Ang II infusion for 4 weeks, CLYBL acetylation in both SIRT2-WT and SIRT2-KO heart tissues was markedly increased (Fig. 4A).

Next, we measured the histopathological alterations in the mouse hearts to assess the impact of CLYBL hyperacetylation on cardiac function. Hematoxylin and eosin (H&E) and wheat germ agglutinin (WGA) staining were used to identify the cross-sectional areas of cardiomyocytes. The degree of collagen disposition detected by Masson’s trichrome staining was significantly higher in SIRT2-KO mice administered Ang II than SIRT2-WT mice (Fig. 4B, C, S3A, S3B). In the CLYBL hyperacetylated state, echocardiography demonstrated an unaltered ejection fraction (EF%) and fractional shortening (FS%) in the physiological state, which significantly decreased following Ang II administration (Fig. 4D, E, F). Additionally, under the condition of CLYBL hyperacetylation, the markedly upregulated heart failure markers ANP and BNP were observed in the mice treated with Ang II (Fig. 4G).

Studies have shown that the infiltration of bone marrow-derived monocytes is increased in patients with hypertension, while cytokines released by infiltrated macrophages also induce local inflammation in the surrounding cardiac microenvironment, leading to cardiac remodeling and correlating with poor heart failure outcomes [28, 29]. To further determine whether CLYBL hyperacetylation aggravates cardiac remodeling by increasing infiltration and the associated inflammatory responses, we tracked the macrophage numbers and levels of inflammatory cytokines in the heart. The results suggested that Ang II elicited an increase in macrophage infiltration, labeled with CD68, in the hearts of WT mice. This effect was exacerbated by the hyperacetylation of CLYBL (Fig. 4H, S3C). The levels of the inflammatory cytokines IL-6 and IL-1β were markedly elevated in the SIRT2-KO mouse hearts treated with Ang II (Fig. 4I). Collectively, these results suggest that the hyperacetylation of CLYBL is accompanied by exacerbated cardiac remodeling, and increased pro-inflammatory factor release and immune cell recruitment mediated by hyperacetylated CLYBL are the core.

### Decreased monocyte infiltration triggered by CLYBL hypoacetylation mitigates cardiac remodeling

**Fig. 5:**
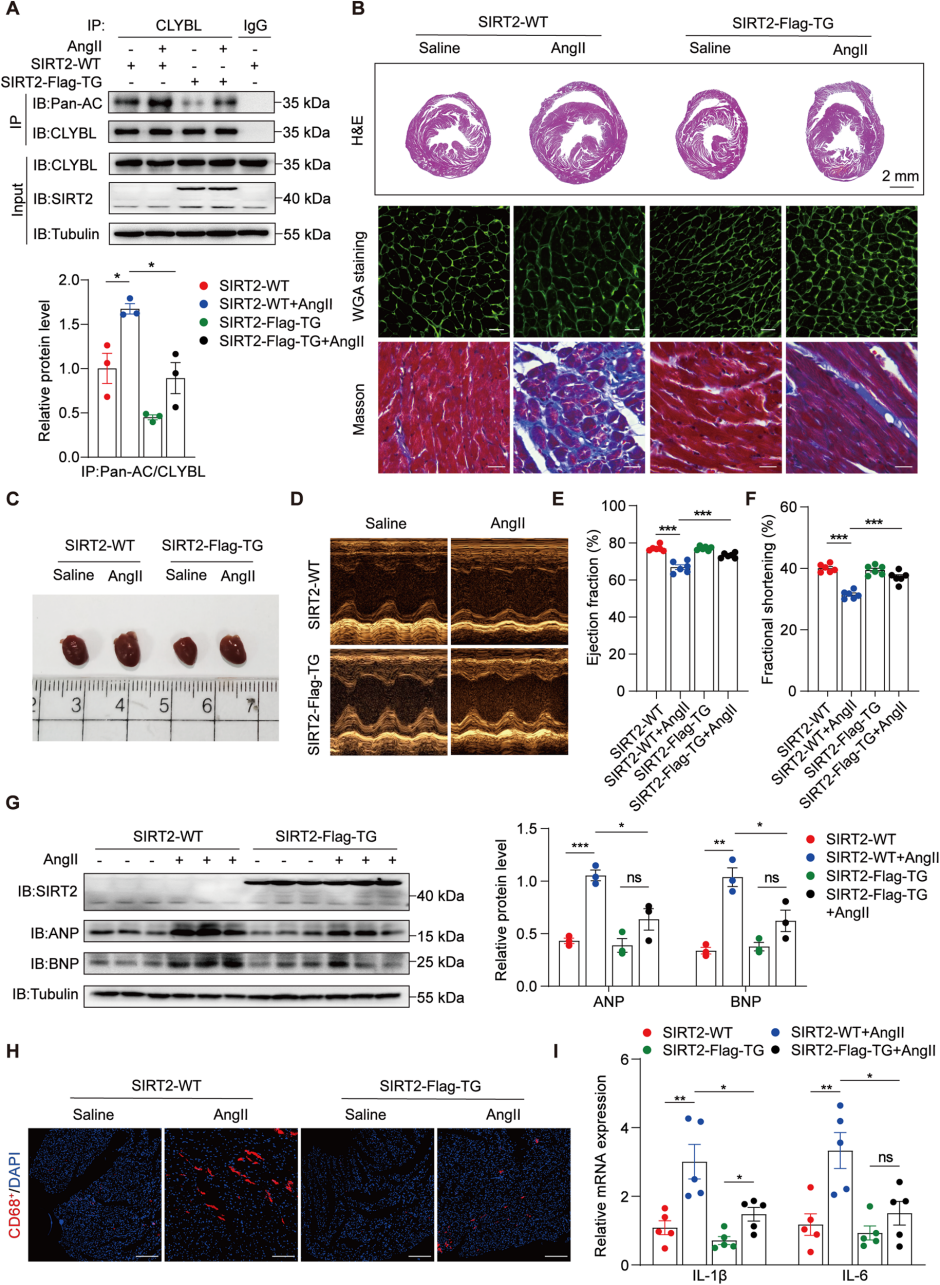
Decreased monocyte infiltration triggered by CLYBL hypoacetylation mitigates cardiac remodeling. **A** Myocardial tissue from SIRT2-WT and SIRT2-Flag-TG mice injected with saline or Ang II (1.4 mg/kg/d) for 4 weeks were used to detect the acetylation of CLYBL (*p < 0.05). **B** H&E (upper), Scale bar, 2 mm; WGA (centered), Scale bar, 20 μm; and Masson’s trichrome staining (lower), Scale bar, 20 μm. **C** Measurements of representative heart specimens. **D** Echocardiogram was used to assess cardiac function. **E**, **F** Ejection fraction and fractional shortening of SIRT2-Flag-TG and corresponding WT mice treated with saline or Ang II (***p < 0.001). **G** Expression levels of ANP and BNP were detected using western blotting (*p < 0.05; **p < 0.01; ***p < 0.001; ns, not significant). **H** Immunofluorescence was used to detect CD68 (CD68 red, DAPI blue; Scale bar, 100 μm). **I** qPCR was used to assess the relative levels of IL-6 and IL-1β (*p < 0.05; **p < 0.01; ns, not significant).

To further determine whether blocking CLYBL acetylation suppresses inflammatory responses and attenuates cardiac remodeling, we used SIRT2-Flag-TG to mimic the status of CLYBL hypoacetylation. After 4 weeks of Ang II treatment, CLYBL acetylation in both SIRT2-WT and SIRT2-Flag-TG heart tissues was increased, but the degree of CLYBL acetylation in SIRT2-Flag-TG hearts was lower than that in WT hearts (Fig. 5A).

In the Ang II-induced cardiac remodeling model, the cross-sectional area of myocardial cells was markedly decreased in the SIRT2-Flag-TG mice, and the same trend was observed in the degree of collagen configuration, as identified by Masson’s trichrome staining (Figs. 5B, C, S3D, S3E). In the CLYBL hypoacetylated state, echocardiography showed no difference in the EF% and FS% in the physiological state, but both were significantly alleviated in the pathological state (Fig. 5D–F). In addition, the hypoacetylation of CLYBL had a negative regulatory effect on the Ang II-induced upregulation of ANP and BNP protein expression (Fig. 5G).

The intrinsic mechanism by which hypoacetylated CLYBL mitigates Ang II-induced cardiac remodeling was further studied. The reduced infiltration of CD68-labeled macrophages (Fig. 5H, S3F) and expression of the pro-inflammatory cytokines IL-6 and IL-1β (Fig. 5I) were observed in the hypoacetylation state of CLYBL mimicked by SIRT2-Flag-TG mice. Taken together, these data further affirm the essential role of CLYBL acetylation in macrophage-triggered cardiac remodeling.

## Discussion

**Fig. 6:**
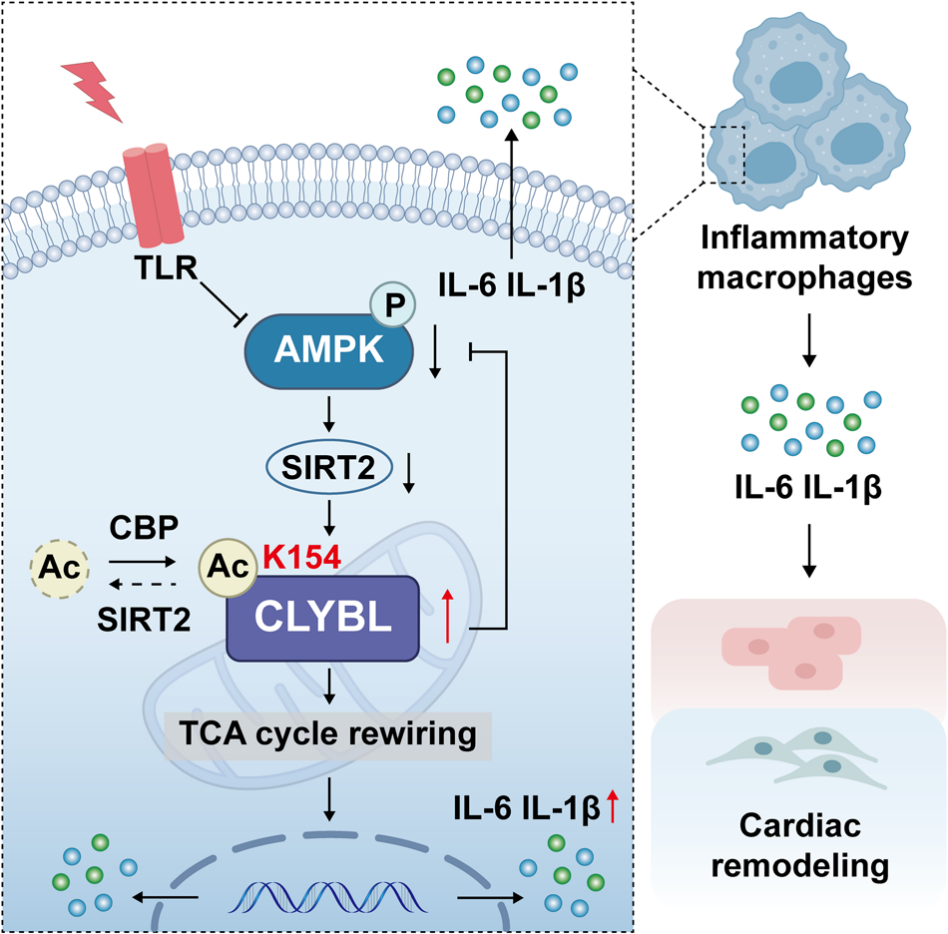
Working Model. External stimuli activate TLR signal, which impedes SIRT2 to deacetylate CLYBL and promotes pro-inflammatory cytokines production in an AMPK-dependent manner. In turn, hyperacetylation of CLYBL mediates hypophosphorylation of AMPK, which further promotes the hypercaetylation of CLYBL. The activation of this feedback loop increases IL-6 and IL-1β release through TCA cycle rewiring, leading to cardiac remodeling.

Metabolic reprogramming plays a pivotal role in macrophage activation in response to the disease microenvironment, but previous studies have mostly focused on alterations in macrophage functions and the mechanisms affected by the accumulation of metabolites [7, 11, 12]. The core mechanisms that trigger metabolite accumulation by TLRs have not been widely studied. Here, we discovered that a positive feedback loop between AMPK and CLYBL acetylation links metabolic rewiring and inflammatory responses triggered by TLRs. CLYBL is acetylated and deacetylated at K154 by CBP and SIRT2. Activated TLRs trigger the downregulation of AMPK phosphorylation, thus rendering a decline in the levels of the deacetylase SIRT2 and enhancing CLYBL acetylation. The inactivation of CLYBL at K154 (K154R) increases the level of AMPK phosphorylation, a phenomenon only observed in the pathological state, thereby hampering pro-inflammatory macrophage polarization. The AMPK hypophosphorylation-CLYBL hyperacetylation axis is reinforced by a positive feedback loop between the two, serving as a metabolic switch to enhance the effector functions of macrophages, leading to the increased production of pro-inflammatory cytokines. Using SIRT2-KO and SIRT2-Flag-TG mice to mimic states of increased and reduced CLYBL acetylation, we found that the hyperacetylation of CLYBL increased macrophage infiltration and inflammatory factors, thus exacerbating cardiac remodeling in mice, whereas the hypoacetylation of CLYBL exerted the opposite effect (Fig. 6). The discovery of the positive feedback loop of AMPK-CLYBL provides new insights into the effector functions of macrophages and metabolic rewiring. These findings further provide a theoretical basis for the inactivation of CLYBL-K154 as a possible therapeutic target for inflammatory response-related disorders.

Disease progression is often accompanied by inflammation. Hypertensive cardiac remodeling is characterized by chronic inflammation, cardiac hypertrophy, and fibrosis, which ultimately lead to heart failure [30, 31]. Increasing evidence has demonstrated that monocyte/macrophage inflammatory signaling is critically involved in cardiac remodeling [32–34]. Monocyte-derived macrophages are less abundant in the homeostatic heart but are recruited to the heart during aging or heart injury [35]. After exposure to stimuli, the injured heart promptly communicates with the spleen to respond to the damage signal evoked by Ang II, eliciting splenic monocytopoiesis and hematopoietic progenitor cell proliferation and leading to increased monocyte infiltration into the heart [36]. After recruitment to the heart, monocytes polarize into pro-inflammatory macrophages in response to DAMPs, and the released cytokines can interact with fibroblasts and cardiomyocytes, leading to cardiac remodeling and correlating with worse heart failure outcomes [34, 37, 38]. Therefore, identifying the mechanisms that drive the cardiac inflammatory response and curb macrophage infiltration and polarization may be crucial for the development of promising therapeutic strategies for cardiac remodeling.

With the discovery that activated macrophages suppress OXPHOS and dramatically rewire the TCA cycle, increasing evidence has suggested that the polarization of macrophages into diverse functional states relies on metabolic reprogramming [39]. The accumulation of TCA cycle metabolites, such as succinate, fumarate, and itaconate, is crucial for controlling the immunophenotype of macrophages [7, 11, 12]. Itaconate can inhibit IL-1β and IL-6 by regulating pathways such as the KEAP1/NRF2 axis and IκBζ-ATF3 inflammatory axis [40, 41]. Recently, with advancements in itaconate and immunometabolic research, the ubiquitously expressed mitochondrial enzyme CLYBL has garnered attention. In mammalian cells, CLYBL operates via the C5-dicarboxylate metabolic pathway, which catalyzes the transition from itaconate to acetyl-CoA. The three-step reaction involves the conversion of itaconate to acetyl-CoA. The first reaction converts itaconate to itaconyl-CoA. Hydrated itaconyl-CoA is then converted to citramalyl-CoA. In the final reaction, CLYBL acts as a citramalyl-CoA lyase to convert citramalyl-CoA to acetyl-CoA and pyruvate [13]. Therefore, CLYBL may aggravate inflammation via modulating itaconate. A clinical study also reported increased levels of the macrophage-enriched enzyme CLYBL in patients with COVID-19 [15]. Using quantitative acetylome profiling and co-immunoprecipitation, we identified CLYBL as a substrate of SIRT2 and found that CLYBL-K154 may be an important regulatory target in the immunometabolic control of macrophages.

Macrophage activation depends on the signaling pathways elicited by TLRs [16]. Stimulation with LPS, a TLR agonist, induces inflammatory responses in macrophages by activating transcriptional cascades and promoting macrophage polarization to the pro-inflammatory M1 phenotype [42]. Furthermore, concurrent changes in cellular metabolism occur upon TLR activation to rapidly supply energy, and this metabolic conversion is characterized by a switch from oxidative phosphorylation to aerobic glycolysis [24]. The metabolic switch must be driven by metabolism-related signaling pathway, which guide us to keep eyes on the conserved cellular energy sensor AMPK. AMPK regulates energy homeostasis by sensing the ratios of AMP and ADP to ATP [17]. An imbalance in the AMP/ATP ratio, triggered by increased aerobic glycolysis, results in AMPK hypophosphorylation, thereby weakening the negative regulatory ability of AMPK in aerobic glycolysis [43, 44]. The activation of AMPK in immune cells inhibits the inflammatory response, partly by causing a switch away from the conversion of aerobic glycolysis toward mitochondrial oxidative [24, 45]. Furthermore, the regulation of peripheral monocyte numbers is dependent on activated AMPK, which reduces monocyte mobilization from the bone marrow [46]. This finding emphasizes the undervalued role of AMPK as an effector in inflammatory response-related disorders. Our study demonstrated that TLRs can regulate metabolic reprogramming via the AMPK-CLYBL acetylation axis and that the altered metabolic switch affects AMPK activity under pathological conditions.

In summary, the positive feedback loop of AMPK and CLYBL acetylation links metabolic rewiring and inflammatory responses, and the inhibition of this loop may suppress inflammatory processes and pathological cellular functions. The homozygous loss of CLYBL is tolerated in healthy individuals [14], and given that CLYBL acetylation does not affect the level of protein expression, CLYBL-154 may be targeted as an immunomodulator, without causing side effects.

## Materials and methods

### Animals

SIRT2-KO mice were a gift from Deng [47]. Rosa26-SIRT2-Flag-TG mice were constructed by Shanghai Model Organisms Center. For all murine experiments, certified pathogen-free male mice aged 8–10 weeks were evaluated. In the Ang II- or sodium chloride (NaCl)-infused murine models, SIRT2-WT, SIRT2-KO, and SIRT2-Flag-TG mice (aggregate *n* = 48, *n* = 6 per group) underwent randomization. Cardiac remodeling was induced through the protracted subcutaneous administration of Ang II, reaching a dosage of 1.4 mg/kg/day, dissolved in physiological saline over a duration of 4 weeks [33]. Ultrasound was used to detect cardiac EF% and FS% in the mice. The Animal Subjects Committee of China Medical University approved all animal experiments (license no. CMU20231452).

### Proteomics and acetylation proteomics

Proteomic and acetylation data were supplied by Jingjie PTM BioLab (Hangzhou, China). Samples consisted of cardiac tissues from SIRT2-KO, SIRT2-Flag-TG mice and WT mice treated with Ang II (*n* = 3). Cardiac tissues were sonicated to extract proteins and total protein in each sample was enzymatically digested to generate tryptic peptides. Peptides were dissolved in IP buffer and incubated overnight at 4°C with anti-lysine acetylation remnant antibody resins to enrich for post-translational modified peptides. Then, tryptic peptides were dissolved in liquid chromatographic mobile phase A and separated on a NanoElute ultra-high performance liquid system. Mobile phases A and B included 0.1% formic acid and 2% acetonitrile (diluted in water) and 0.1% formic acid (diluted in acetonitrile). Peptides were eluted using the following gradient: 0–40 min, 6–24% B; 40–52 min, 24–32% B; 52–56 min, 35–80% B; 56–60 min, 80% B with a constant flow rate at 450 nL/min. Separated peptides were injected into a capillary ion source for ionization and TIMS-TOF Pro mass spectrometer for LC-MS/MS analysis subsequently. Raw mass spectrometry data was searched against a Swissprot protein sequence database by Maxquant (v1.6.15.0), UniProt-GOA database was utilized for GO annotation and Encyclopedia of Genes and Genomes (KEGG) database was used to analyze enrichment pathways. Specific methodologies are described in a previously published article [48].

### Cell culture

RAW264.7 cells and HEK293T cells procured from HyCyte™ underwent cultivation in High Glucose Dulbecco’s Modified Eagle Medium (DMEM), complemented with 10% fetal bovine serum, 1% penicillin and streptomycin. Cells were cultured in a humidified atmosphere containing 5% CO_2_ and maintained at 37 °C. All cell lines were authenticated using short tandem repeat (STR) profiling and regularly tested to ensure they were free of mycoplasma contamination.

### Plasmids and siRNAs construction

The synthesis of plasmids encompassing complete CLYBL sequences, SIRT2 sequences, mutated SIRT2 sequences, CBP sequences, P300 sequences, and PCAF sequences involved their integration into Flag-tagged destination vectors. Full-length SIRT2 and GCN5 sequences were integrated into destination vectors containing Myc tag. Lysine-mutated CLYBL variants (K307R, K154R, and K55R) were incorporated into the Flag-vector provided by Gene Chemistry. siRNAs were purchased from RIBOBIO and Huzhou Hippo Biotechnology Co., Ltd. Three target sequences were employed for CLYBL and SIRT2 gene targeting to mitigate potential off-target effects. The efficacy of CLYBL and SIRT2 knockdown was confirmed using western blotting. The specific target sequences are listed in the Table S1.

### Cell transfection and treatment

The plasmids were transfected into cells with the HiGene I transfection reagent (Applygen, China), following the manufacturer’s recommendations (plasmid DNA/transfection reagent ratio =1 μg/1.5 μL). siCLYBL and siSIRT2 were transfected with the jetPRIME transfection reagent (siRNA/transfection reagent ratio = 20 pmol/μL) procured from PolyPlus. Cells were harvested at intervals of 48 h or 72 h post-transfection. 293 T cells were subjected to treatment with 0.5 μM TSA for 16 h and 5 mM NAM for 4 h. A distinct set of 293 T cells underwent treatment with AGK2, a specific inhibitor of SIRT2, at a concentration of 20 μM for a duration of 24 h to investigate the SIRT2-induced deacetylation of CLYBL. RAW264.7 cells were induced into a pro-inflammatory state through exposure to LPS at a concentration of 1 μg/mL for a duration of 8 h. For some experiments, RAW264.7 cells were treated with AICAR. The optimal concentration for AICAR treatment in RAW264.7 cells was determined to be 500 μM, with a treatment duration of 6 h.

### Histology staining

Cardiac samples underwent fixation in a 4% paraformaldehyde solution, embedded in paraffin, and sectioned to a thickness ranging from 4 to 5 μm. Before staining, the sections of the cardiac tissue were dewaxed using xylene, dehydrated by progressively decreasing ethanol concentrations, rinsed with PBS, and subsequently stained with H&E, WGA, and Masson’s trichrome.

### Immunofluorescence staining

After dewaxing and dehydration, the cardiac tissue sections were boiled in Tris-EDTA solution for antigen retrieval. Then, the samples were washed three times with PBS and treated with 0.3% Triton X-100 for 15 min. After washing three times with PBS, the samples were blocked with 5% Bovine Serum Albumin (BSA) for 1 h, and then incubated with an anti-CD68 (cat: 28058-1-AP, Proteintech, 1:300) overnight. Followed by washing with PBS three times, the sections were incubated with its secondary antibody: Donkey anti-Rabbit IgG (H + L) Highly Cross-Adsorbed Secondary Antibody (cat: A-21207, Thermo Fisher Scientific, 1:500). RAW264.7 cells were fixed with 4% paraformaldehyde solution and permeabilized for 15 min with 0.2% Triton X-100. Following a 1 h blocking step with 5% BSA, the samples were incubated at 4 °C overnight with primary antibody: anti-iNOS (cat: 18985-1-AP, Proteintech, 1:200). Subsequently, the cells were treated with secondary antibodies labeled with Alexa Fluor.

### Western blotting and immunoprecipitation

To extract the proteins, cellular and tissues were lysed in IP lysis buffer [49]. Subsequently, the mixtures underwent centrifugation at 13,300 rpm for 20 min at 4 °C and protein samples were quantified. For immunoprecipitation, cell lysates were incubated with antibodies (antibody/cell lysate=1 μg/mg) for 3 h, followed by incubation with 25 μL of protein A/G immunoprecipitation magnetic beads or anti-Flag Affinity Gel for 12 h at 4 °C and the complexes were washed three times with cold IP lysis buffer. For western blotting and immunoprecipitation, samples were separated by 6%, 8%, or 10% SDS-PAGE, followed by transfer to PVDF membranes. The membranes were blocked for 1 h with 5% BSA in TBST buffer and then incubated with specific primary antibodies at 4 °C overnight. After washing three times with TBST, the membranes were incubated with secondary antibodies at room temperature for 1 h. The quantification of the western blotting results involved the utilization of a minimum of three blots, and the outcomes were presented as the mean ± standard error of the mean (SEM). Antibodies were used as follows: anti-CLYBL (cat: 17314-1-AP, Proteintech, 1:2000), anti-SIRT2 (cat: ab51023, Abcam, 1:1000), anti-Flag (cat: GNI4110-FG, GNI, 1:1000), anti-Myc (cat: GNI4110-MC, GNI, 1:1000), anti-AMPK (cat: 2532S, Cell Signaling Technology, 1:1000), anti-p-AMPK (cat: 2535S, Cell Signaling Technology, 1:1000), anti-Acetylated-Lysine (cat: 9441S, Cell Signaling Technology, 1:1000), anti-CBP (cat: 7389S, Cell Signaling Technology, 1:1000), anti-NLRP3 (cat: 15101S, Cell Signaling Technology, 1:1000), and anti-ANP (cat: DF6497, Affinity Biosciences, 1:1000), anti-BNP (cat: DF6902, Affinity Biosciences, 1:1000), anti-α-Tubulin (cat: 11224-1-AP, Proteintech, 1:2000), anti-GAPDH (cat: 60004-1-Ig, Proteintech, 1:2000), (HRP)-conjugated goat anti-Mouse IgG (cat: A21010, Abbkine, 1:7000), (HRP)-conjugated goat anti-Rabbit IgG (cat: A21020, Abbkine, 1:7000).

### Quantitative real-time PCR

Total RNA was extracted from cardiac tissues or RAW264.7 by RNAiso Plus. A total of 500 ng of RNA was reverse transcribed into cDNA within a reaction volume of 20 μL by using the PrimeScript™ RT Reagent kit, according to the manufacturer’s instructions. Quantitative real-time PCR reactions were conducted using TB Green Premix Ex Taq™ II on the LightCycler96 System. The PCR primers are listed in the key resources table.

### Flow cytometry

Each sample with 100 μL binding buffer contained 10^6^ RAW264.7 cells. Before surface staining, the cells were treated with TruStain FcX™ (CD16/32) antibody (cat: 101319, BioLegend, 1 μg/test) at 4 °C for 10 min. To detect the cell surface antigens, the cells were treated with CD86 (cat: 159202, BioLegend, 1 μg/test) at 4 °C for 30 min. Before flow cytometry, the cells were washed twice with cell staining buffer. All data were collected using NovoExpress 1.5.0 on a NovoCyte.

### Statistical analysis

The data are presented as the mean ± SEM or the percentage derived from a minimum of three independent experiments, employing rigorous statistical analyses through GraphPad Prism 8.0. Homogeneity of variance was evaluated using the F-test in the case of two groups or the Brown-Forsythe test for three or more groups. Comparisons involving equal variance for two groups were conducted using Student’s *t*-tests, and Welch’s *t* test was employed in cases of unequal variance. Comparisons among multiple groups were conducted using a one-way analysis of variance supplemented by Tukey’s comparison test for a post hoc analysis. The criterion for statistical significance was set at *p* < 0.05.

## Supplementary information

Supplemental figure 1

Supplemental figure 2

Supplemental figure 3

Table S1

Supplementary legends

Uncropped Western blots

## Data Availability

All data are available in the main text or the supplementary materials.
