# Supplementary figures and images for: Positive feedback loop involving AMPK and CLYBL acetylation links metabolic rewiring and inflammatory responses
Source: Cell Death Dis. 2025 Jan 25;16(1):41. doi: 10.1038/s41419-025-07362-0 (PMC11762313; doi:10.1038/s41419-025-07362-0)

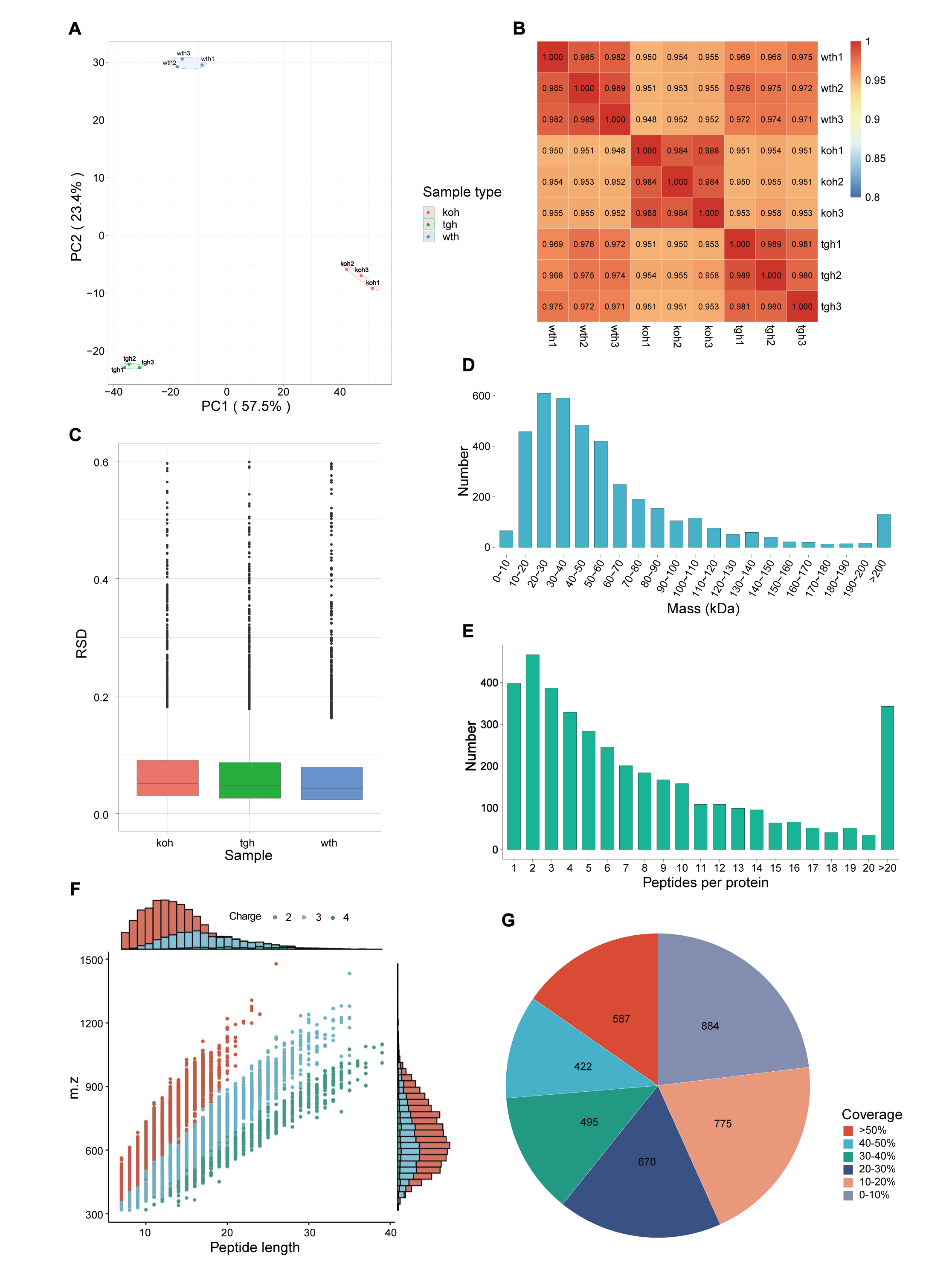

Supplement: Supplementary file 1 — Supplemental figure 1 [file 41419_2025_7362_MOESM1_ESM.tif]

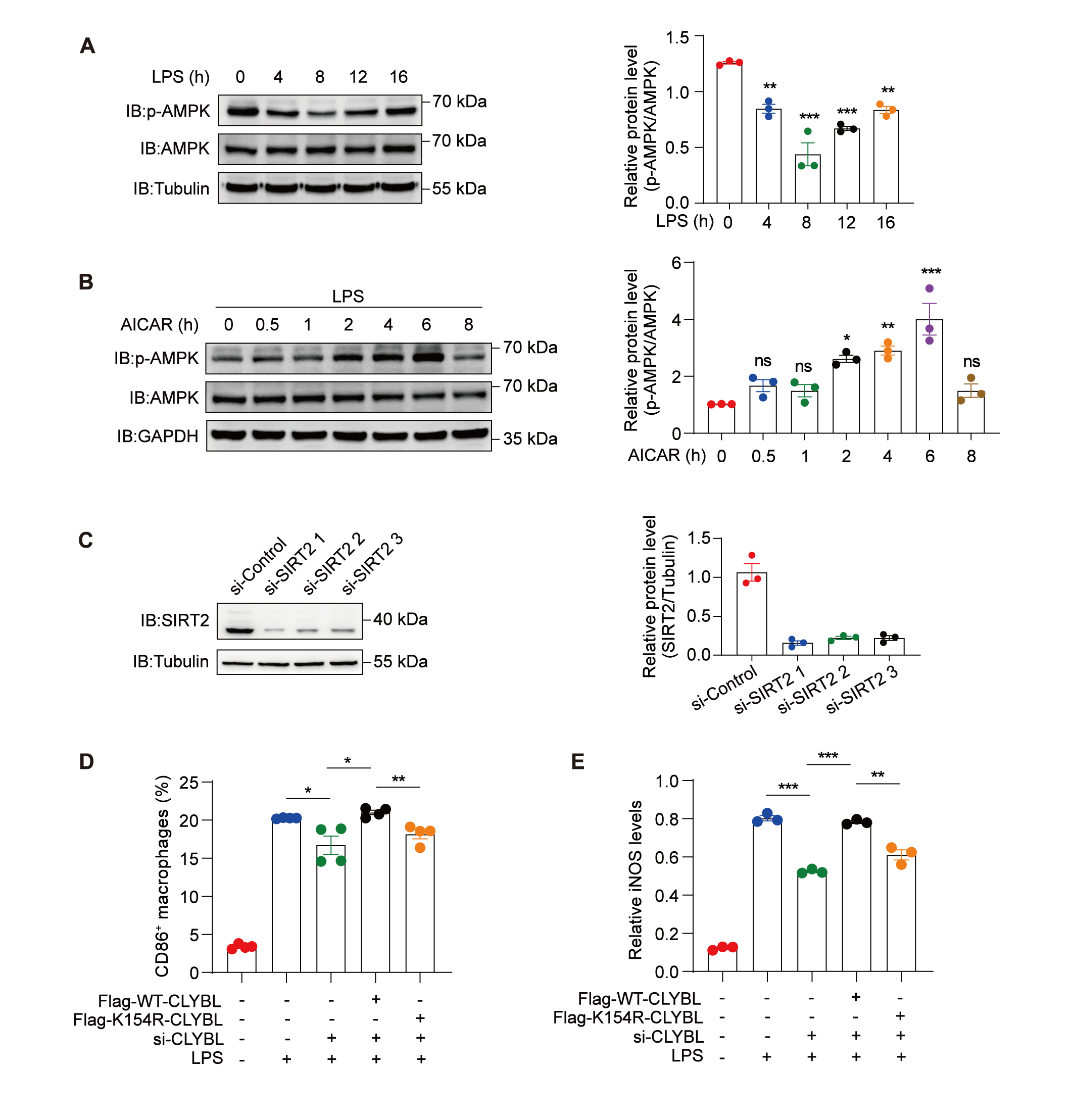

Supplement: Supplementary file 2 — Supplemental figure 2 [file 41419_2025_7362_MOESM2_ESM.tif]

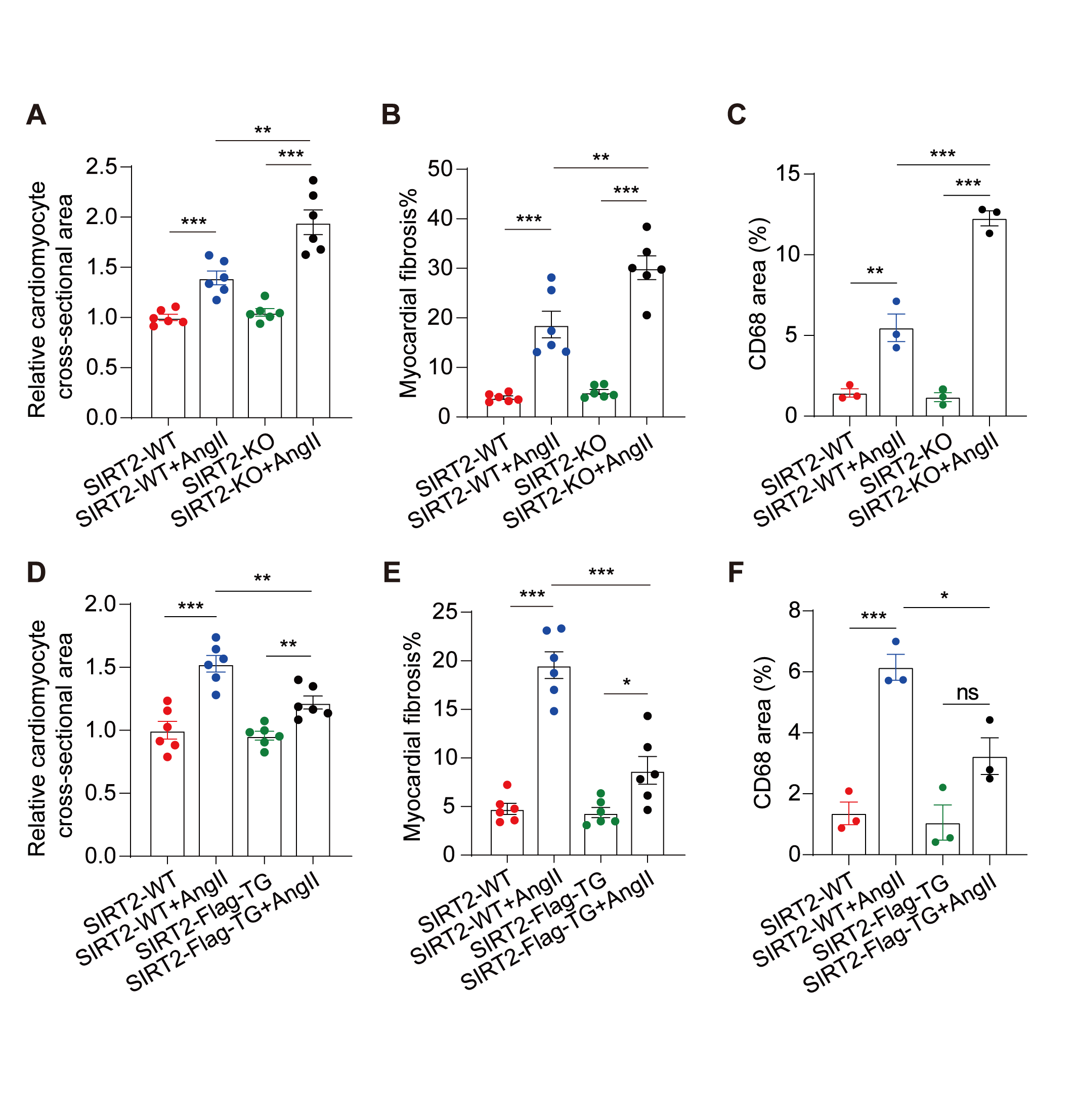

Supplement: Supplementary file 3 — Supplemental figure 3 [file 41419_2025_7362_MOESM3_ESM.tif]
