# Supplementary material for: Positive feedback loop involving AMPK and CLYBL acetylation links metabolic rewiring and inflammatory responses
Source: Cell Death Dis. 2025 Jan 25;16(1):41. doi: 10.1038/s41419-025-07362-0 (PMC11762313; doi:10.1038/s41419-025-07362-0)
Supplement: Supplementary file 4 — Table S1 [file 41419_2025_7362_MOESM4_ESM.docx]

Table S1. The key resources and reagents used in the present study

| REAGENT or RESOURCE | SOURCE | IDENTIFIER |
| --- | --- | --- |
| Antibodies for western blotting | | |
| Rabbit anti-CLYBL (1:2000) | Proteintech | Cat# 17314-1-AP  RRID: AB_2083483 |
| Rabbit anti-SIRT2 (1:1000) | Abcam | Cat# ab51023  RRID: AB_882563 |
| Mouse anti-Flag (1:1000) | GNI | Cat# GNI4110-FG  RRID: AB_3067995 |
| Mouse anti-Myc (1:1000) | GNI | Cat# GNI4110-MC  RRID: AB_3076243 |
| Rabbit anti-AMPK (1:1000) | Cell Signaling Technology | Cat# 2532S  RRID: AB_330331 |
| Rabbit anti-p-AMPK (1:1000) | Cell Signaling Technology | Cat# 2535S  RRID: AB_331250 |
| Rabbit anti- Acetylated-Lysine (1:1000) | Cell Signaling Technology | Cat# 9441S  RRID: AB_331805 |
| Rabbit anti-CBP (1:1000) | Cell Signaling Technology | Cat# 7389S  RRID: AB_2616020 |
| Rabbit anti-NLRP3 (1:1000) | Cell Signaling Technology | Cat# 15101S  RRID: AB_2722591 |
| Rabbit anti-ANP (1:1000) | Affinity Biosciences | Cat# DF6497  RRID: AB_2838459 |
| Rabbit anti-BNP (1:1000) | Affinity Biosciences | Cat# DF6902  RRID: AB_2838861 |
| Rabbit anti-α-Tubulin (1:2000) | Proteintech | Cat# 11224-1-AP  RRID: AB_2210206 |
| Mouse anti-GAPDH (1:2000) | Proteintech | Cat# 60004-1-Ig  RRID: AB_2107436 |
| HRP, Goat Anti-Mouse IgG (1:7000) | Abbkine | Cat# A21010  RRID: AB_2728771 |
| HRP, Goat Anti-Rabbit IgG (1:7000) | Abbkine | Cat# A21020  RRID: AB_2876889 |
| Antibodies for histology and immunofluorescence staining | | |
| Rabbit anti-iNOS (1:200) | Proteintech | Cat# 18985-1-AP  RRID: AB_2782960 |
| Rabbit anti-CD68 (1:300) | Proteintech | Cat# 28058-1-AP  RRID: AB_2881049 |
| Donkey anti-Rabbit IgG (H+L) Highly. Cross-Adsorbed Secondary Antibody, Alexa Fluor™ 594 (1:500) | Thermo Fisher Scientific | Cat# A-21207  RRID: AB_141637 |
| Antibodies for flow cytometry | | |
| Mouse anti-CD86 | BioLegend | Cat# 159202  RRID: AB_2860748 |
| Mouse anti-CD16/32 | BioLegend | Cat# 101319  RRID: AB_1574973 |
| Reagents | | |
| Anti-Flag Affinity Gel | Selleck | Cat# B23102 |
| Protein A/G Magnetic Beads | Selleck | Cat# B23202 |
| Protease Inhibitor Cocktail | Selleck | Cat# B14001 |
| LPS | Sigma | Cat# L3012 |
| AGK2 | MedChemExpress | Cat# HY-100578 |
| AICAR | TOCRIS | Cat# 2840 |
| Trichostatin A | Selleck | Cat# S1045 |
| Nicotinamide | Selleck | Cat# S1899 |
| HiGene I transfection reagent | Applygen | Cat# C1506 |
| jetPRIME | PolyPlus | Cat# Polyplus_PT-114-15 |
| Dulbecco’s Modified Eagle’s Medium. (DMEM) | Viva Cell Biosciences | Cat# C3103-0500 |
| Certified Fetal Bovine Serum | Viva Cell Biosciences | Cat# C04001-500 |
| Dulbecco's Phosphate Buffered Saline. (PBS) | Viva Cell Biosciences | Cat# C3593-0500 |
| Penicillin-Streptomycin Solution | HyCyte™ | Cat# GUSA-R002 |
| Bovine Serum Albumin | Solarbio | Cat# A8020 |
| 4% Paraformaldehyde | Solarbio | Cat# P1110 |
| Tris-EDTA Antigen Retrieval Solution | Solarbio | Cat# C1038 |
| 4′,6-diamidino-2-phenylindole | Solarbio | Cat# C0065 |
| Antifade Mounting Medium | Beyotime | Cat# P0126 |
| RNAiso Plus | TAKARA | Cat# 9108 |
| Critical commercial assays | | |
| BCA protein assay | Solarbio | Cat# PC0020 |
| PrimeScript™ RT reagent Kit with. gDNA Eraser (Perfect Real Time) | TAKARA | Cat# RR047A |
| TB Green Premix Ex Taq™ II  (Tli RNaseH Plus) | TAKARA | Cat# RR820A |
| Hematoxylin and Eosin (HE) | Solarbio | Cat# G1120 |
| Wheat Germ Agglutinin (WGA) | Vectorlabs | Cat# RL-1022 |
| Masson's Trichrome Stain Kit | Solarbio | Cat# G1340 |
| Experimental models: Cell lines | | |
| HEK293T | HyCyte™ | Cat# TCH-C101 |
| RAW264.7 | HyCyte™ | Cat# TCM-C766 |

| Experimental models | | | | | | | | | | |
| --- | --- | --- | --- | --- | --- | --- | --- | --- | --- | --- |
| SIRT2-KO mice | | | A gift from Chuxia Deng | | | | | PMID: 22014574 | | |
| SIRT2-TG mice | | | Shanghai Biomodel Organism Science &. Technology Development | | | | | N/A | | |
| Recombinant DNA | | | | | | | | | | |
| Flag-WT-CLYBL | | | | GeneChem (China) | | | | Cat# GOSE0330416 | | |
| Flag-K307R-CLYBL | | | | GeneChem (China) | | | | Cat# GOSE0330419 | | |
| Flag-K154R-CLYBL | | | | GeneChem (China) | | | | Cat# GOSE0330418 | | |
| Flag-K55R-CLYBL | | | | GeneChem (China) | | | | Cat# GOSE0330417 | | |
| Flag-WT-SIRT2 | | | | A gift from Prof.Chuangui Wang | | | | N/A | | |
| Myc-WT-SIRT2 | | | | GeneChem (China) | | | | Cat# GOSE0255332 | | |
| Flag-Q167AH187Y-SIRT2 | | | | A gift from Prof.Chuangui Wang | | | | N/A | | |
| Flag-CBP | | | | A gift from Prof.Qunying Lei. (Shanghai Medical College, Shanghai, China) | | | | N/A | | |
| Myc-GCN5 | | | | A gift from Prof.Qunying Lei | | | | N/A | | |
| Flag-P300 | | | | A gift from Prof.Qunying Lei | | | | N/A | | |
| Flag-PCAF | | | | A gift from Prof.Qunying Lei | | | | N/A | | |
| Software and algorithms | | | | | | | | | | |
| GraphPad Prism 8.0 | | | | | Software | | http://www.graphpad.com | | | |
| ImageJ | | | | | Software | | https://imagej.net/ij/download.html | | | |
| Adobe Illustrator | | | | | Software | | https://www.adobe.com | | | |
| NovoExpress 1.5.0 | | | | | Software | | <https://www.agilent.com.cn/zh-cn/product/research->  flow-cytometry/flow-cytometry-software/novocyte-  novoexpress-software-1320805 | | | |
| Leica Application Suite X 3.7.4 | | | | | Software | | https://www.leica-microsystems.com/products/  microscope-software/ | | | |
| NIS-Elements Viewer 5.21 | | | | | Software | | <https://www.microscope.healthcare.nikon.com/products/>software/nis-elements/viewer | | | |
| Primers for RT-PCR | | | | | | | | | | |
| Primer | FOWARD | | | | | REVERSE | | | | SOURCE |
| IL-6 | 5’-CCACTTCACAA GTCGGAGGCTTA-3’ | | | | | 5’- GCAAGTGCATC  ATCGTTGTTCATAC-3’ | | | | Sangon Biotech  (Shanghai, China) |
| IL-1β | 5’-TCCAGGATGAG  GACATGAGCAC-3’ | | | | | 5’- GAACGTCACAC  ACCAGCAGGTTA-3’ | | | | Sangon Biotech  (Shanghai, China) |
| GAPDH | 5’-GGTTGTCTCCTG  CGACTTCA-3’ | | | | | 5’-TGGTCCAGGGTT  TCTTACTCC-3’ | | | | Sangon Biotech  (Shanghai, China) |
| siRNA | | | | | | | | | | |
| siRNA | | SPECIES | | | TARGET SEQUENCE | | | | SOURCE | |
| siCLYBL 001 | | Mouse | | | CTGGTAAACAGGTGATCCA | | | | RIBOBIO (China) | |
| siCLYBL 002 | | Mouse | | | GCCTACAAGCCATAGATCT | | | | RIBOBIO (China) | |
| siCLYBL 003 | | Mouse | | | GTATGATCGACATGCCATT | | | | RIBOBIO (China) | |
| siSIRT2 001 | | Mouse | | | CCAGAATAAGGCATTTCTCTA | | | | Huzhou Hippo Biotechnology Co., Ltd. | |
| siSIRT2 002 | | Mouse | | | TGGCTAAATCAAATTAACCTA | | | | Huzhou Hippo Biotechnology Co., Ltd. | |
| siSIRT2 003 | | Mouse | | | CACGGCUGCUCAUUAACAATT | | | | Huzhou Hippo Biotechnology Co., Ltd. | |
