# Supplementary material for: Positive feedback loop involving AMPK and CLYBL acetylation links metabolic rewiring and inflammatory responses
Source: Cell Death Dis. 2025 Jan 25;16(1):41. doi: 10.1038/s41419-025-07362-0 (PMC11762313; doi:10.1038/s41419-025-07362-0)
Supplement: Supplementary file 5 — Supplementary legends [file 41419_2025_7362_MOESM5_ESM.docx]

**Supplemental Materials**

**Supplemental Fig. 1. Quality control results of the SIRT2 proteome.**

(**A**) Principal component analysis showing the first three principal components of protein intensities, with specimens linked by centroids based on the sample type. (**B**) Pearson’s correlation analysis. Each value represents the correlation coefficient between the three samples. (**C**) Boxplot based on the relative standard deviation (RSD), with each point representing an RSD. (**D**) Protein molecular weight statistics; bar height represents the protein number. (**E**) Peptide length statistics after enzymatic hydrolysis, with the bar height representing the number of peptides. (**F**) Tolerance distribution of the peptide mass. (**G**) Protein coverage is shown in pie charts.

**Supplemental Fig 2. Activation of TLRs suppresses SIRT2 expression via AMPK signaling in macrophages.**

(**A**) The protein expression levels of p-AMPK and AMPK were measured after stimulation with LPS at doses of 1 μg/mL for different times in RAW264.7 cells (***p*<0.01, ****p*<0.001). (**B**) The protein expression levels of p-AMPK and AMPK were assessed following stimulation with a combination of LPS (1 μg/mL, 8 h) and AICAR at a concentration of 500 μM for various durations in RAW264.7 cells (**p*<0.05; ***p*<0.01; ****p*<0.001; ns, not significant). **(C)** The expression of SIRT2 was assessed after transfection with NC, siSIRT2-1, siSIRT2-2, and siSIRT2-3 in RAW264.7 cells. (**D**) Percentages of CD86-positive fractions were assessed using flow cytometry (**p*<0.05; ***p*<0.01). (**E**) The relative expression of iNOS was examined using immunofluorescence staining (***p*<0.01; ****p*<0.001).

**Supplemental Fig 3. SIRT2 regulates Ang II-induced cardiac remodeling.**

(**A**-**B**) Quantification of relative cardiomyocyte cross-sectional area (A) and percentages of the fibrotic fraction (B) (***p*<0.01; ****p*<0.001). (**C**) Quantification of the levels of CD68-positive area in Figure 4H (***p*<0.01; ****p*<0.001). (**D**-**E**) Quantification of relative cardiomyocyte cross-sectional area (D) and percentages of the fibrotic fraction (E) (**p*<0.05; ***p*<0.01; ****p*<0.001). (**F**) Quantification of the levels of CD68-positive area in Figure 5H (**p*<0.05; ****p*<0.001; ns, not significant).
