# Supplementary material for: Positive feedback loop involving AMPK and CLYBL acetylation links metabolic rewiring and inflammatory responses
Source: Cell Death Dis. 2025 Jan 25;16(1):41. doi: 10.1038/s41419-025-07362-0 (PMC11762313; doi:10.1038/s41419-025-07362-0)

Uncropped Western blots for Figure 2

Uncropped Western blots for Figure 2 A

IP: CLYBL@SIRT2 IB: SIRT2

IP: CLYBL@SIRT2 IB: CLYBL

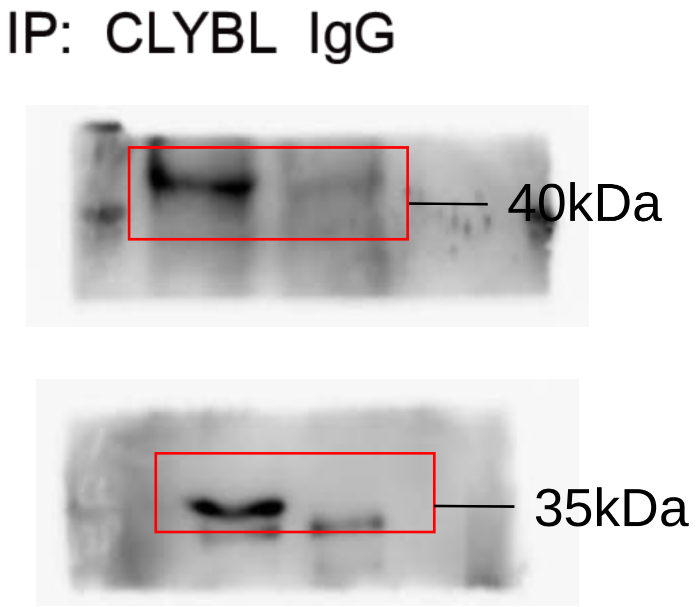

Uncropped Western blots for Figure 2 A

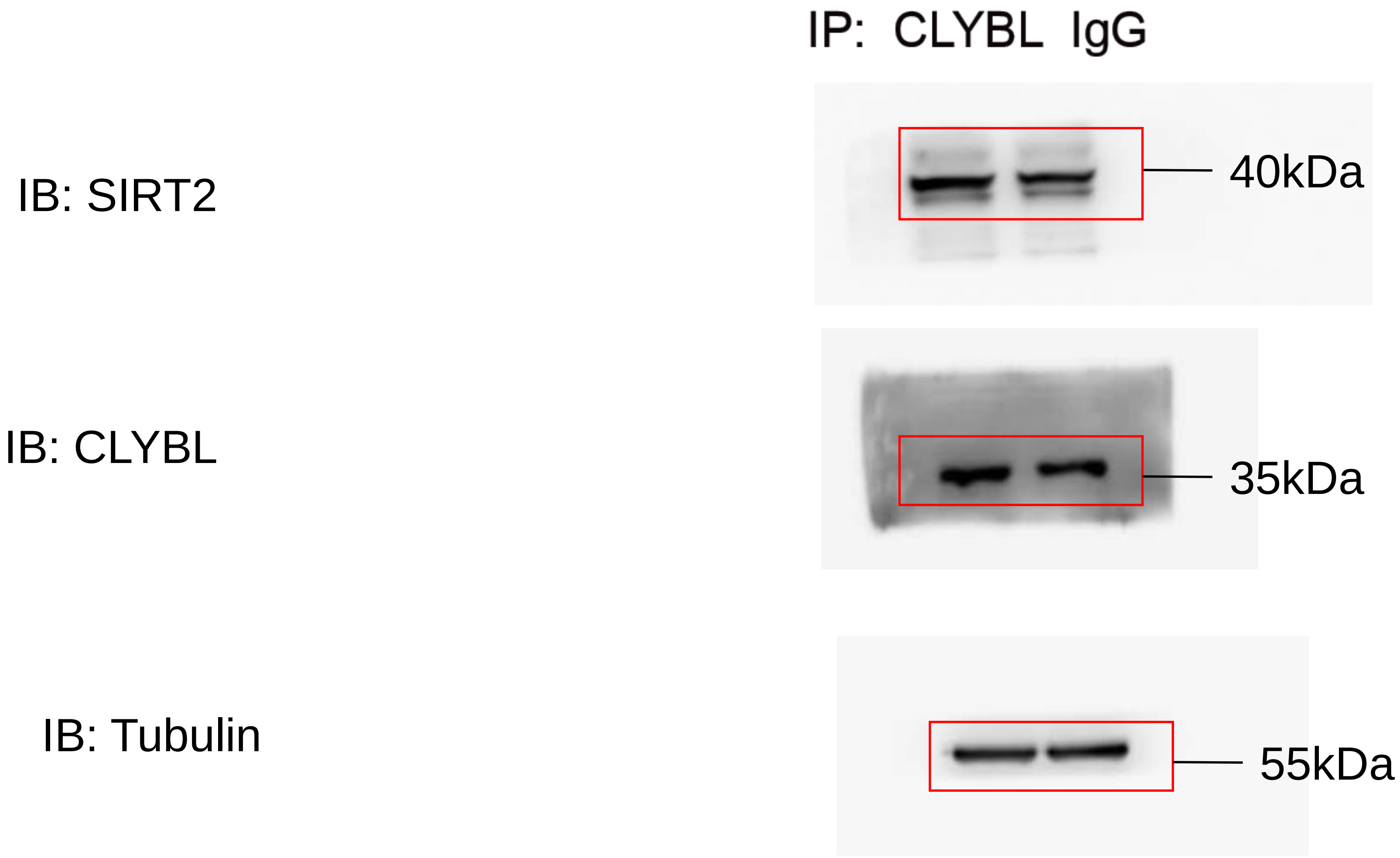

Uncropped Western blots for Figure 2 B

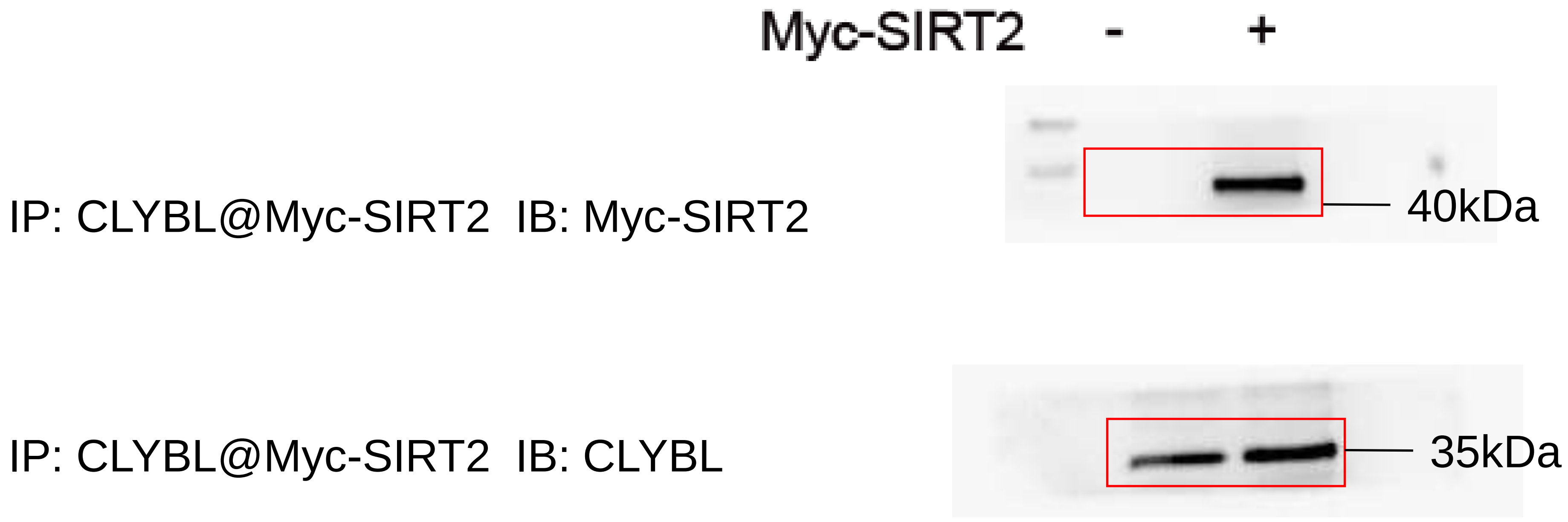

Uncropped Western blots for Figure 2 B

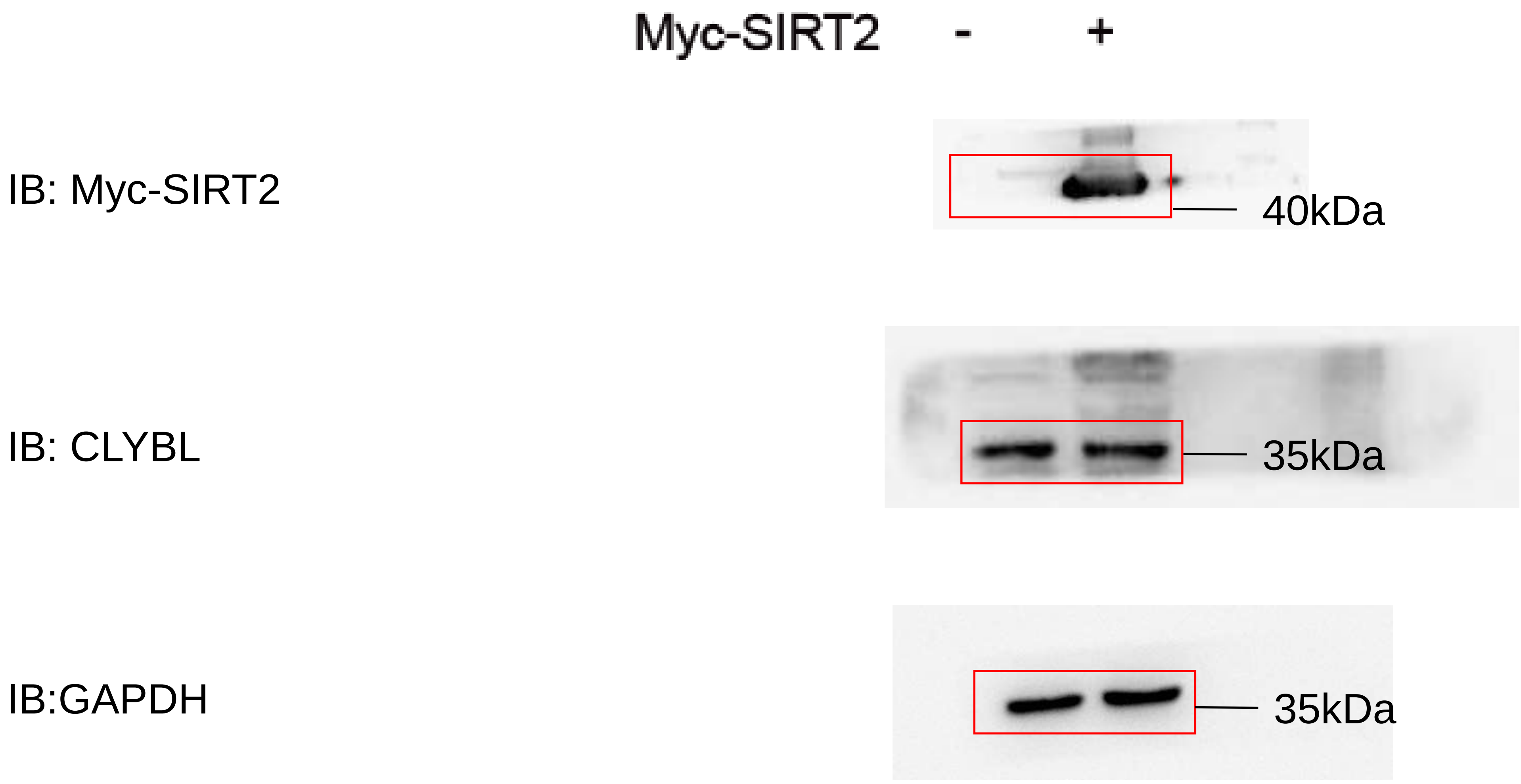

Uncropped Western blots for Figure 2 C

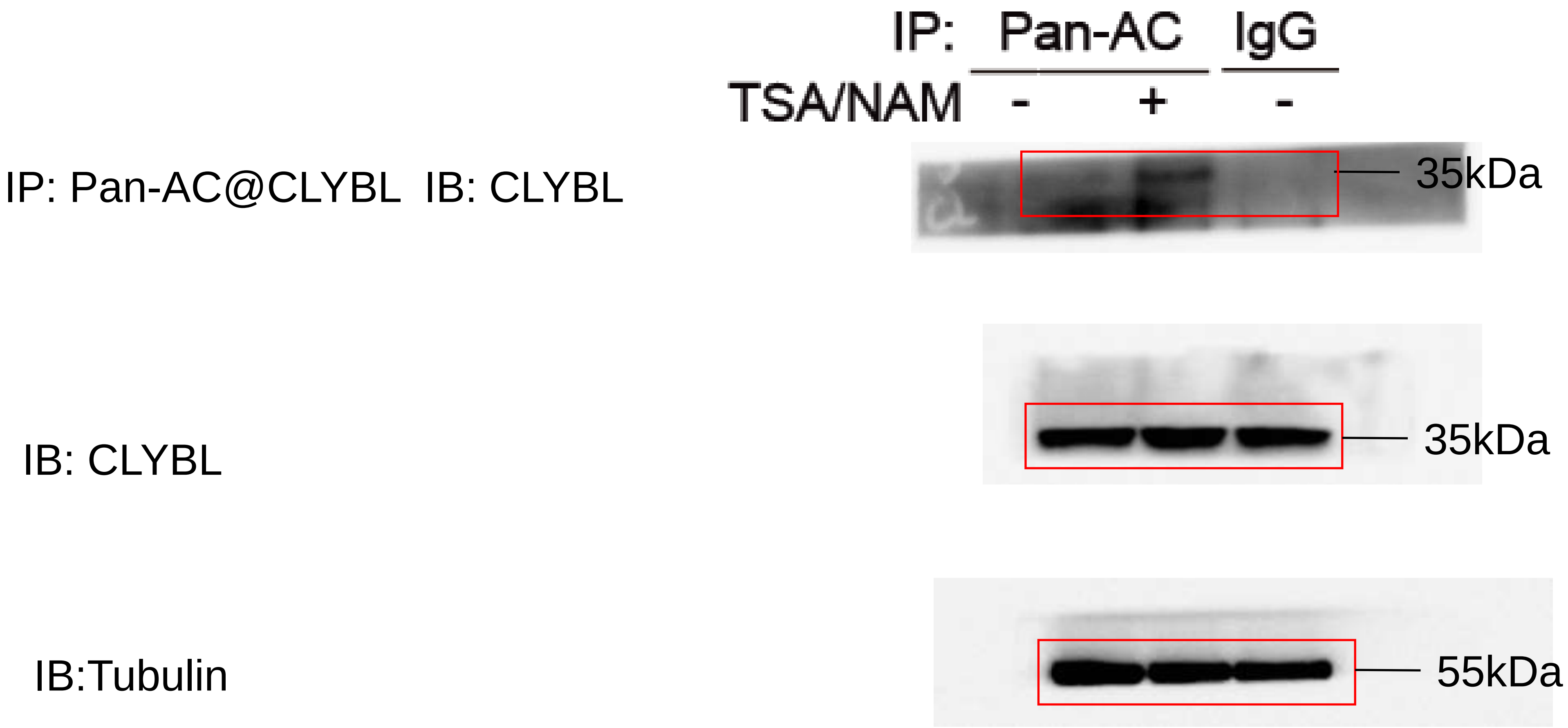

Uncropped Western blots for Figure 2 D

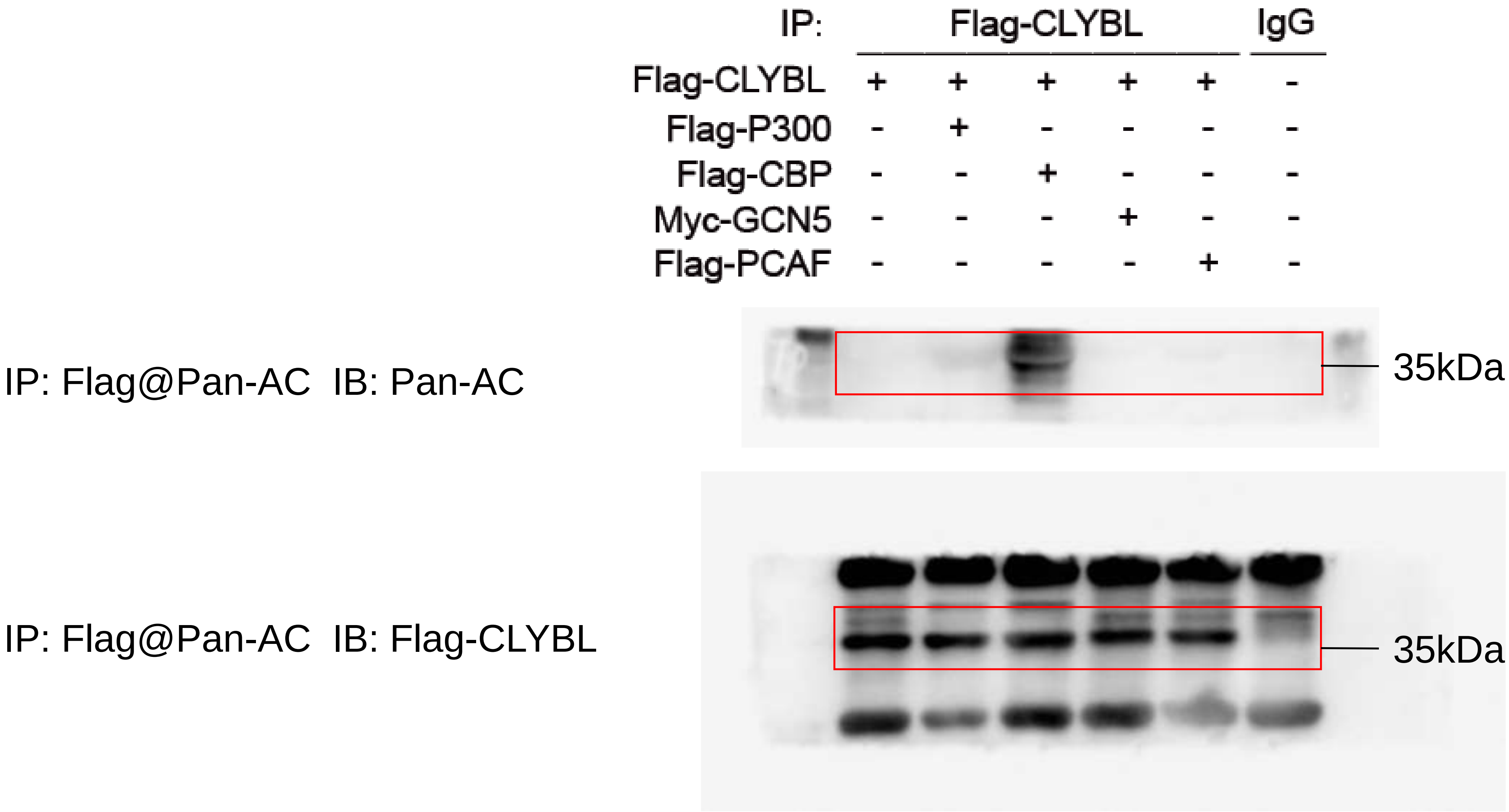

Uncropped Western blots for Figure 2 D

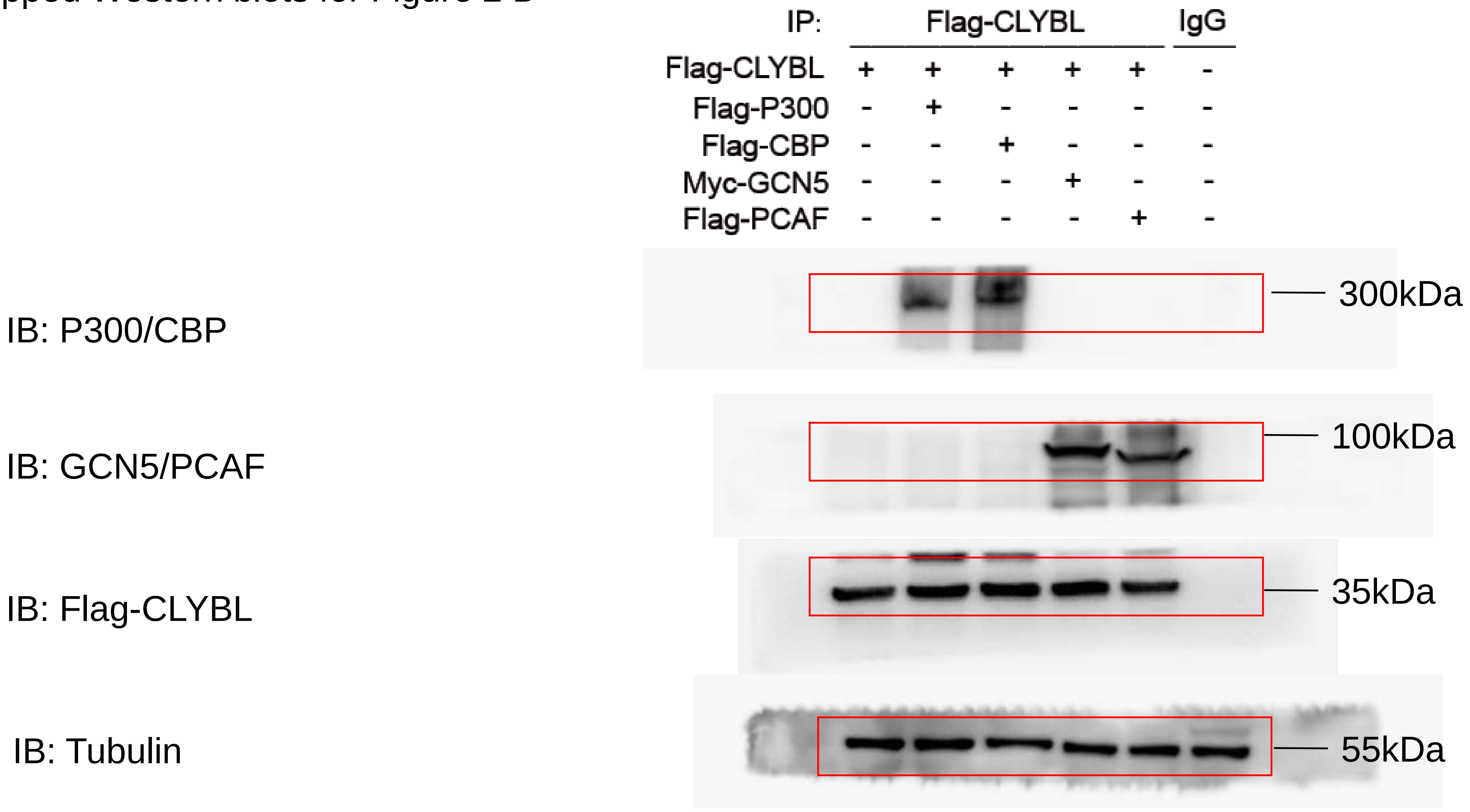

Uncropped Western blots for Figure 2 E

Flag-CBP - +

IP: Flag@CLYBL IB: CLYBL

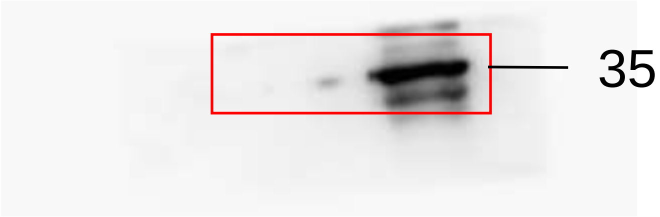

35kDa

IP: Flag@CLYBL IB: Flag-CBP

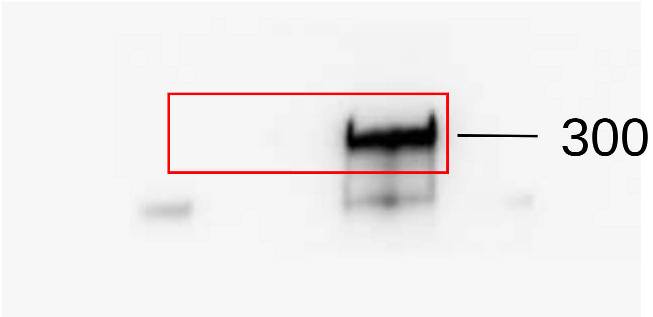

300kDa

Uncropped Western blots for Figure 2 E

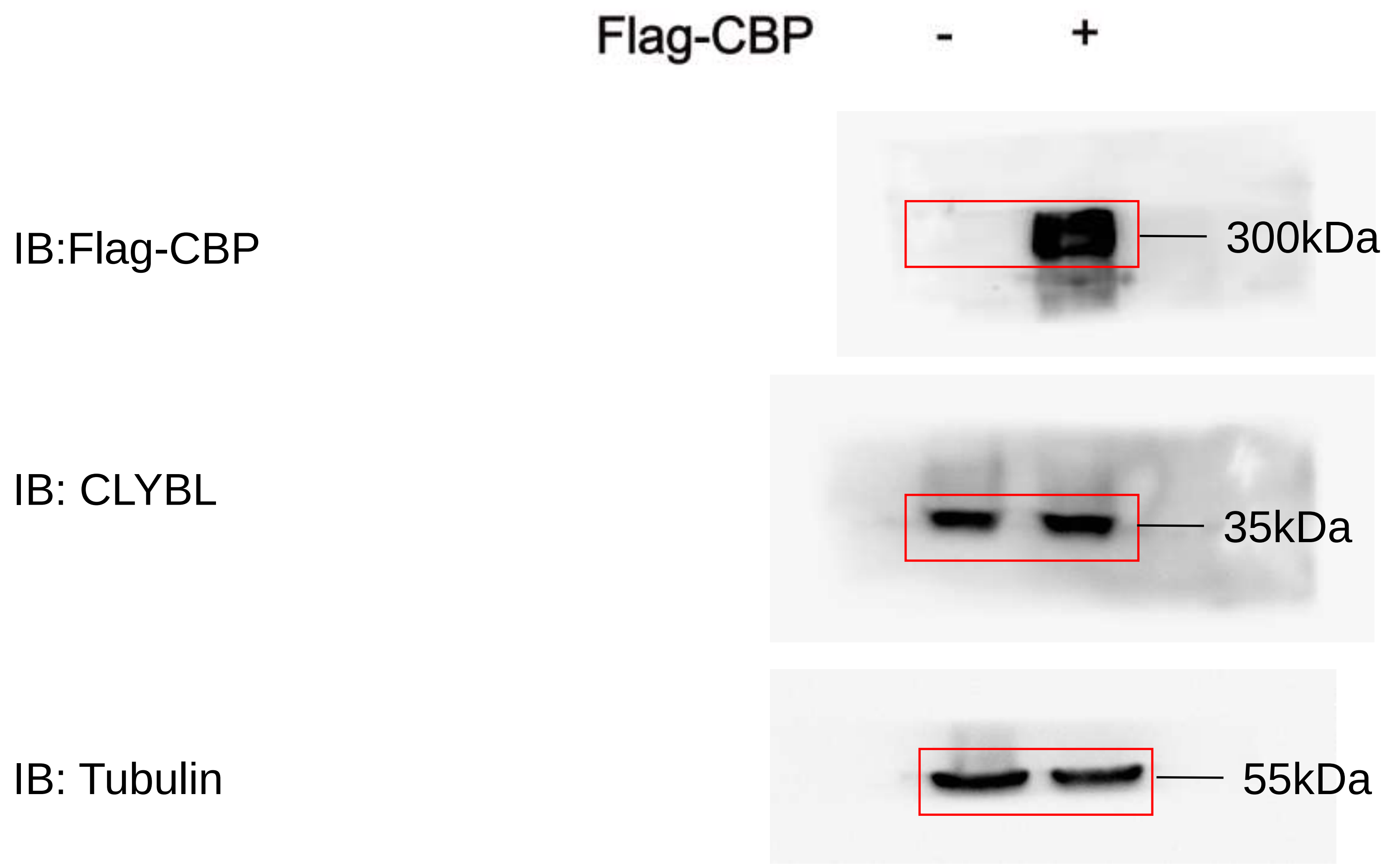

Uncropped Western blots for Figure 2 F

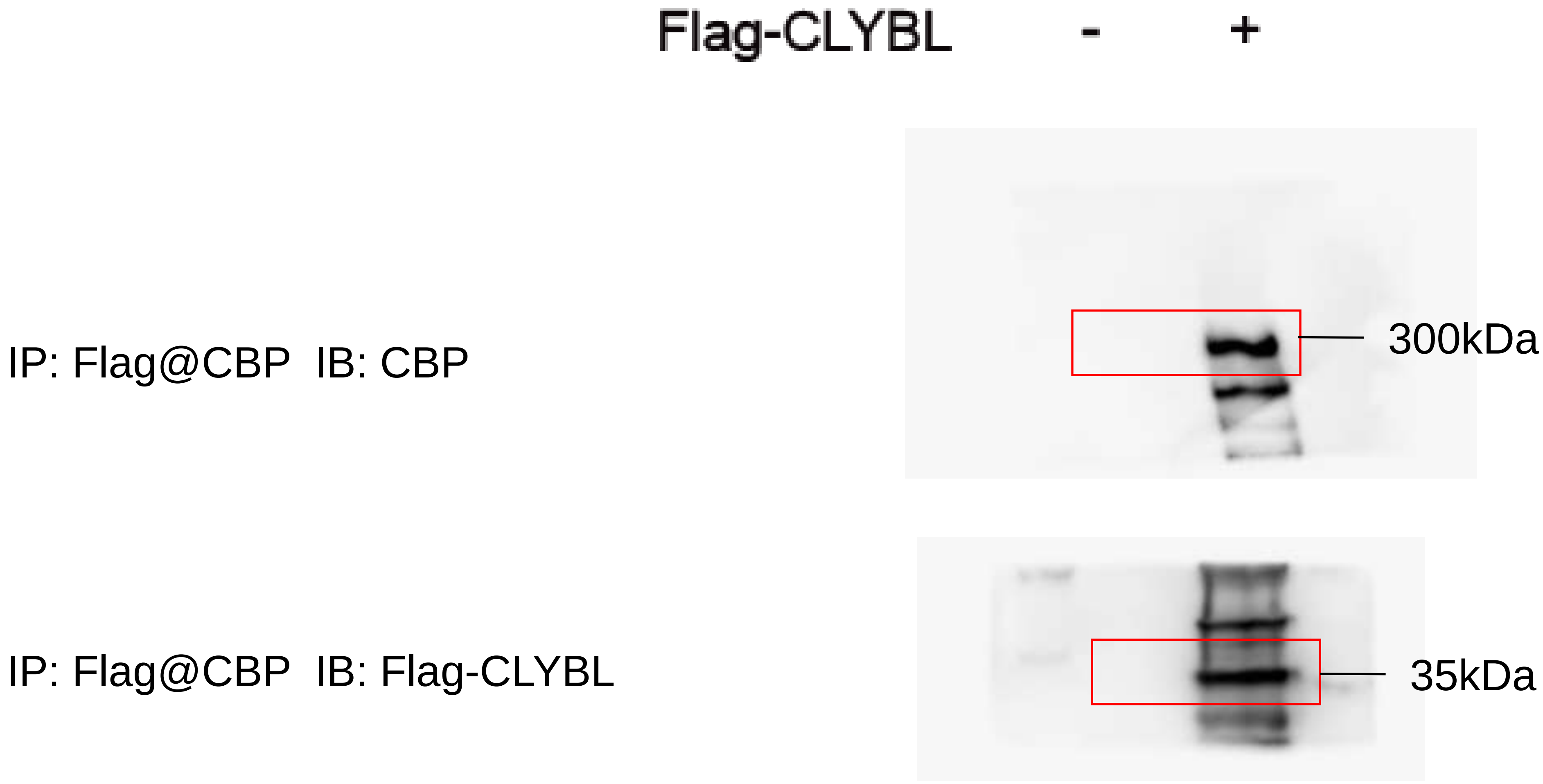

Uncropped Western blots for Figure 2 F

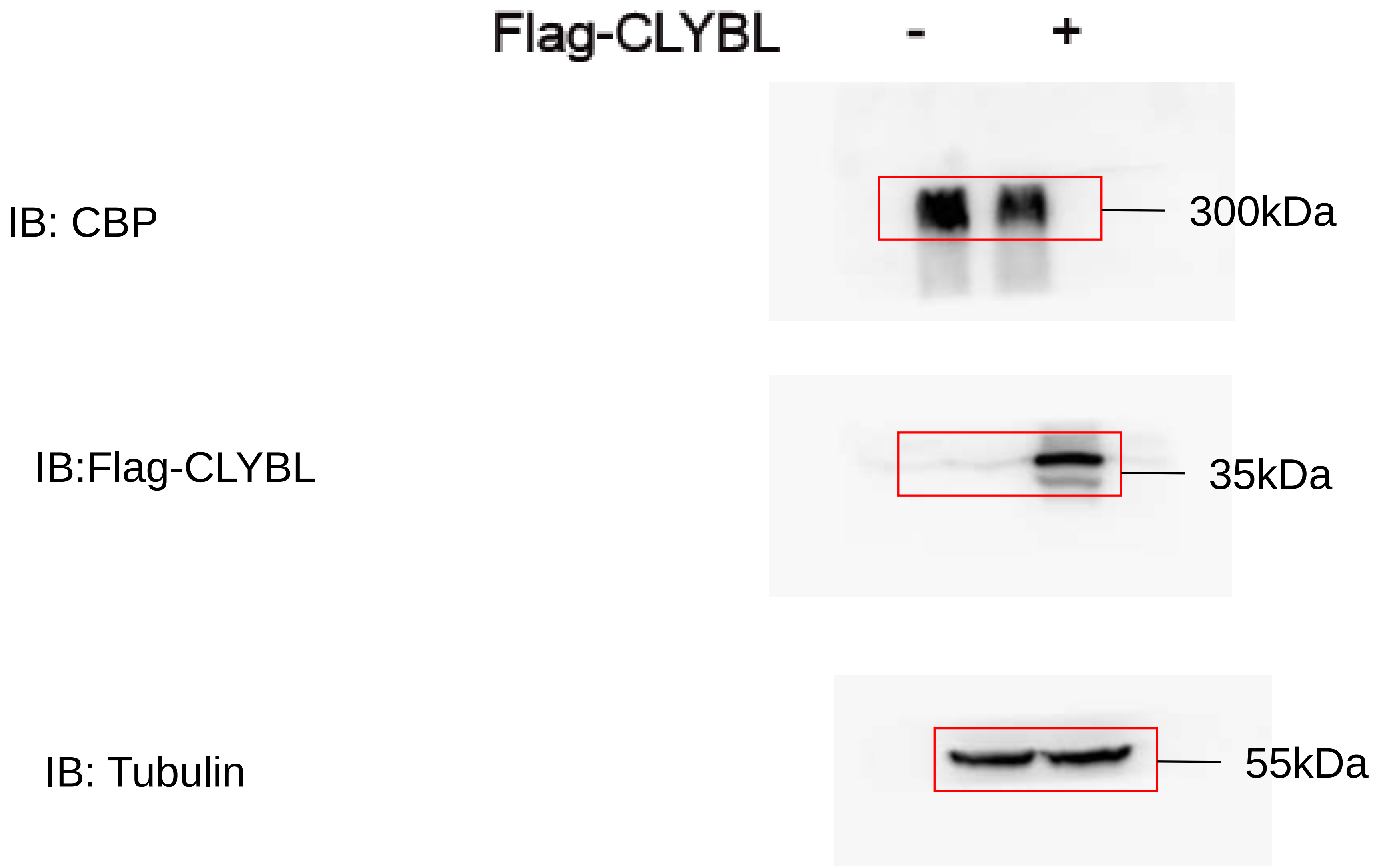

Uncropped Western blots for Figure 2 G

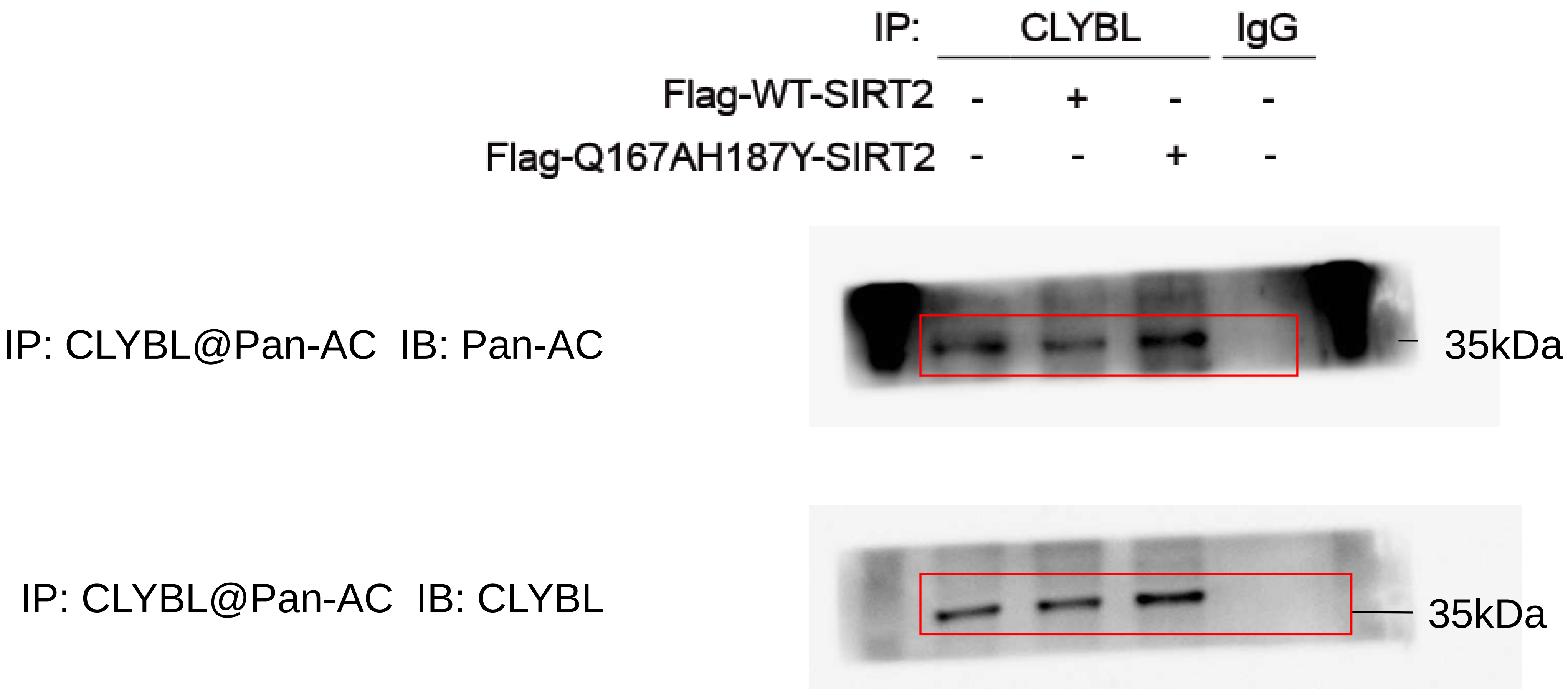

Uncropped Western blots for Figure 2 G

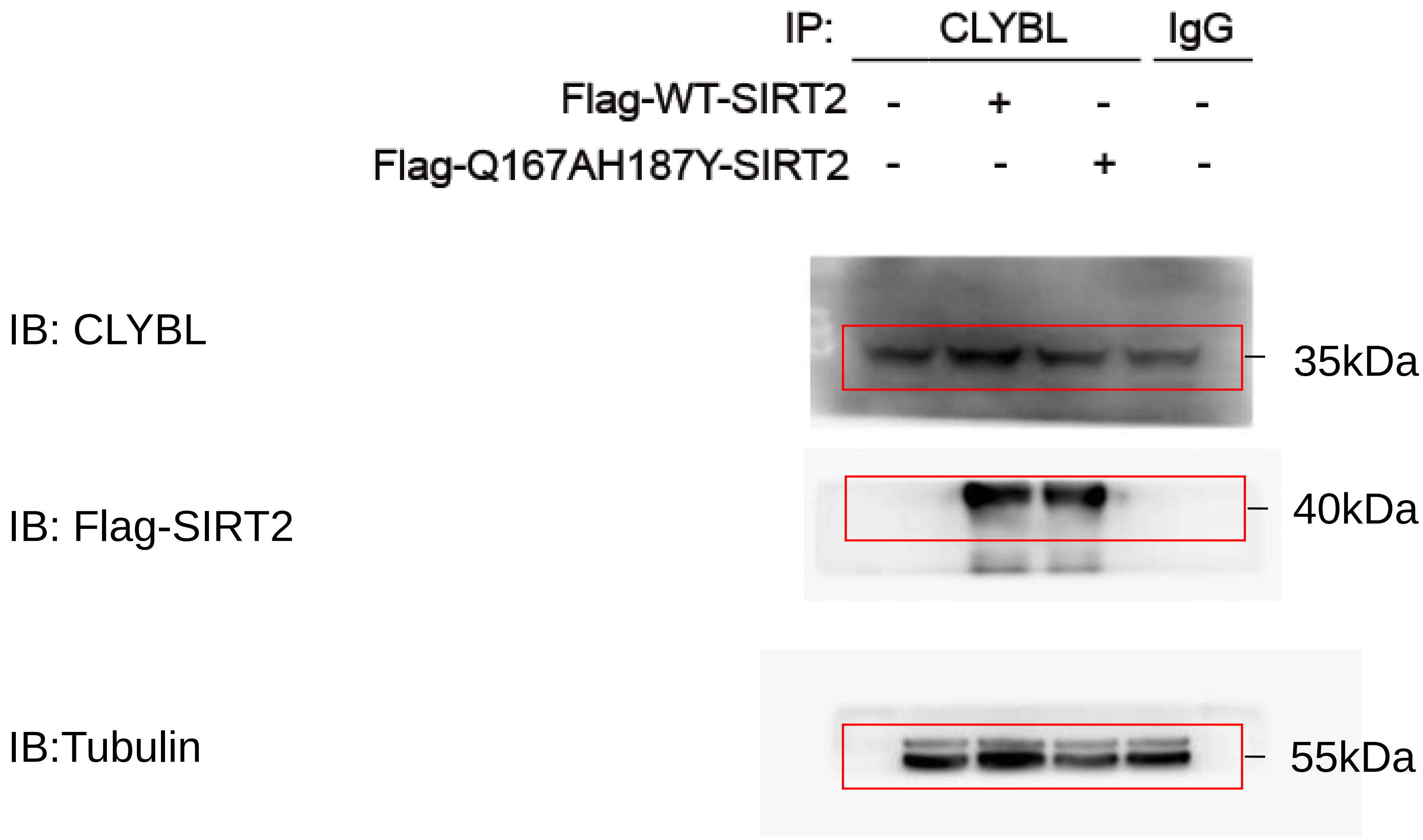

Uncropped Western blots for Figure 2 H

IP: Pan-AC@CLYBL IB: CLYBL

IB: CLYBL

IB:Tubulin

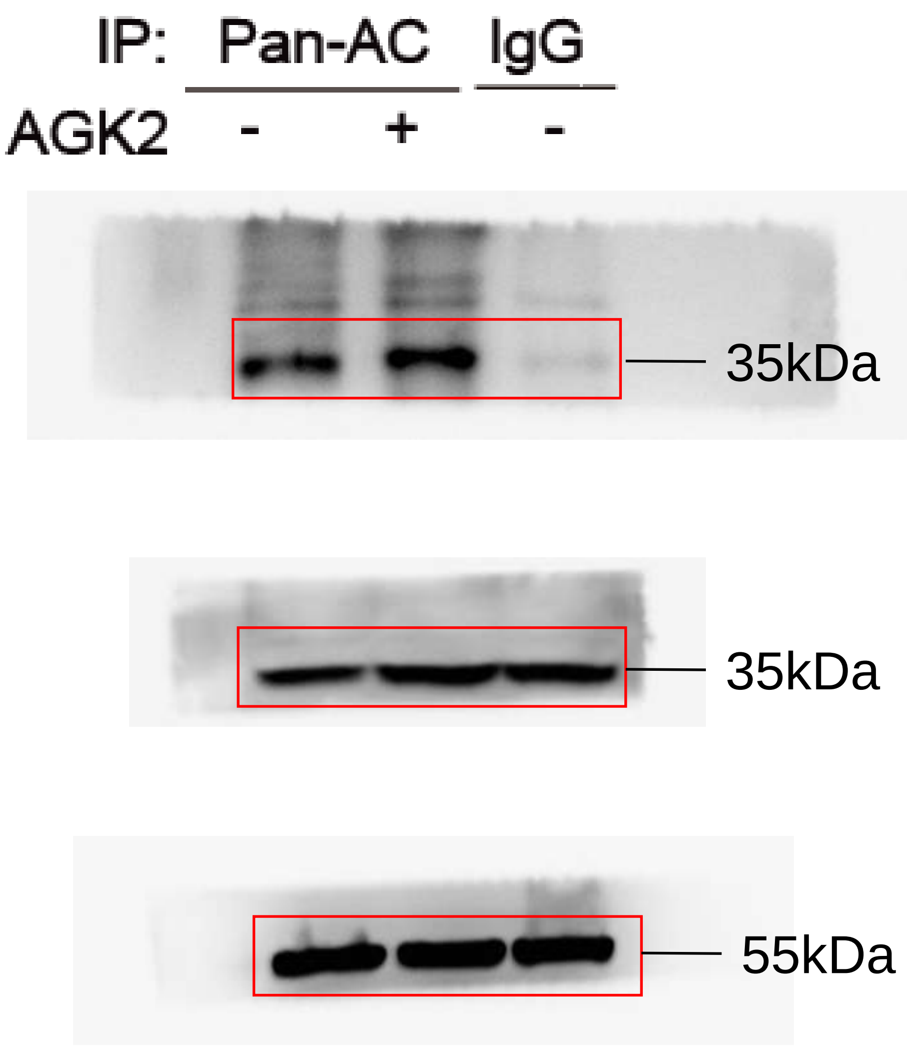

Uncropped Western blots for Figure 2 I

IP: Pan-AC@Flag IB: Flag-CLYBL

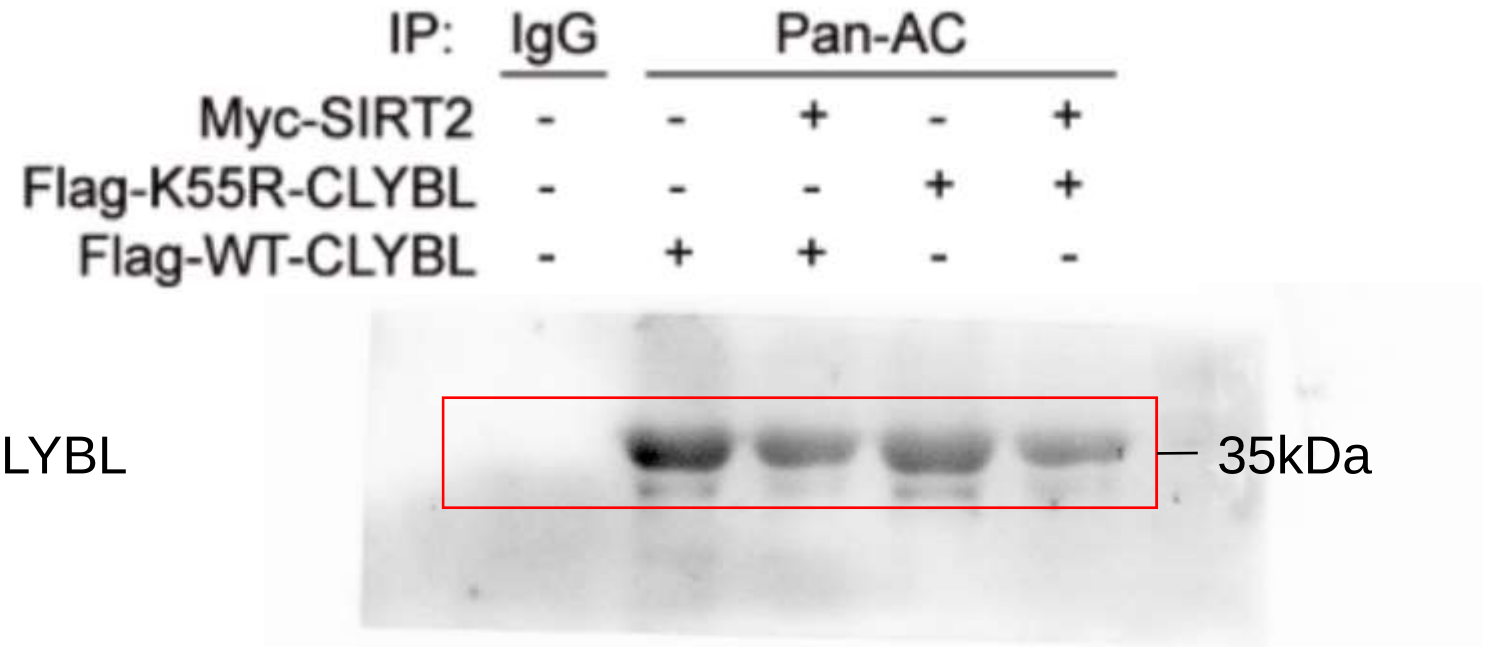

Uncropped Western blots for Figure 2 I

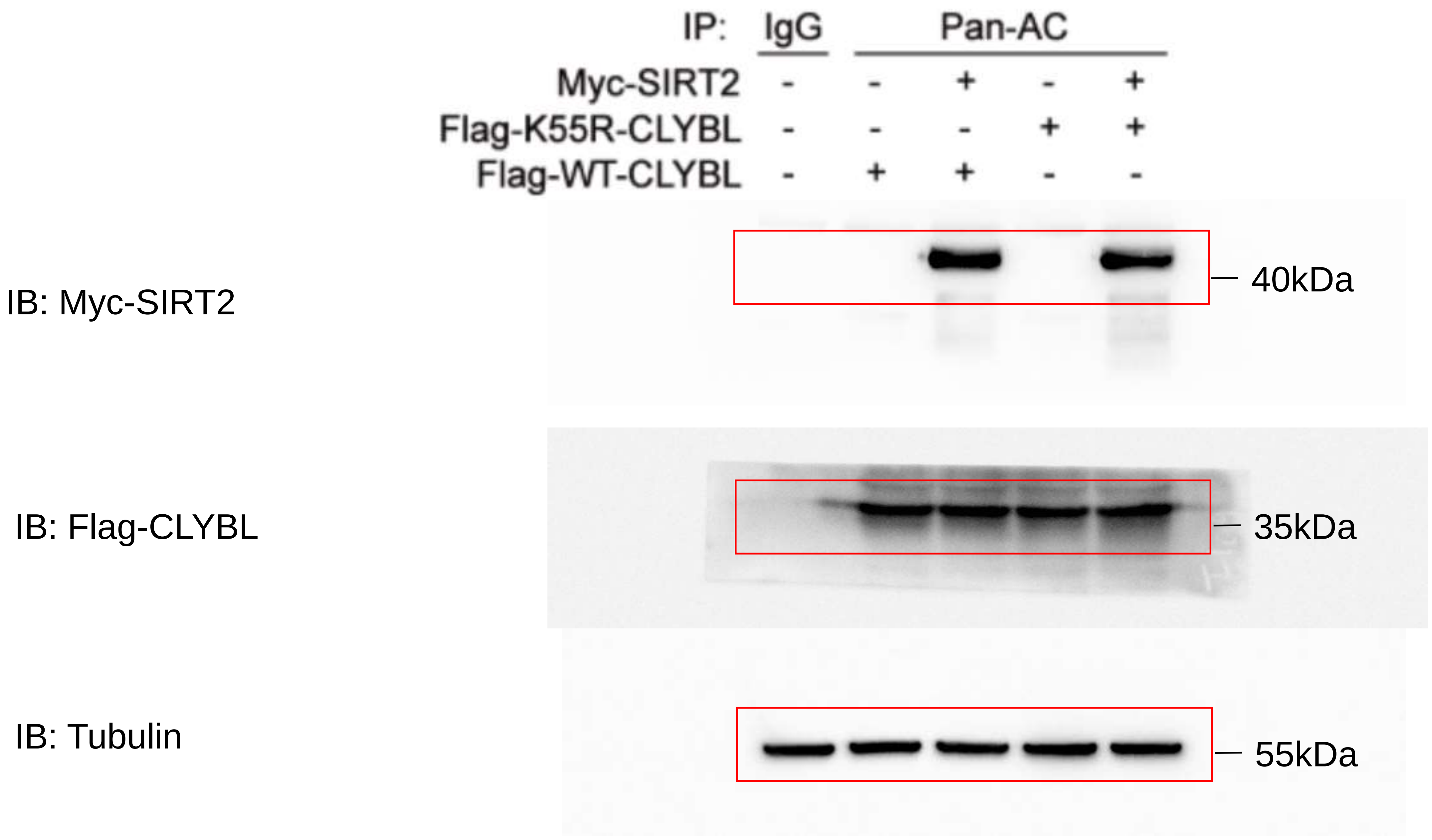

Uncropped Western blots for Figure 2 J

IP: Pan-AC@Flag IB: Flag-CLYBL

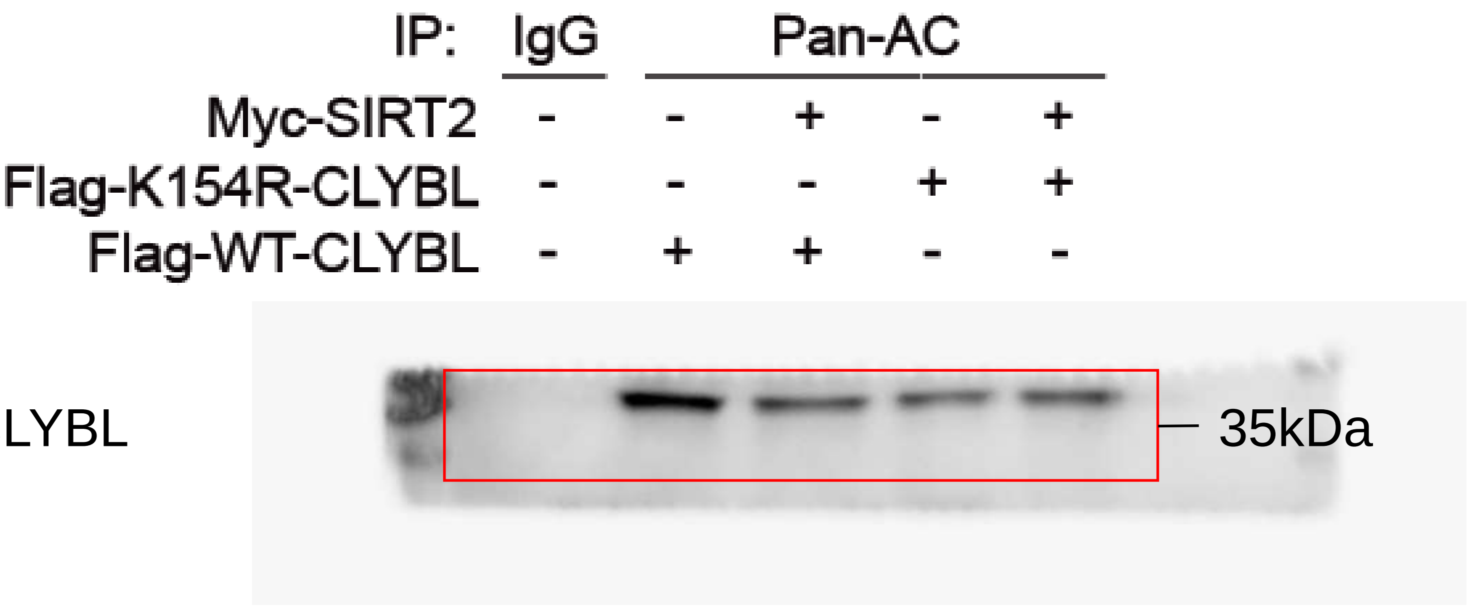

Uncropped Western blots for Figure 2 J

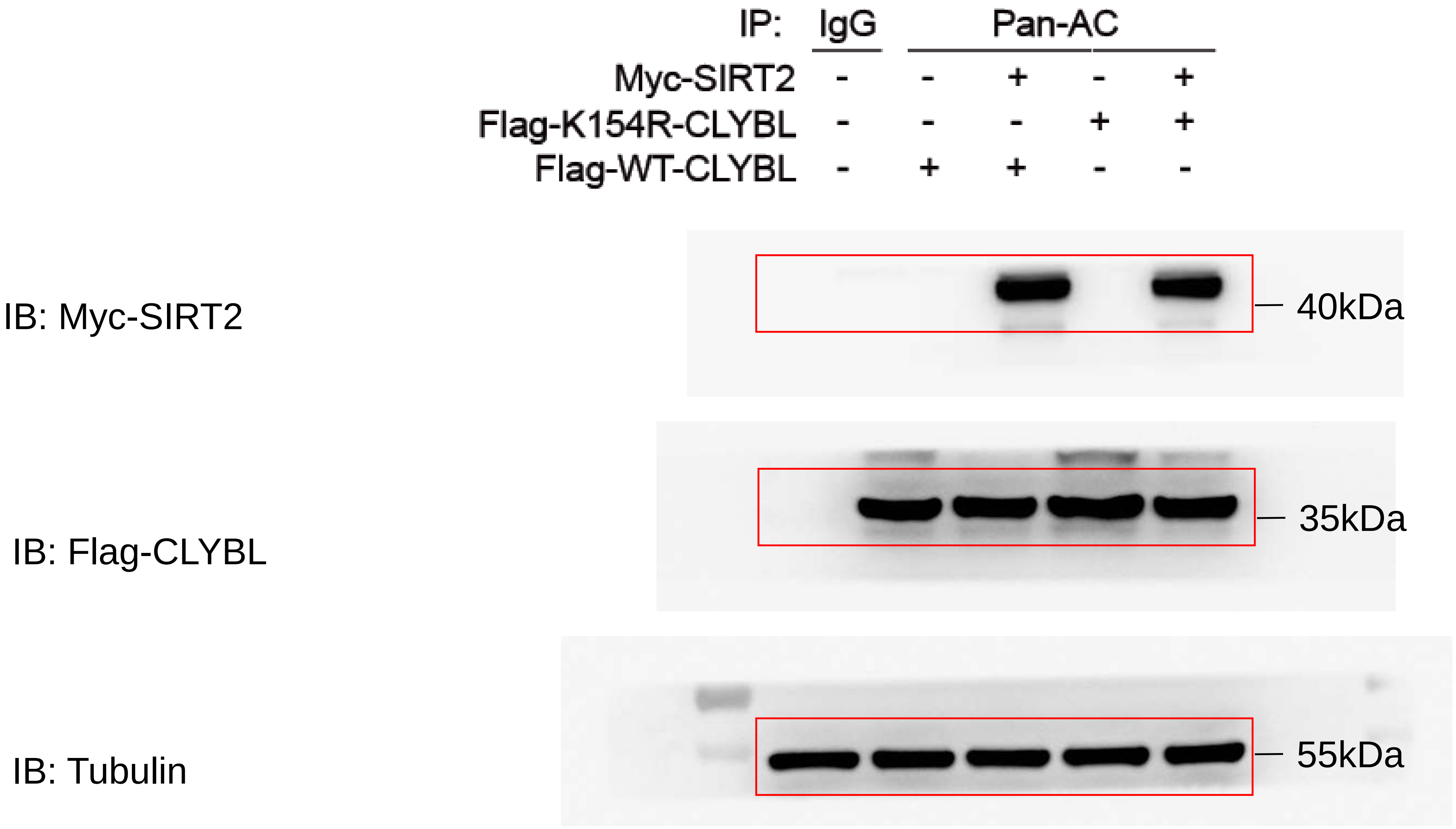

Uncropped Western blots for Figure 2 K

IP: Pan-AC@Flag IB: Flag-CLYBL

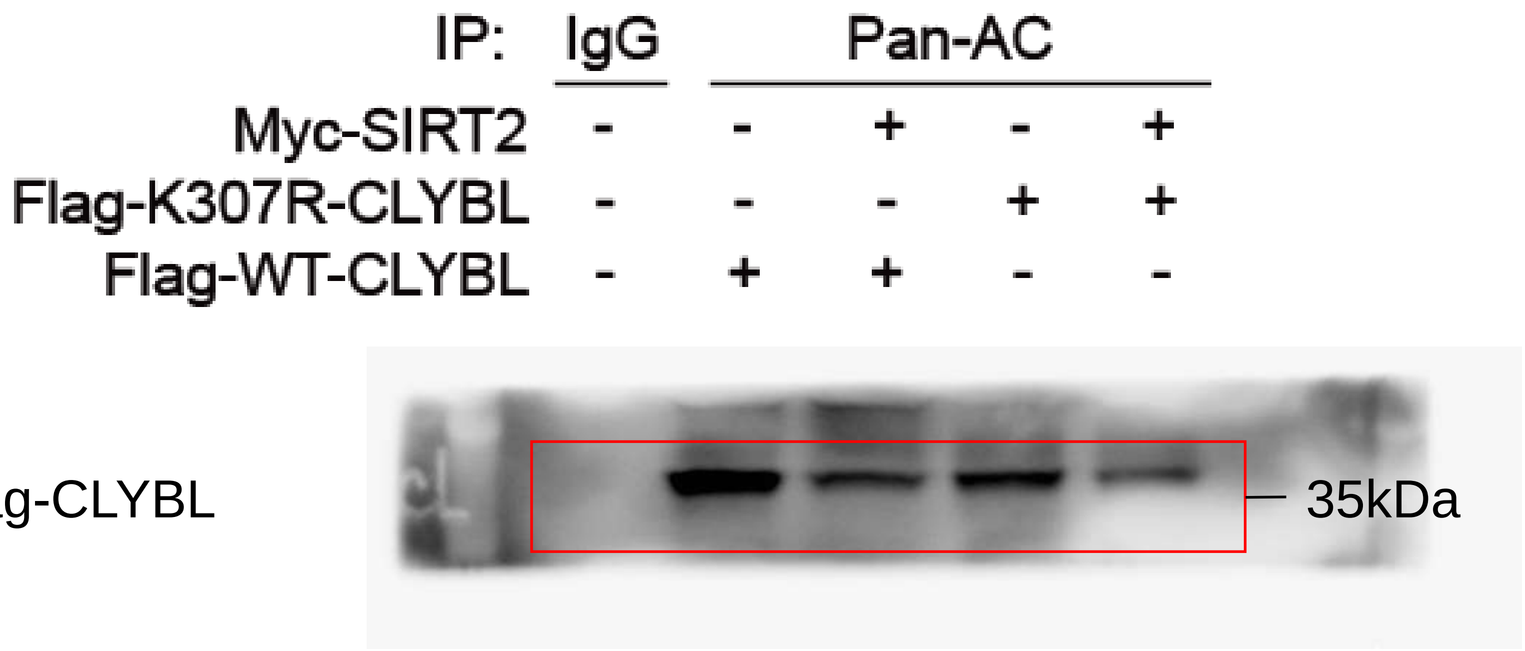

Uncropped Western blots for Figure 2 K

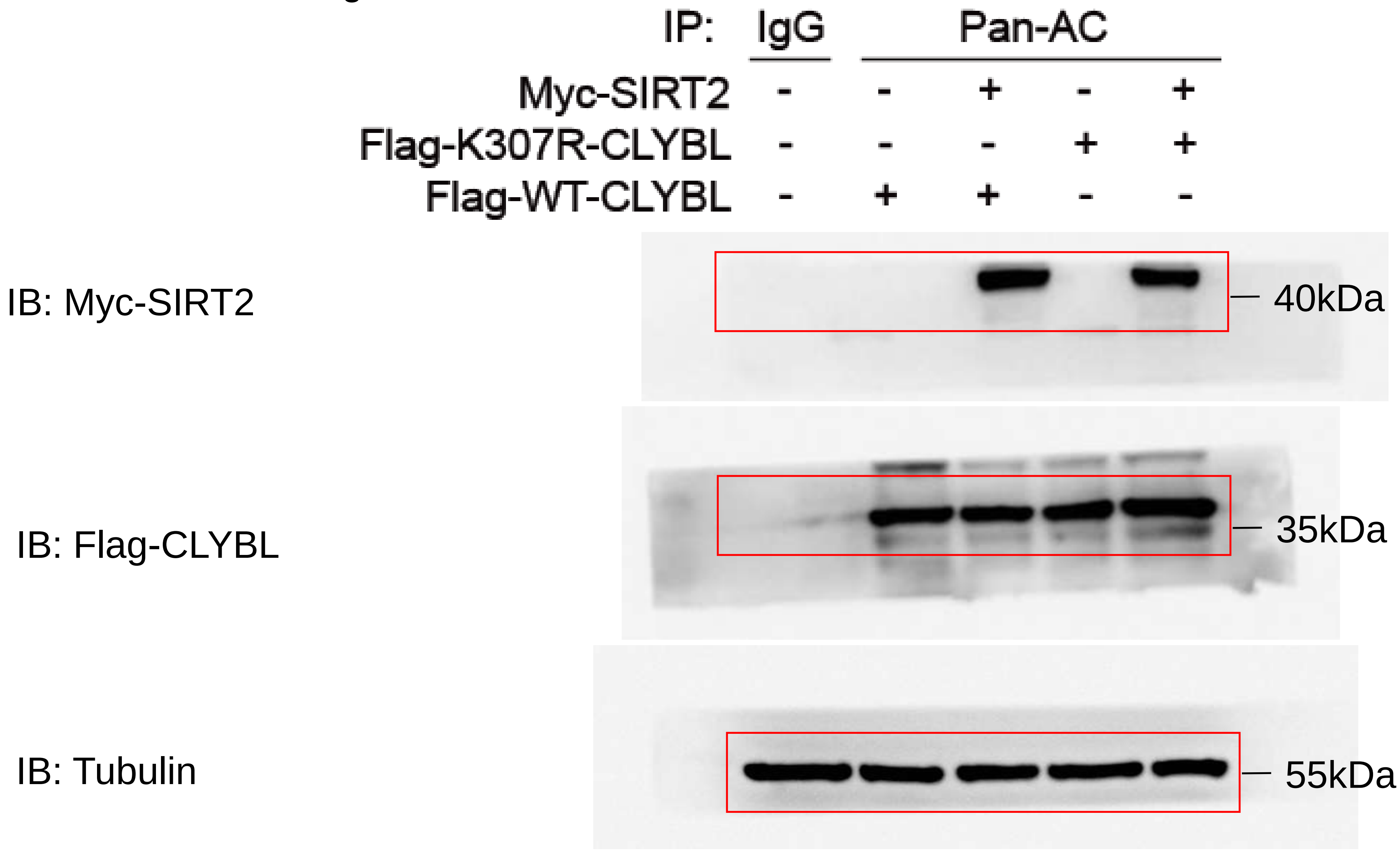

Uncropped Western blots for Figure 2 L

IP: Pan-AC@Flag IB: Flag-CLYBL

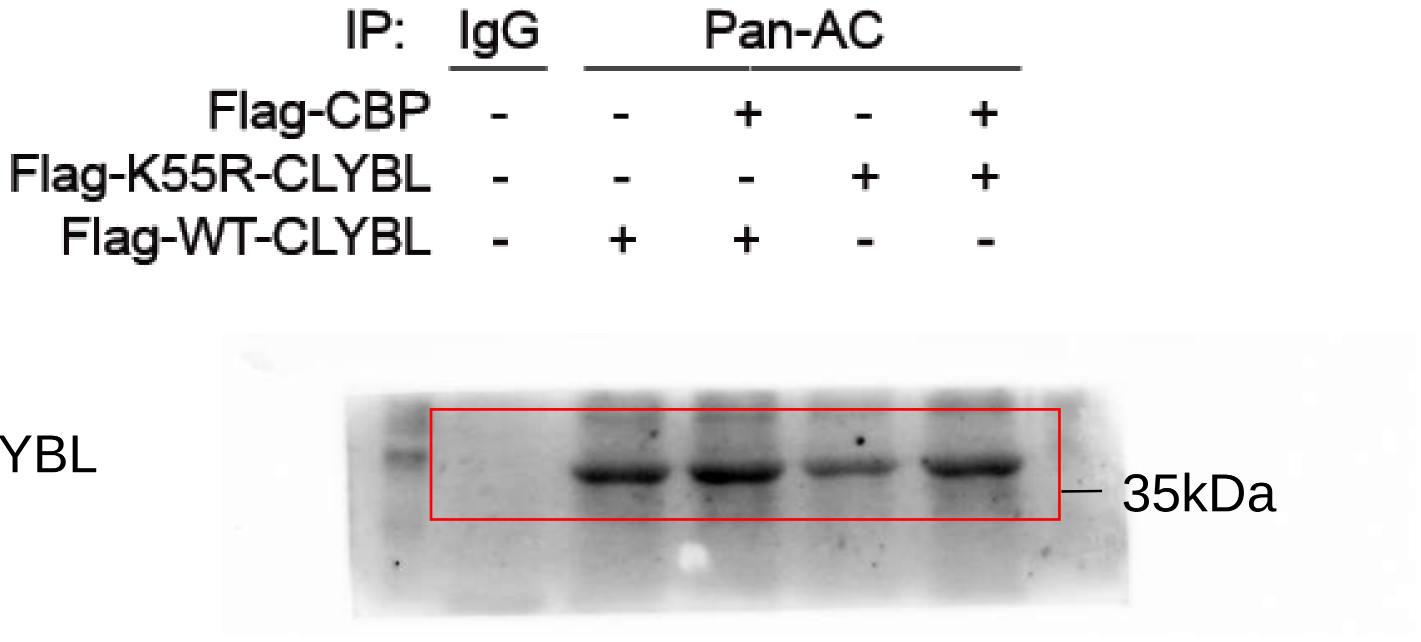

Uncropped Western blots for Figure 2 L

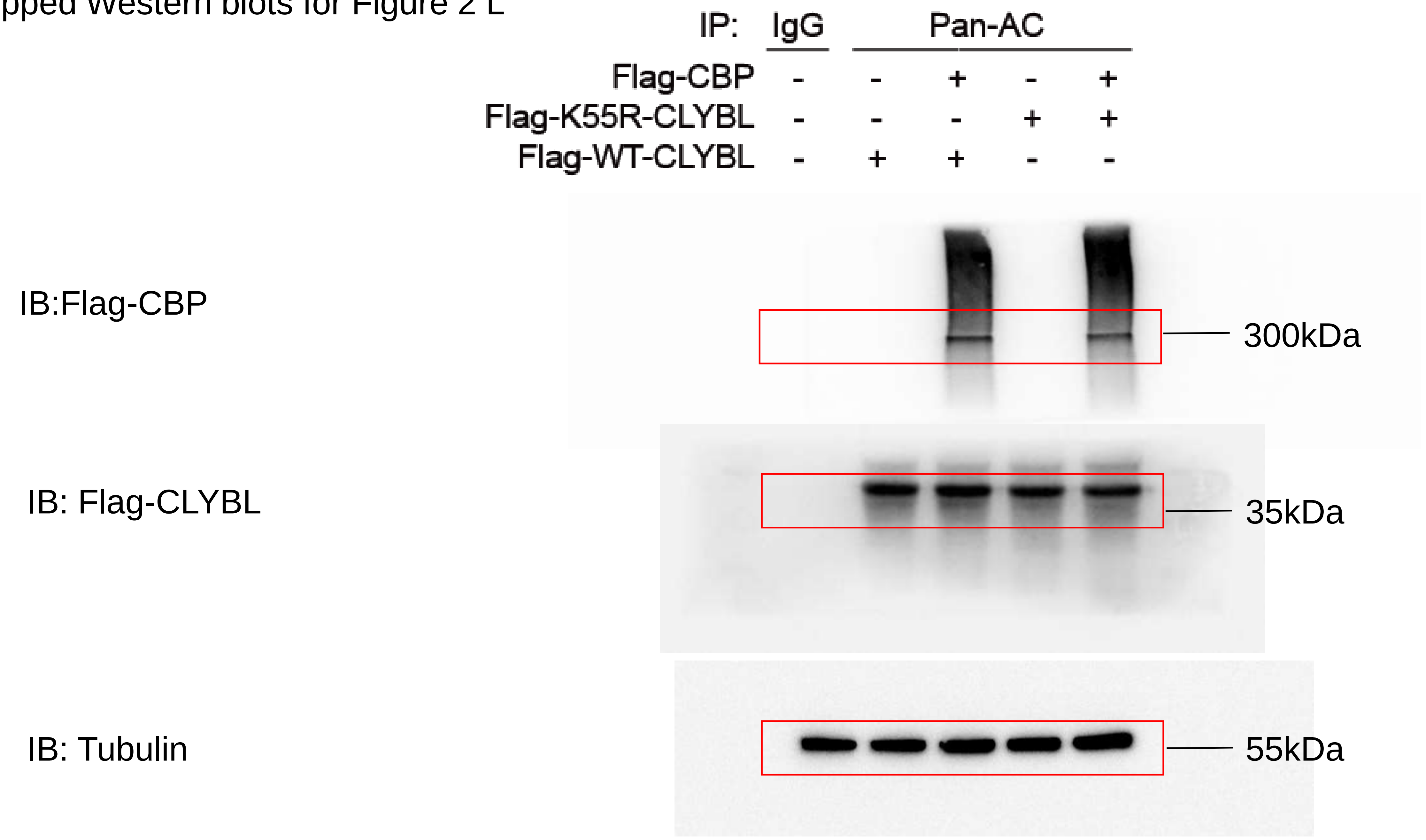

Uncropped Western blots for Figure 2 M

IP: Pan-AC@Flag IB: Flag-CLYBL

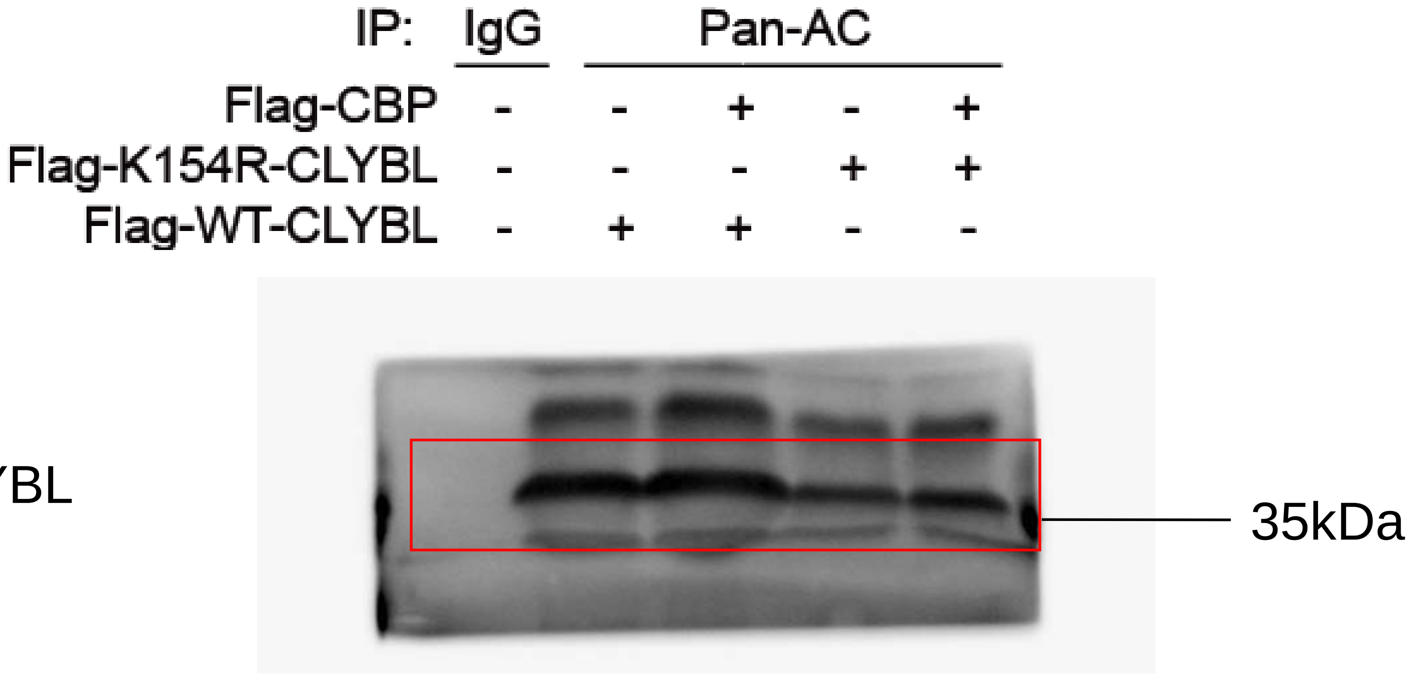

Uncropped Western blots for Figure 2 M

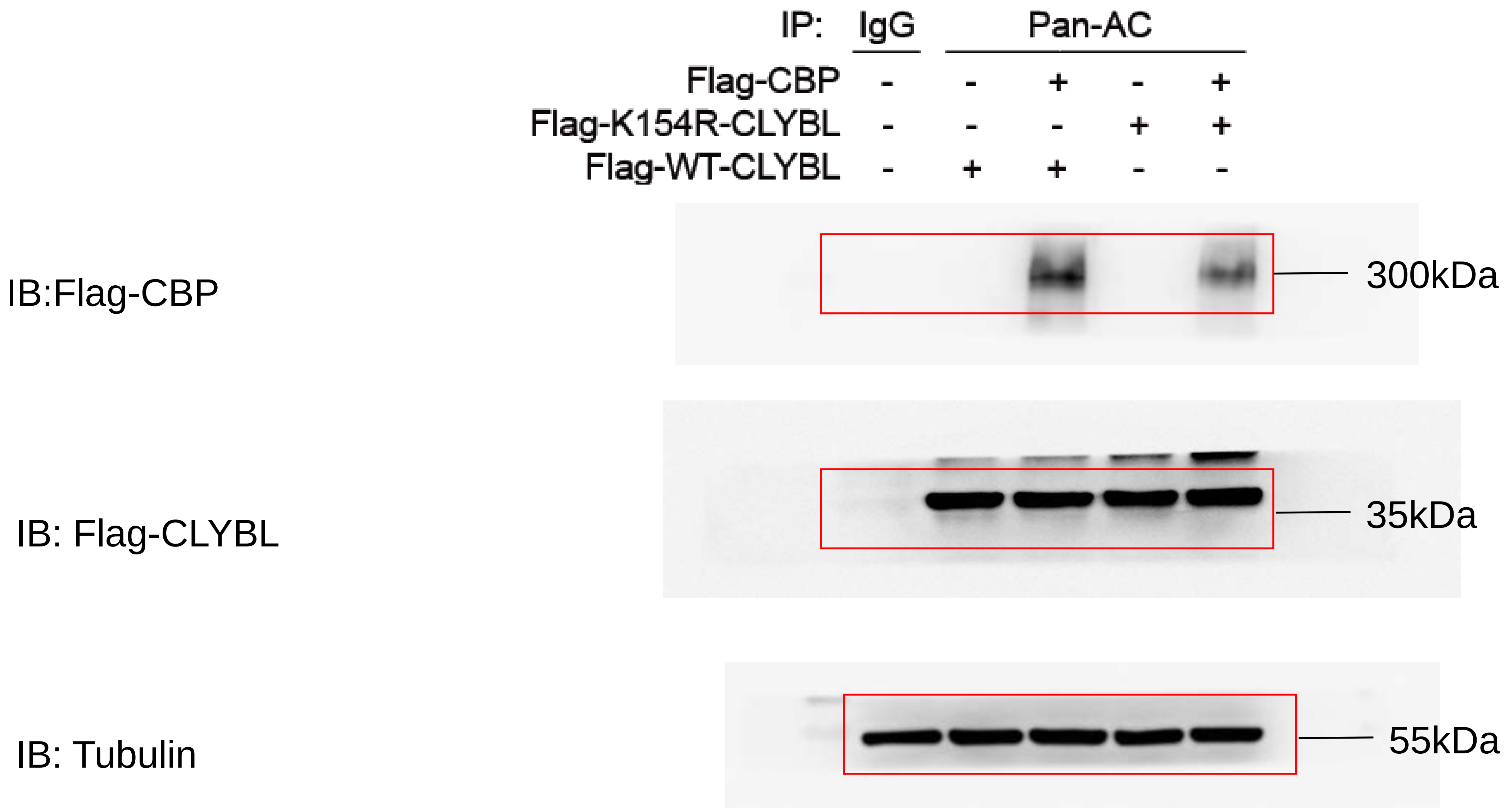

Uncropped Western blots for Figure 2 N

IP: Pan-AC@Flag IB: Flag-CLYBL

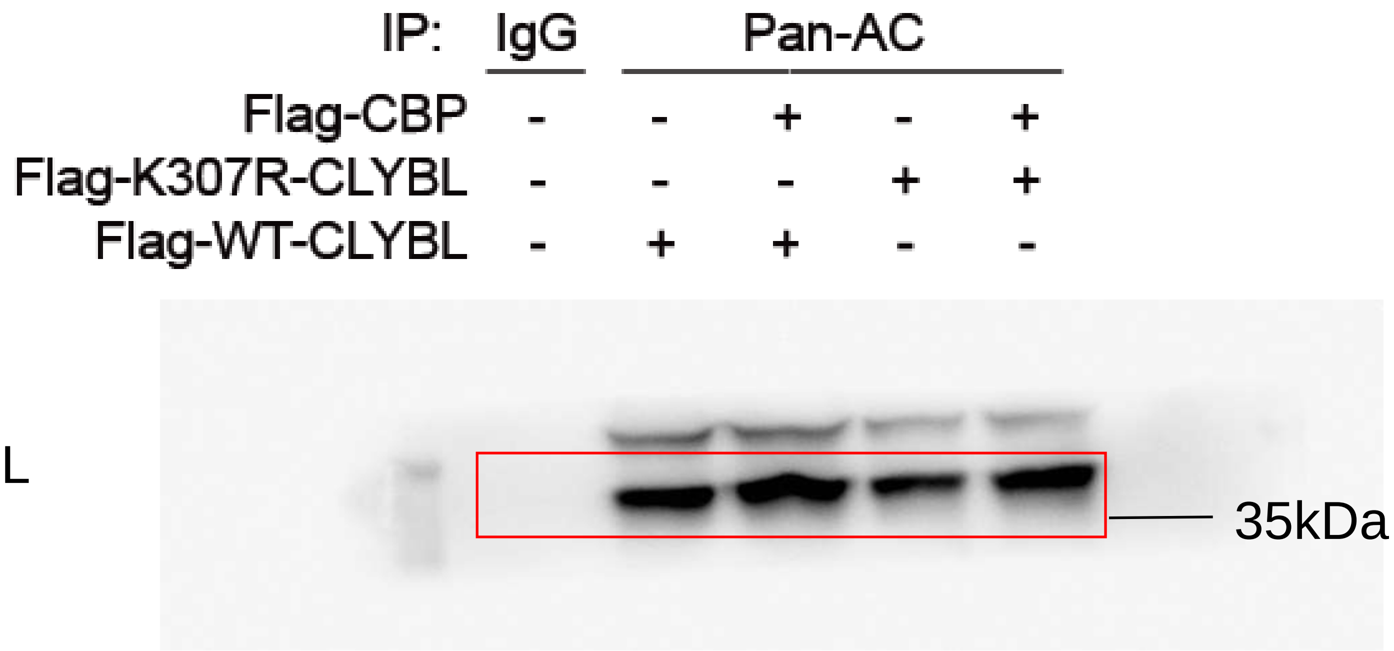

Uncropped Western blots for Figure 2 N

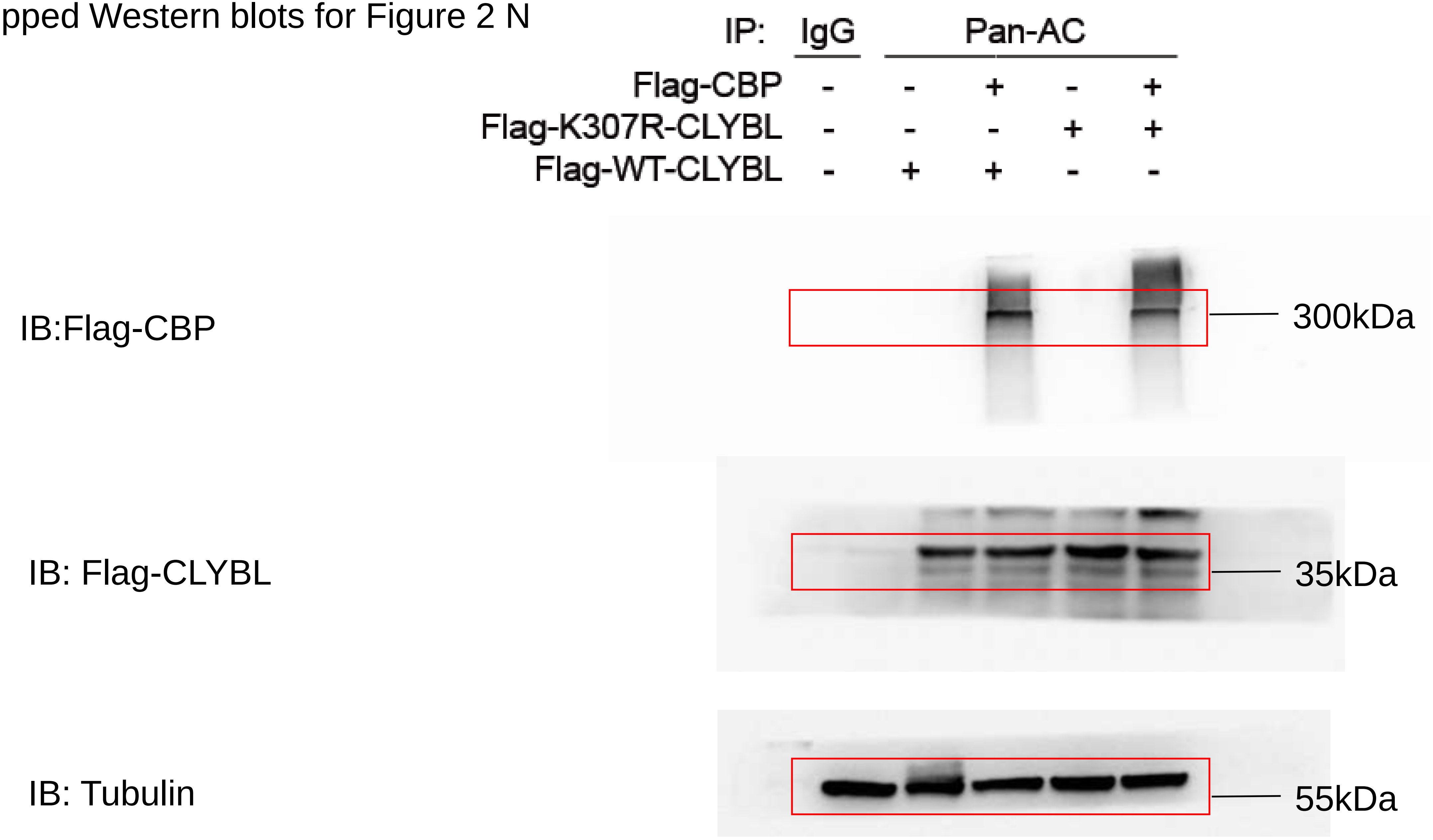

Uncropped Western blots for Figure 3

Uncropped Western blots for Figure 3 A

IP: CLYBL@Pan-AC IB: Pan-AC

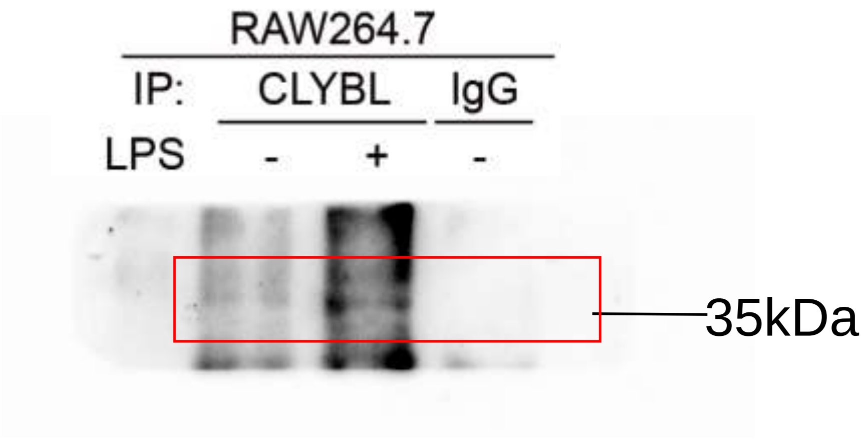

IP: CLYBL@Pan-AC IB: CLYBL

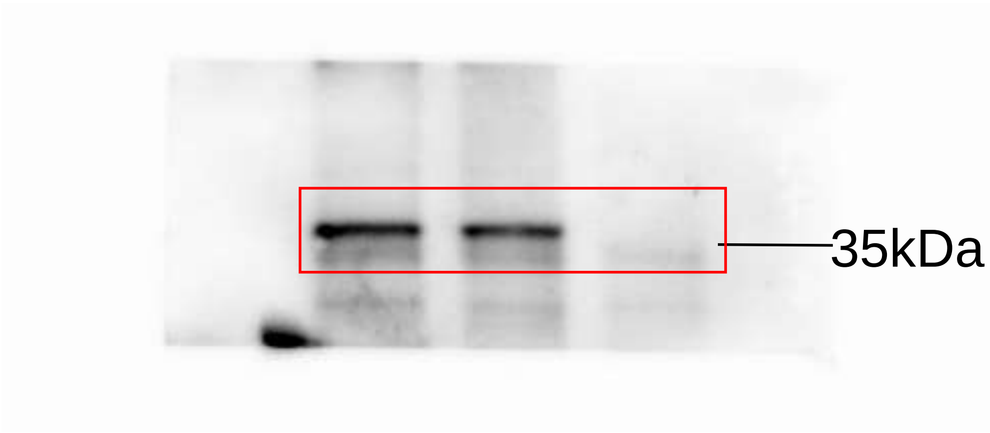

Uncropped Western blots for Figure 3 A

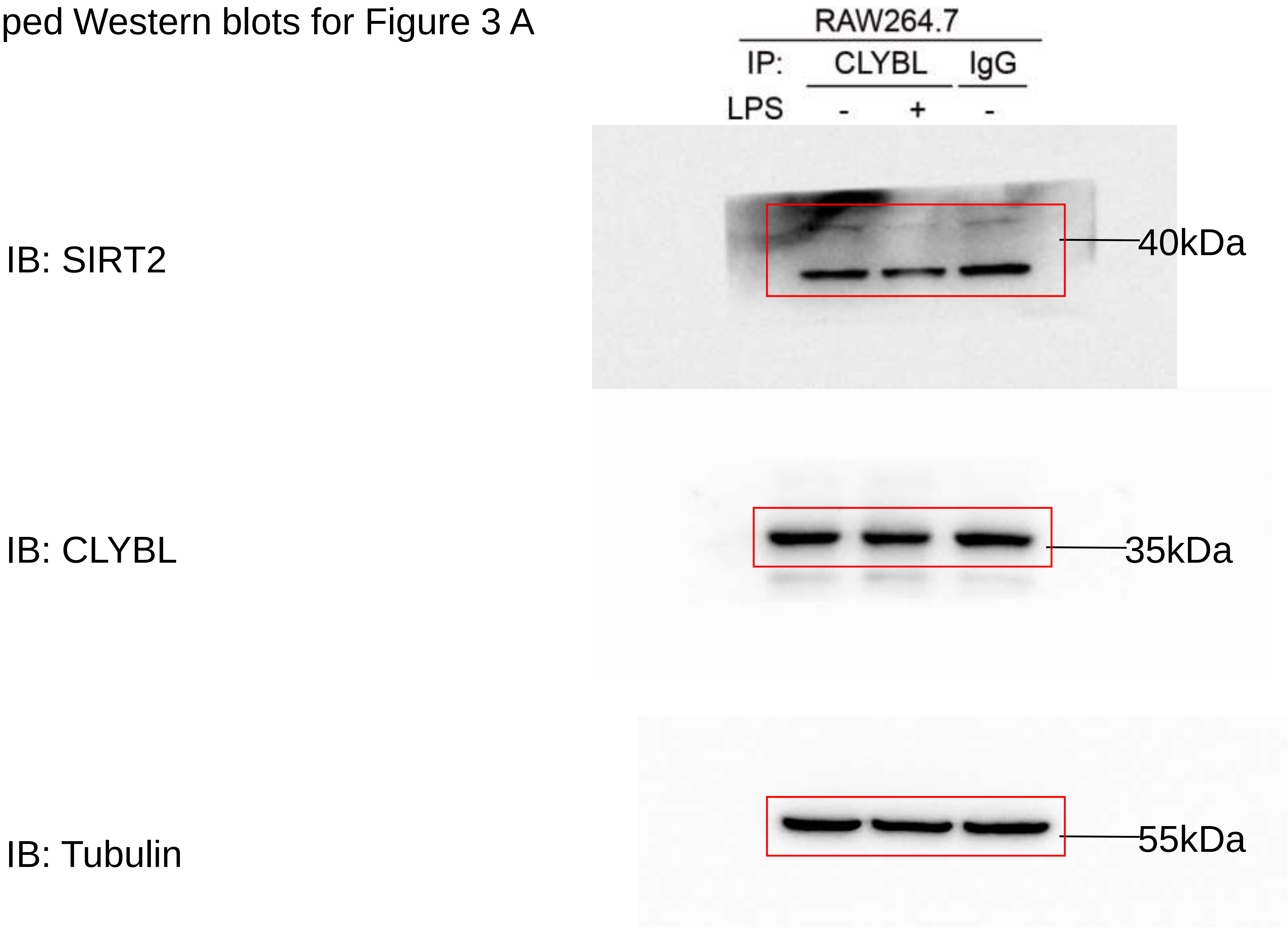

Uncropped Western blots for Figure 3 B

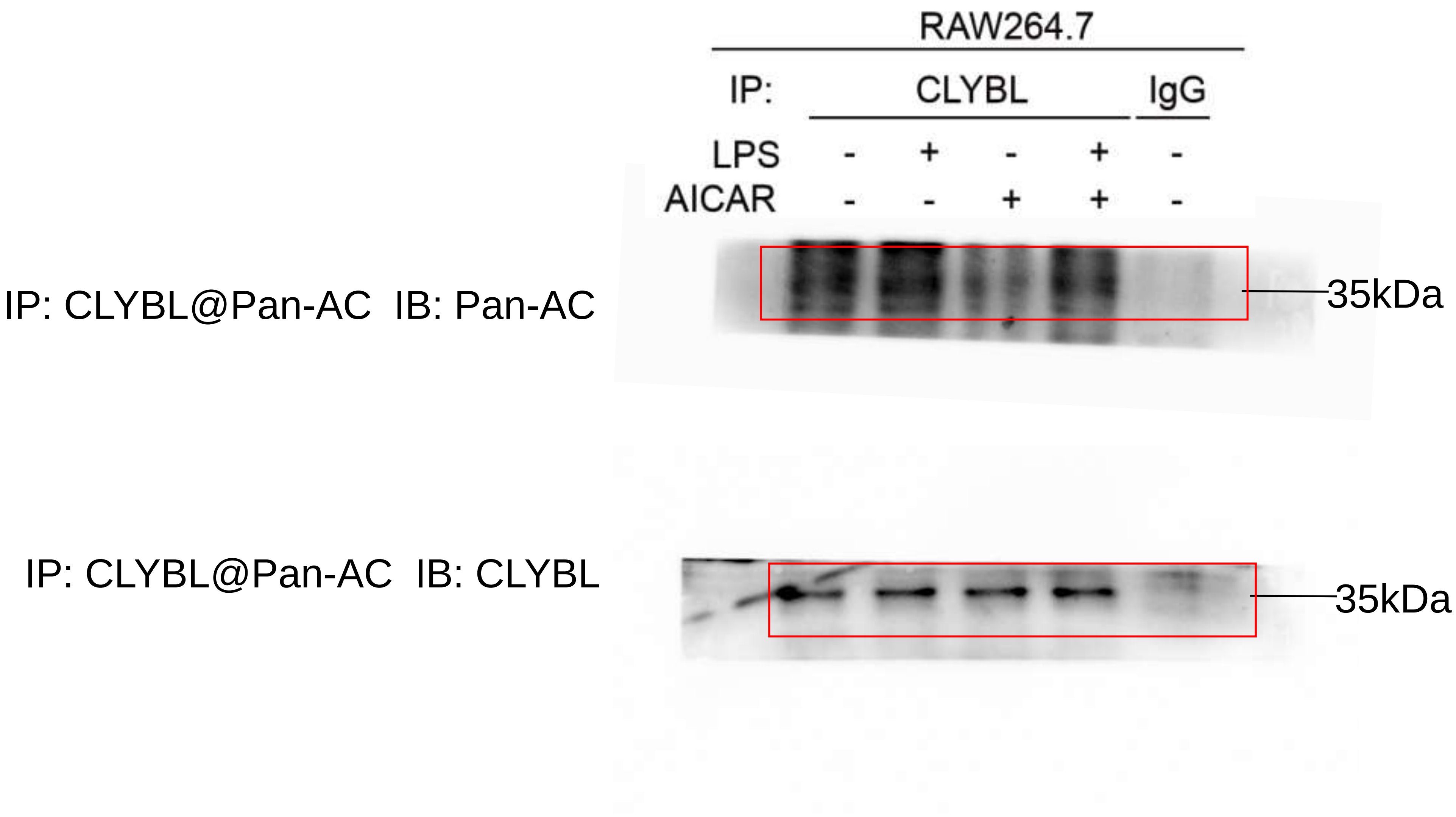

Uncropped Western blots for Figure 3 B

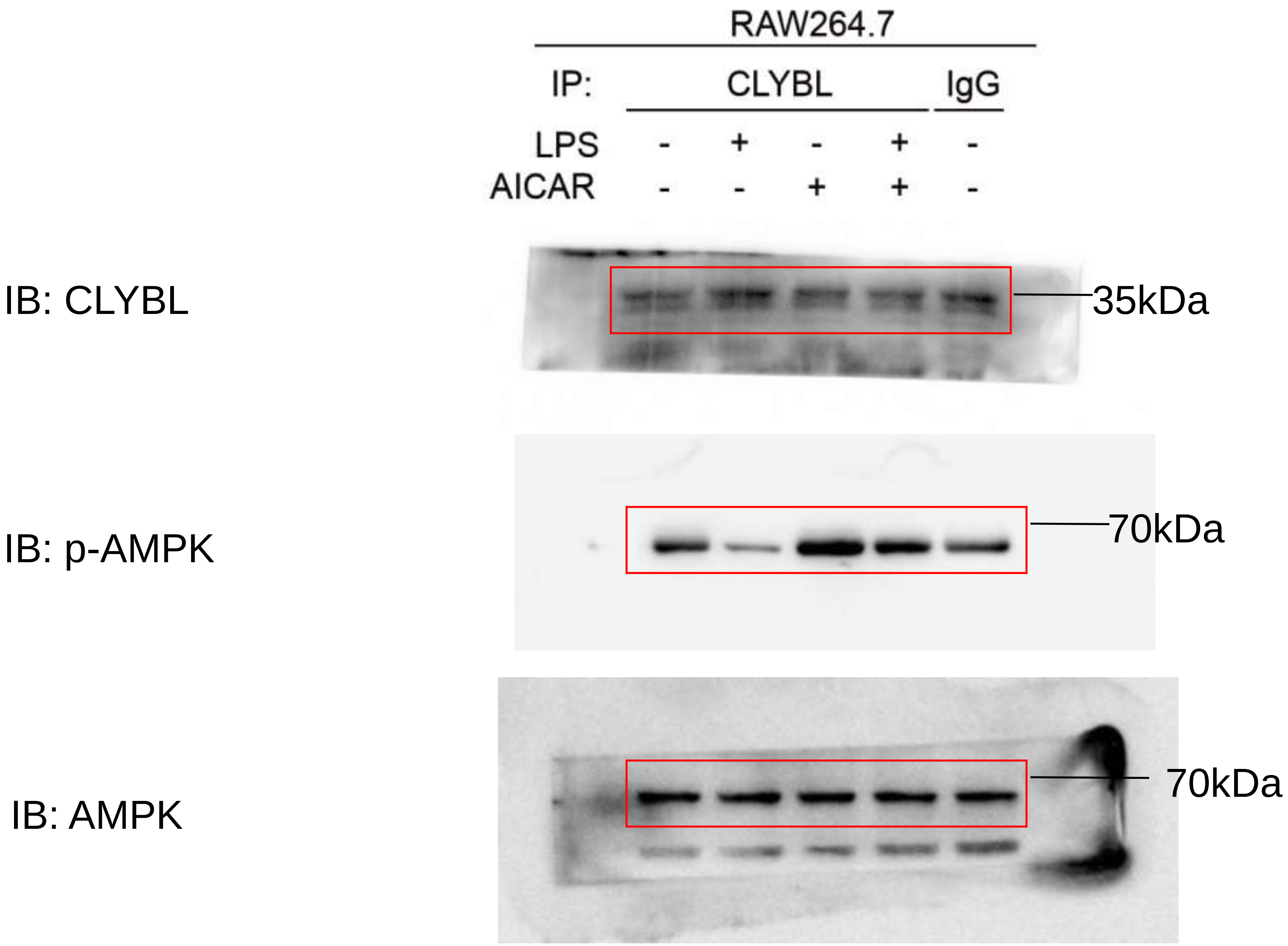

Uncropped Western blots for Figure 3 B

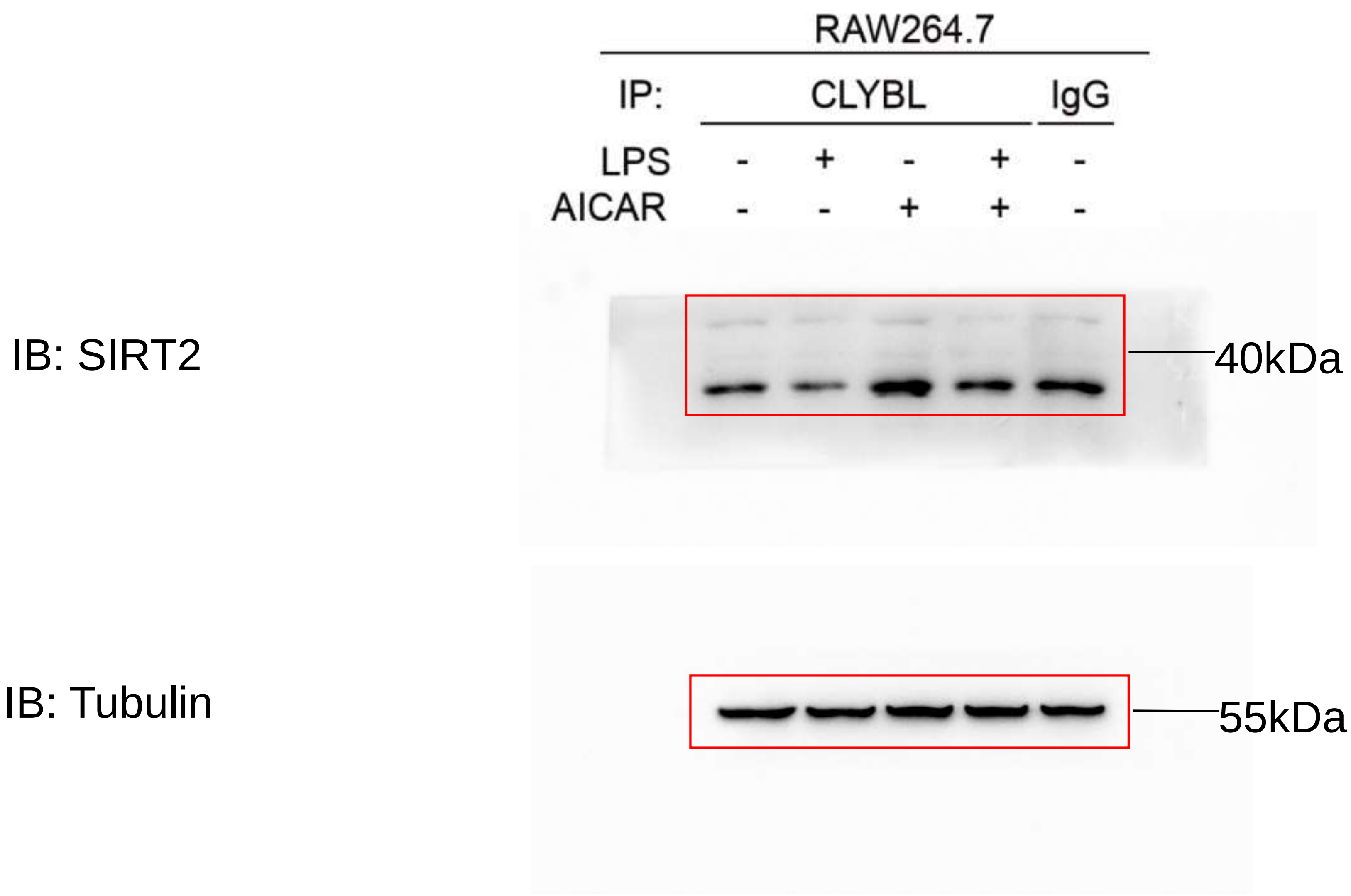

Uncropped Western blots for Figure 3 C

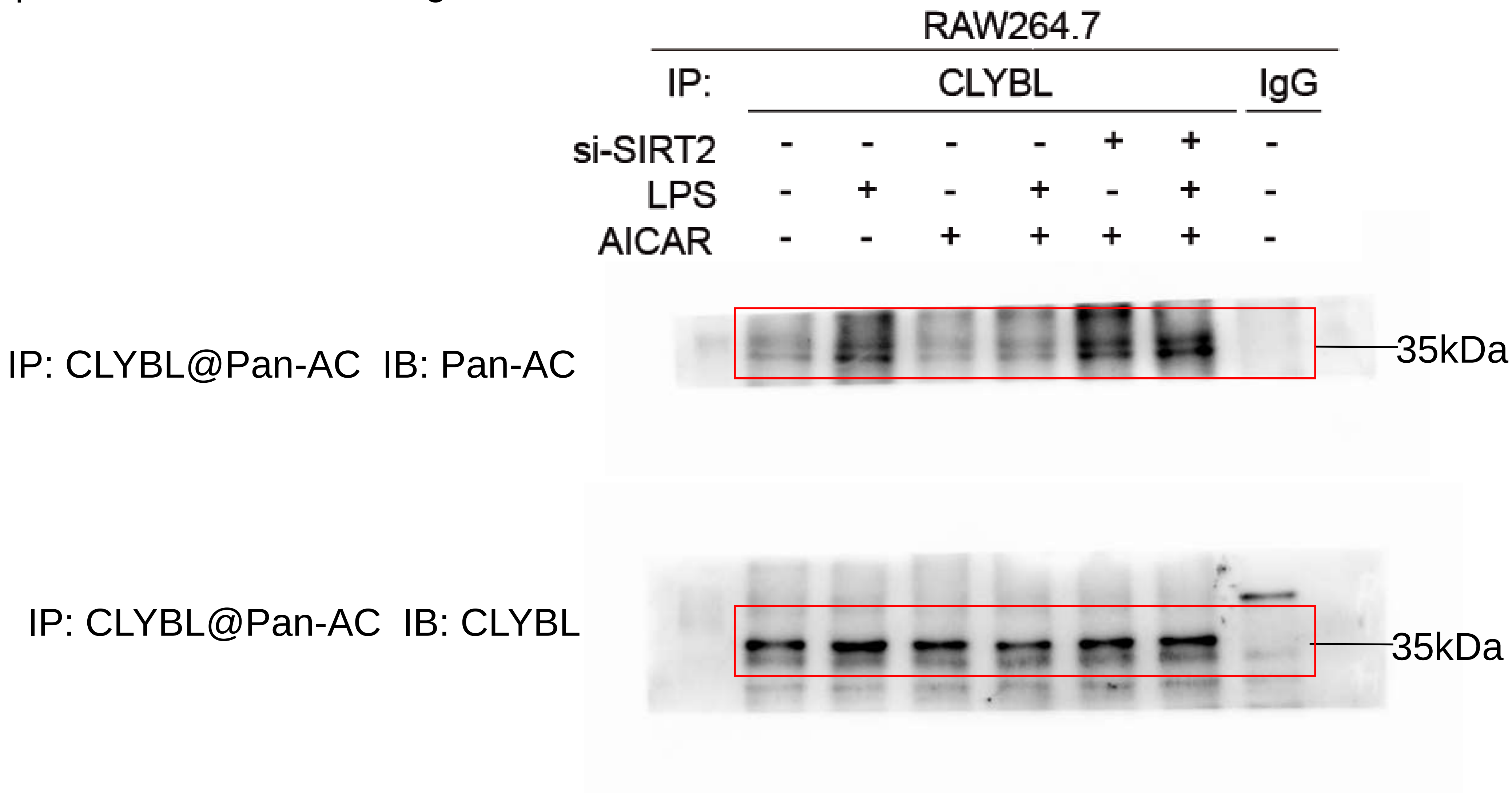

Uncropped Western blots for Figure 3 C

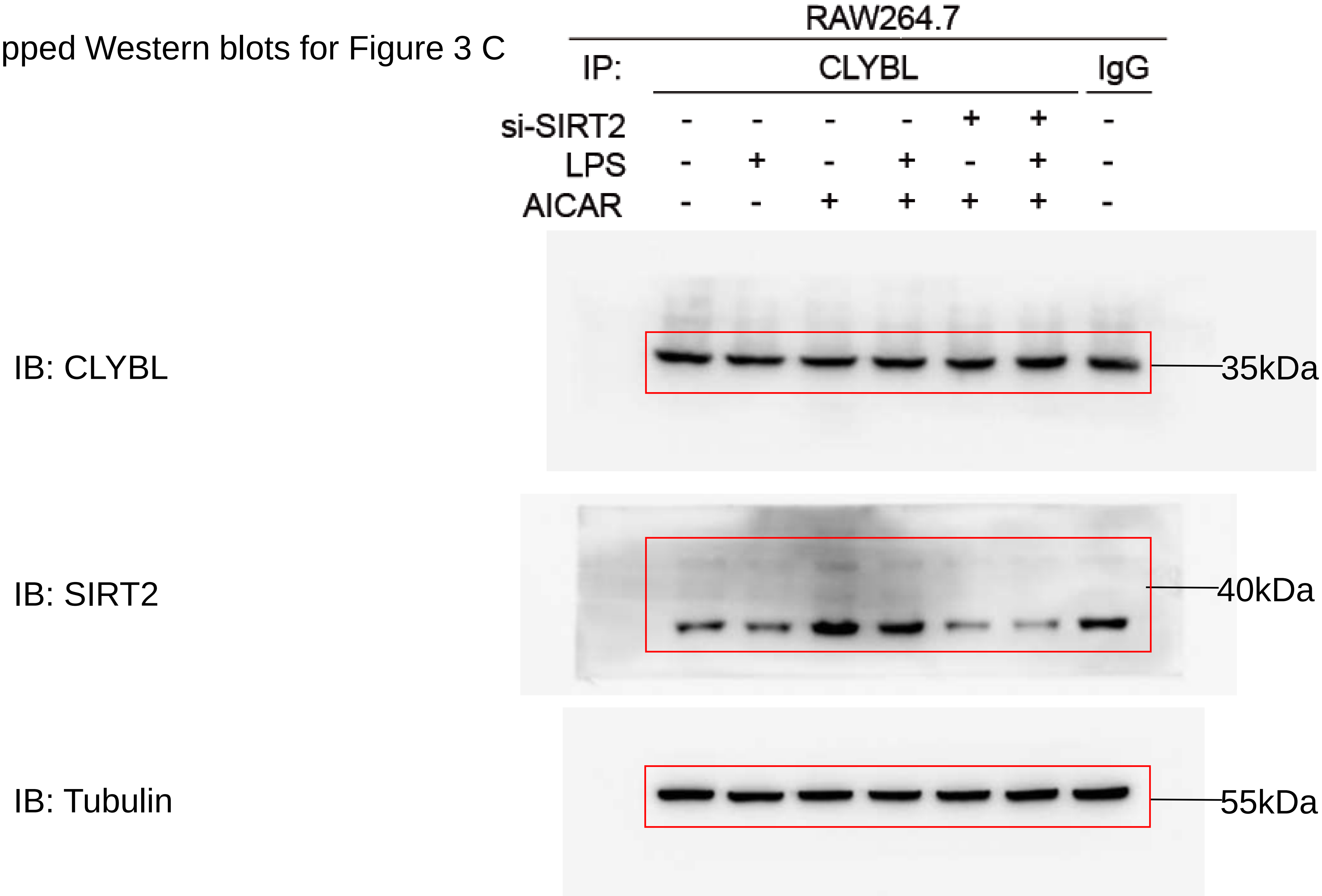

Uncropped Western blots for Figure 3 D

IB: P-AMPK

IB: AMPK

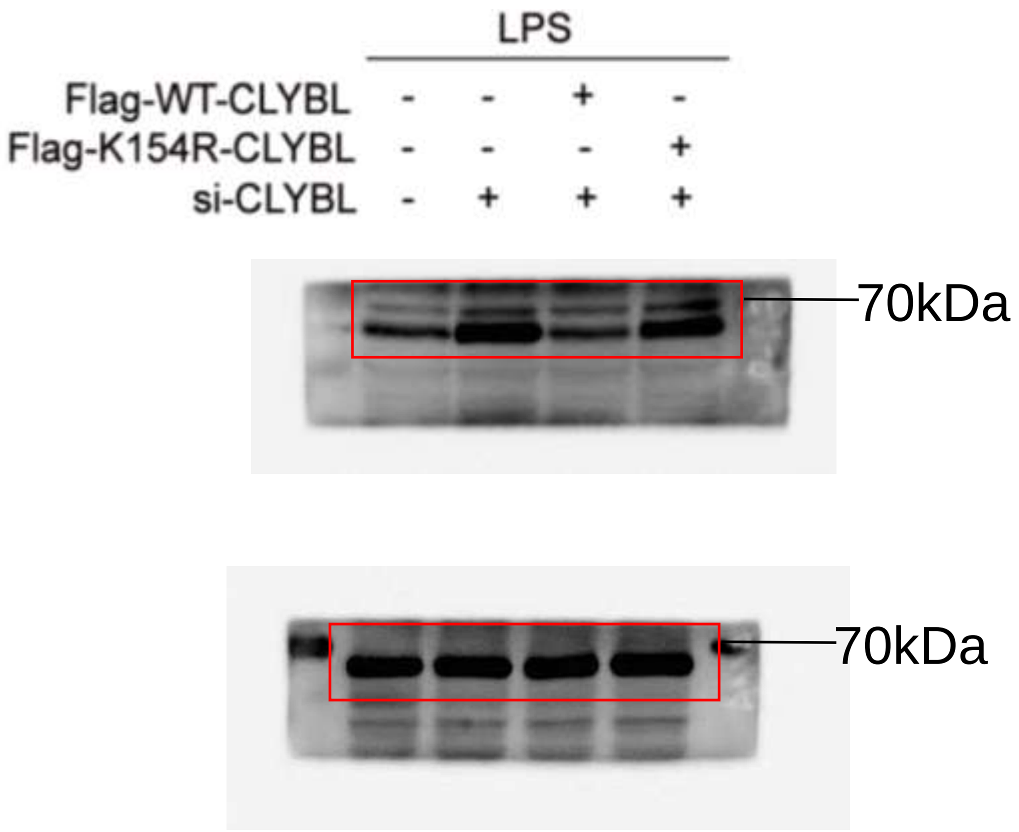

Uncropped Western blots for Figure 3 D

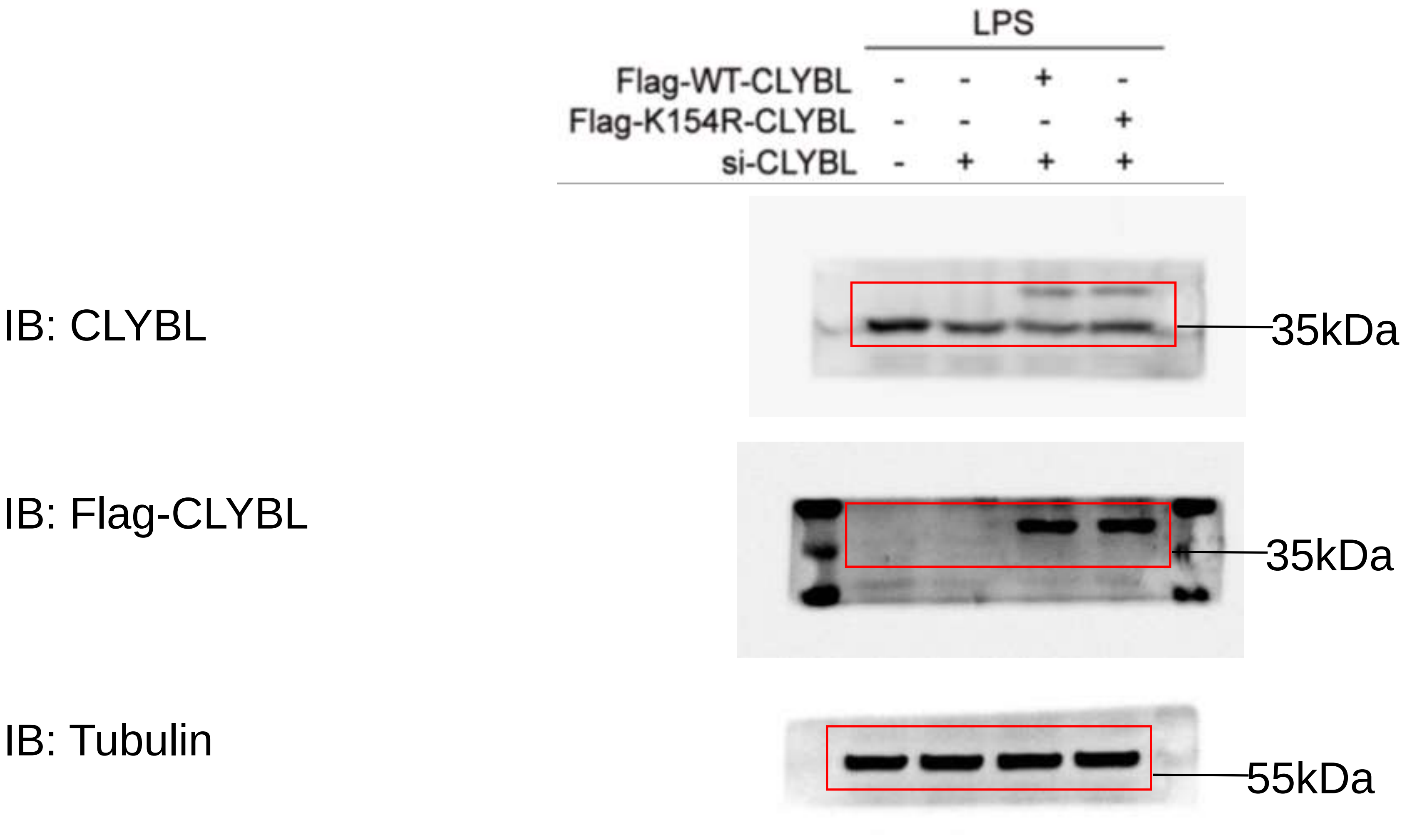

Uncropped Western blots for Figure 3 E

Flag-WT-CLYBL	-	-	+	-
Flag-K154R-CLYBL	-	-	-	+
si-CLYBL	-	+	+	+

IB: P-AMPK

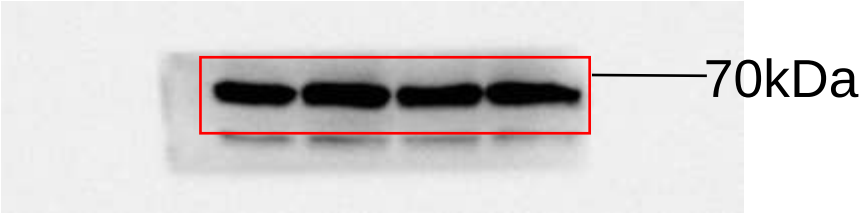

70kDa

IB: AMPK

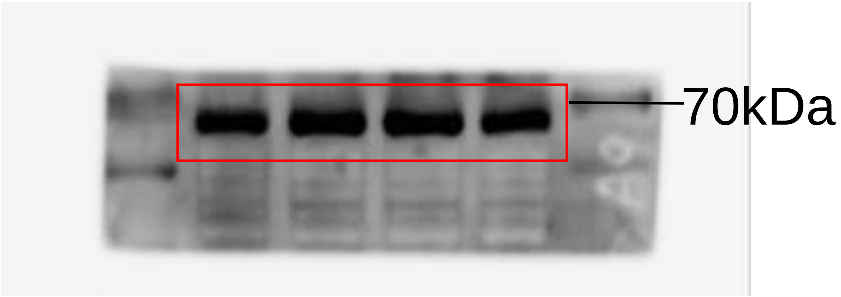

70kDa

Uncropped Western blots for Figure 3 E

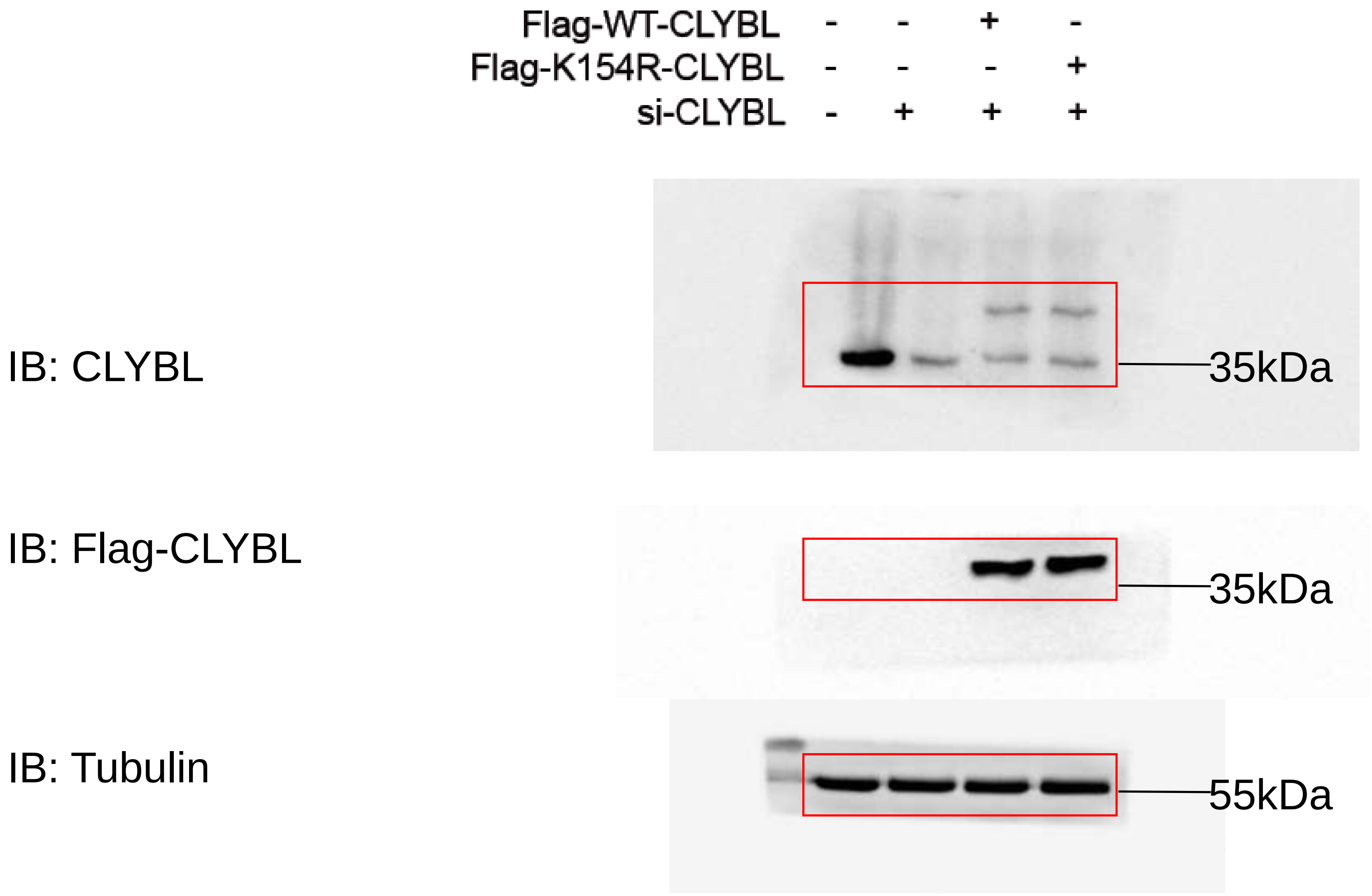

Uncropped Western blots for Figure 3 F

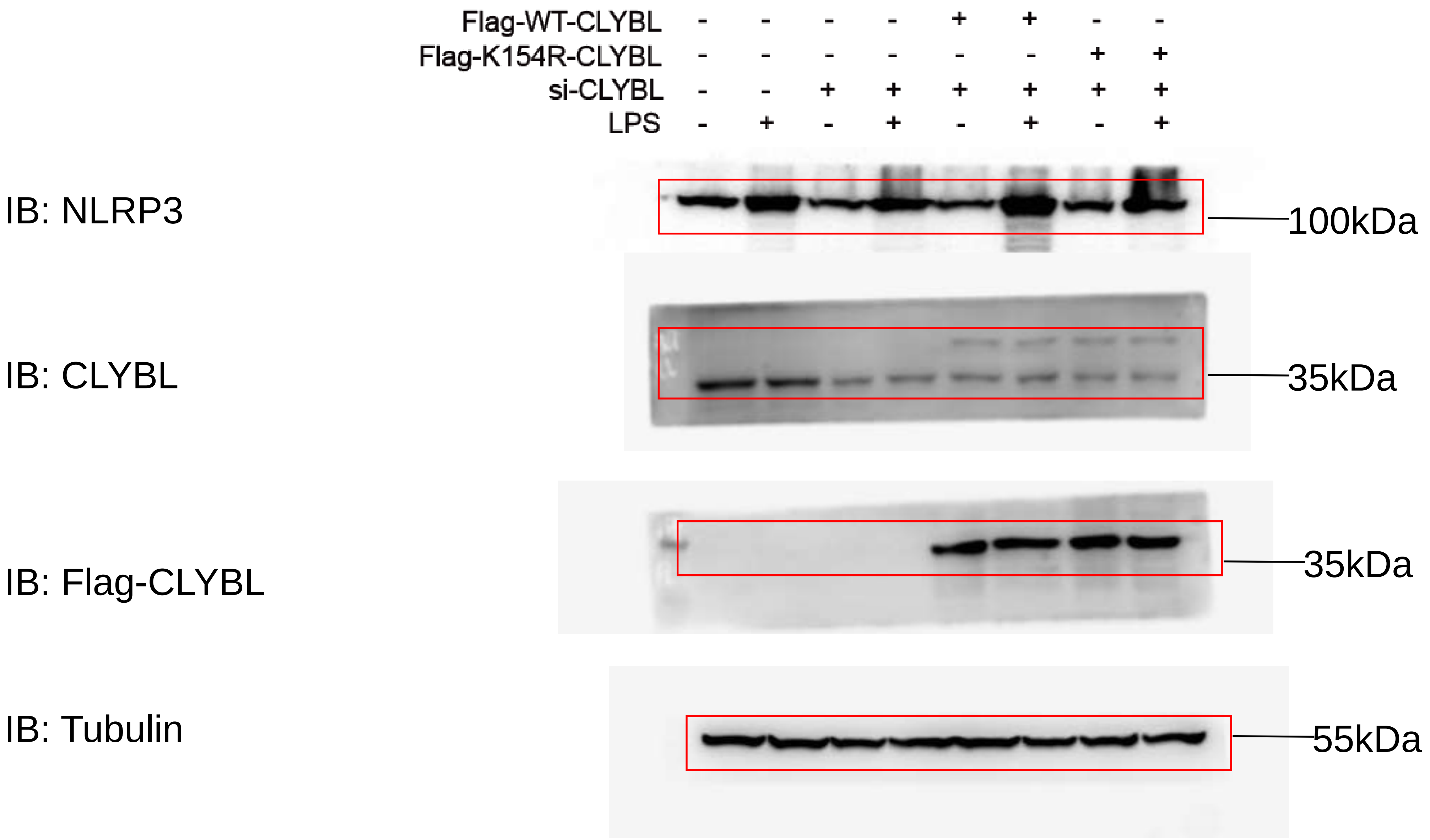

Uncropped Western blots for Figure 4

Uncropped Western blots for Figure 4 A

IP: CLYBL@Pan-AC IB: Pan-AC

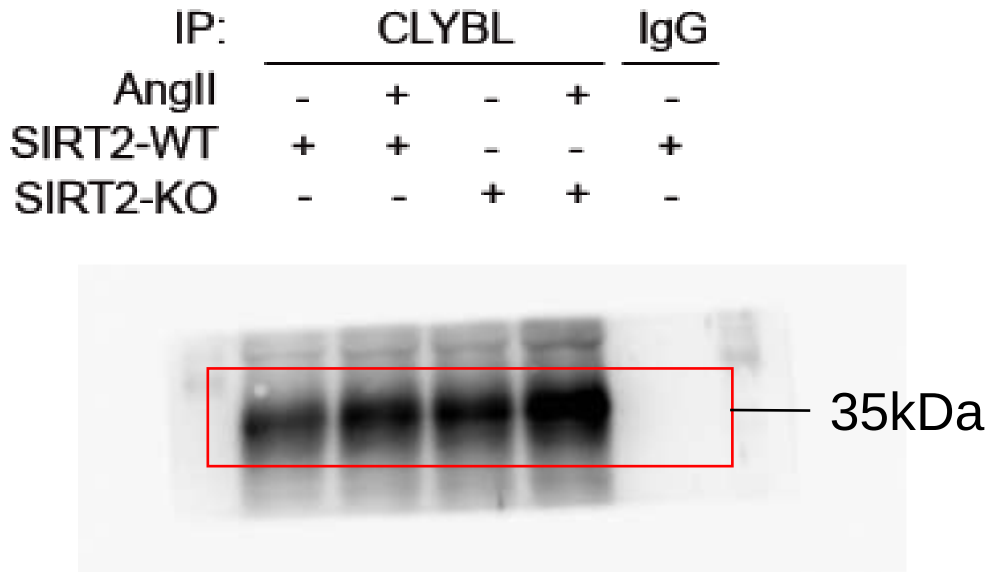

IP: CLYBL@Pan-AC IB: CLYBL

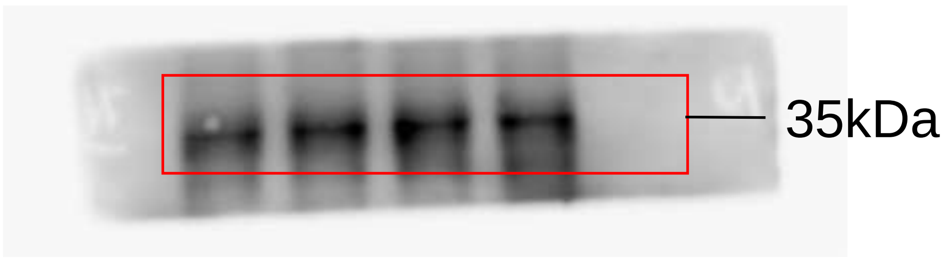

Uncropped Western blots for Figure 4 A

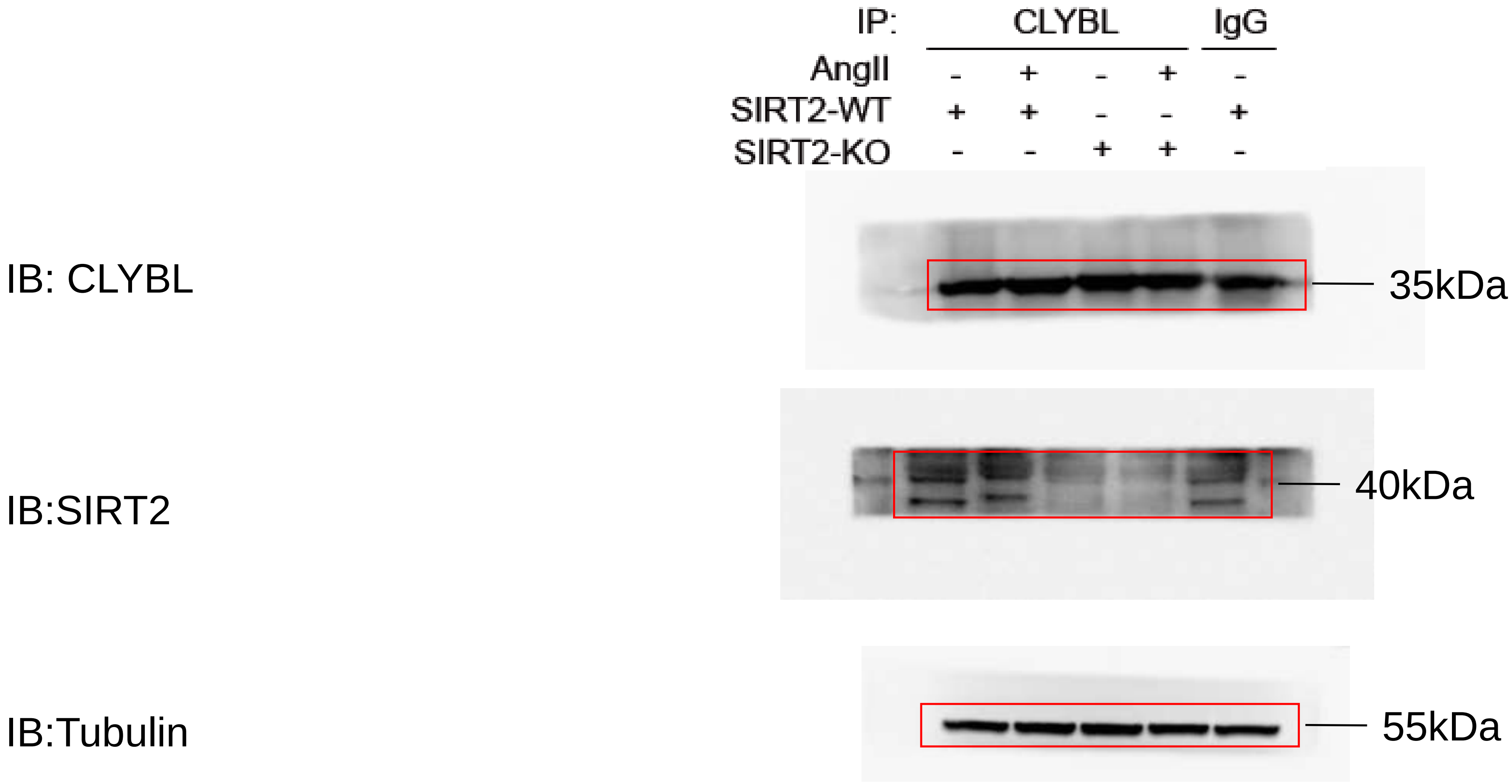

Uncropped Western blots for Figure 4 G

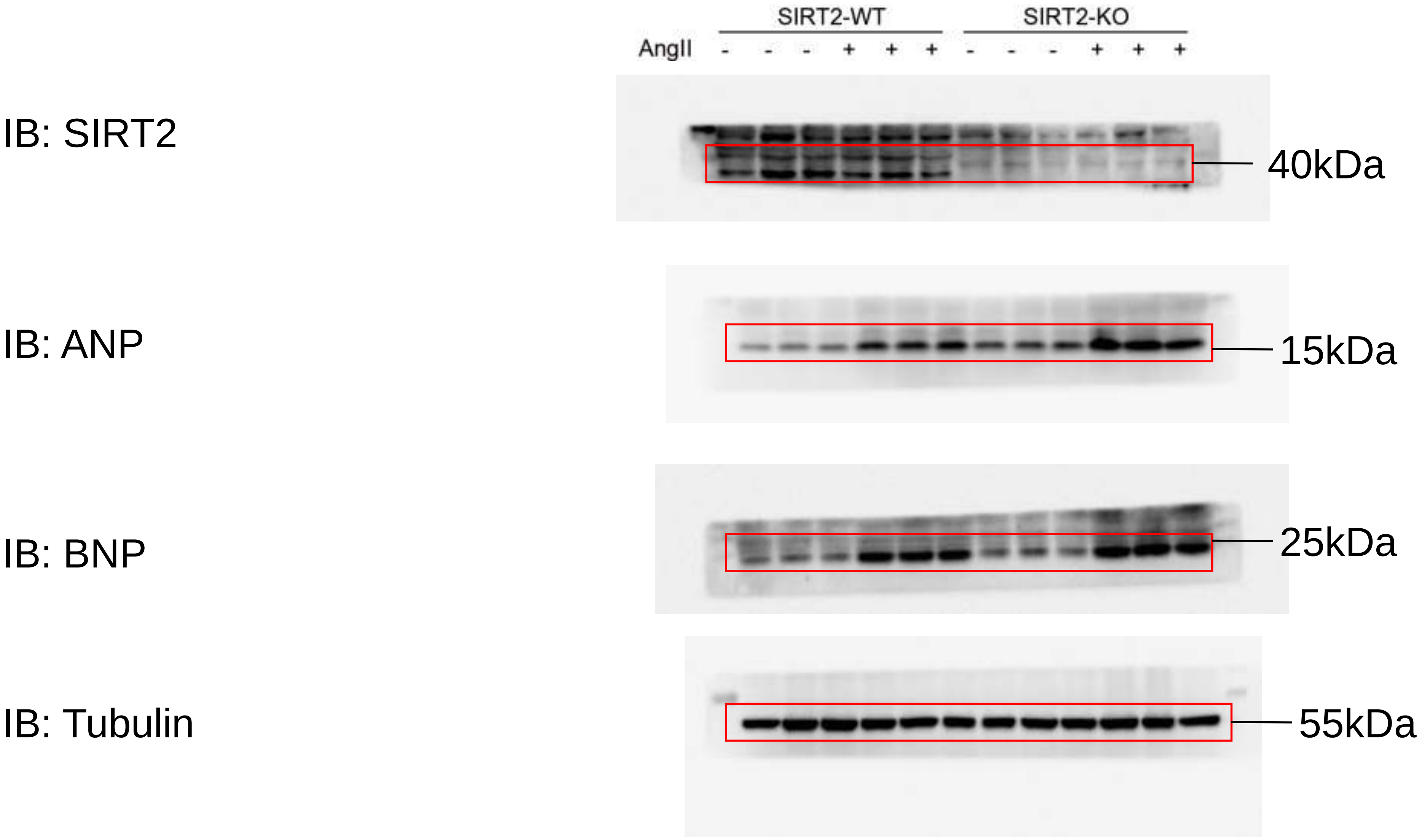

Uncropped Western blots for Figure 5

Uncropped Western blots for Figure 5 A

IP: CLYBL@Pan-AC IB: Pan-AC

IP: CLYBL@Pan-AC IB: CLYBL

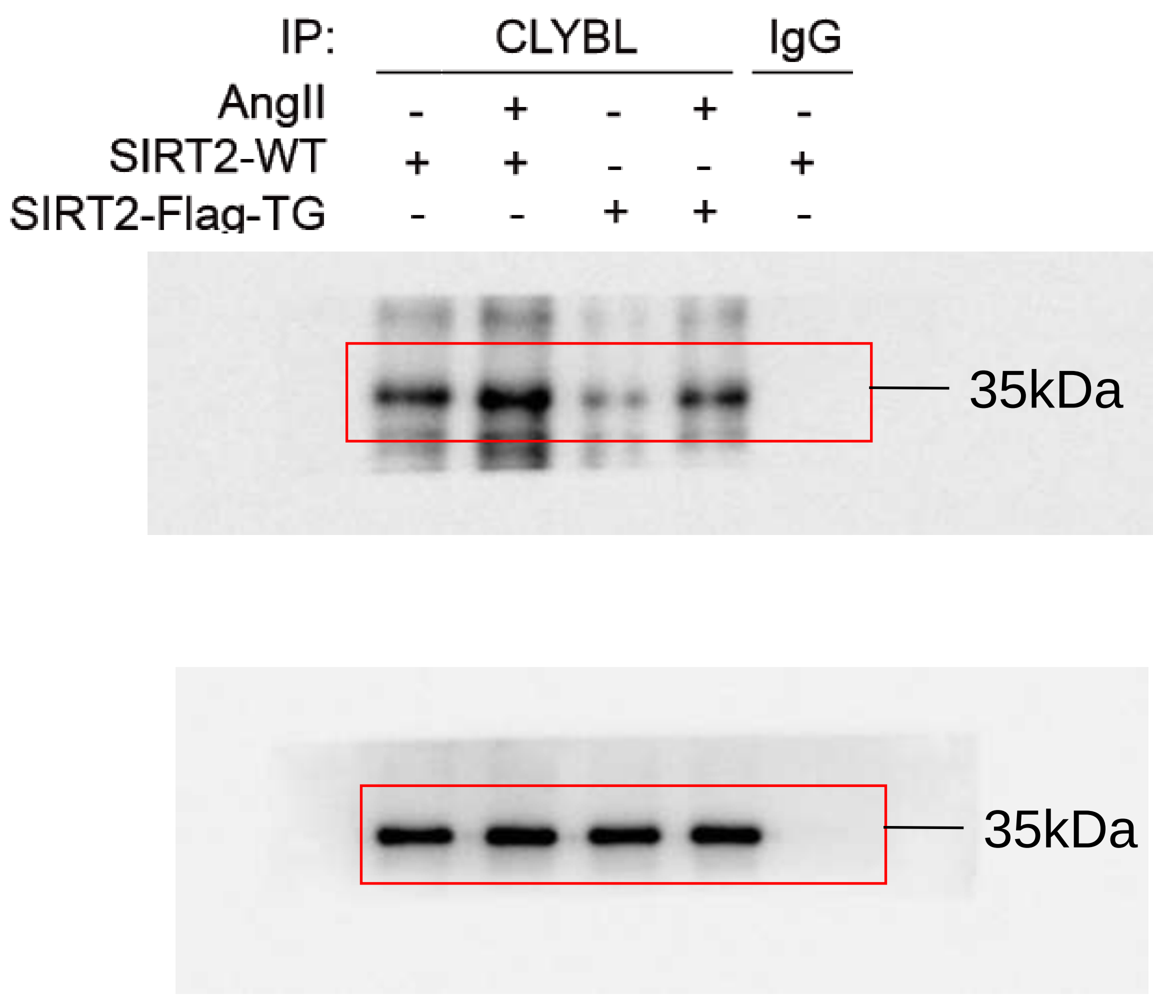

Uncropped Western blots for Figure 5 A

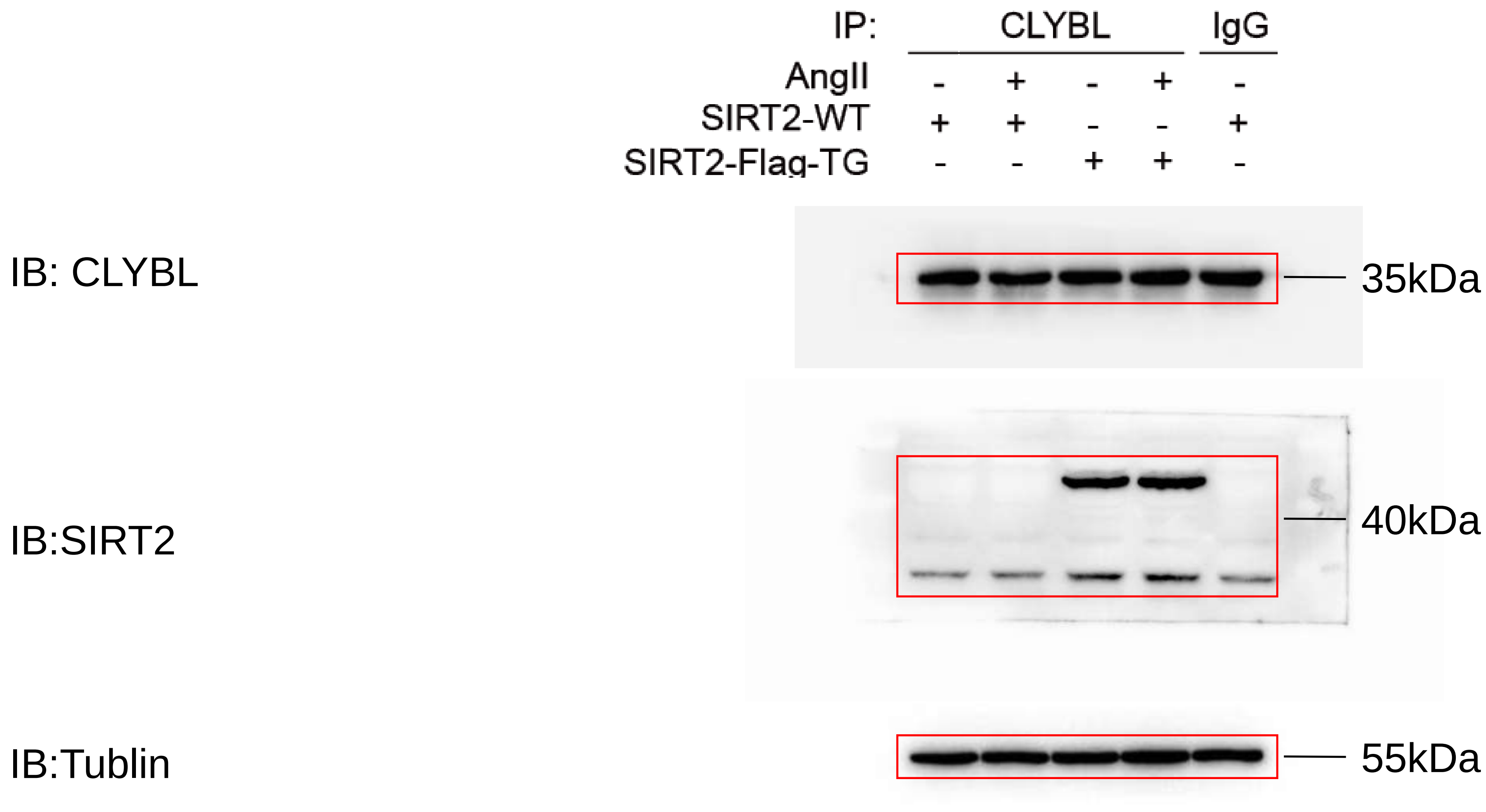

Uncropped Western blots for Figure 5 G

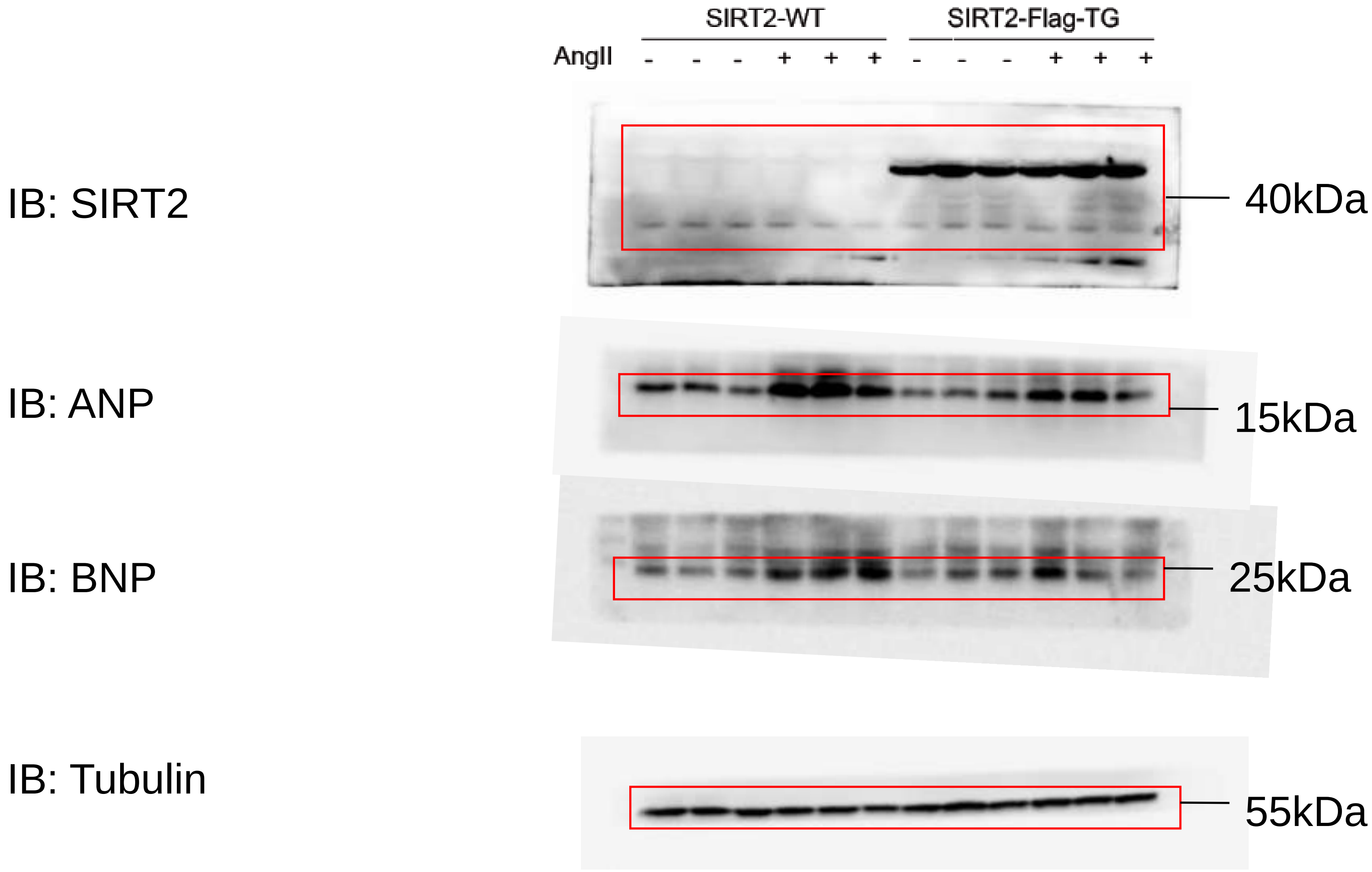

Uncropped Western blots for Supplemental Figure 2

Uncropped Western blots for S F 2 A

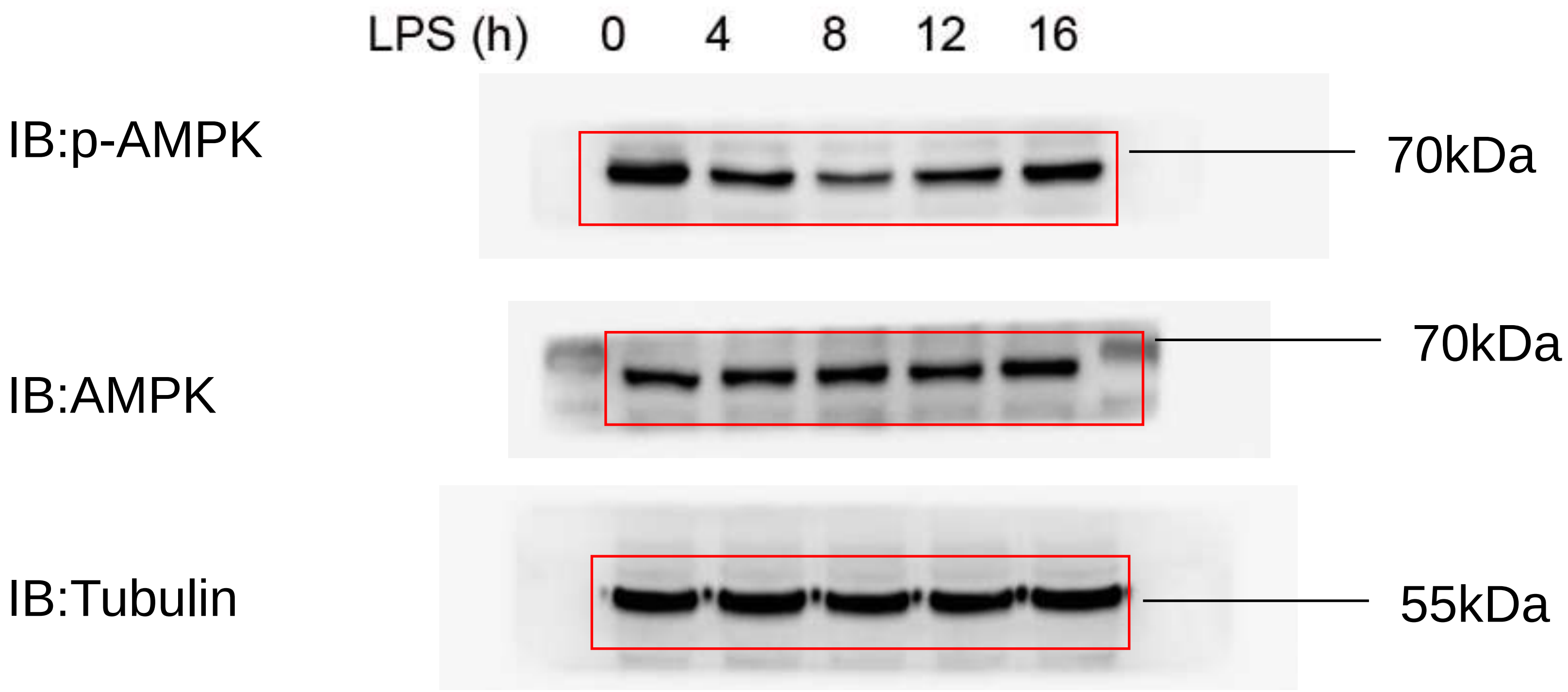

Uncropped Western blots for S F 2 B

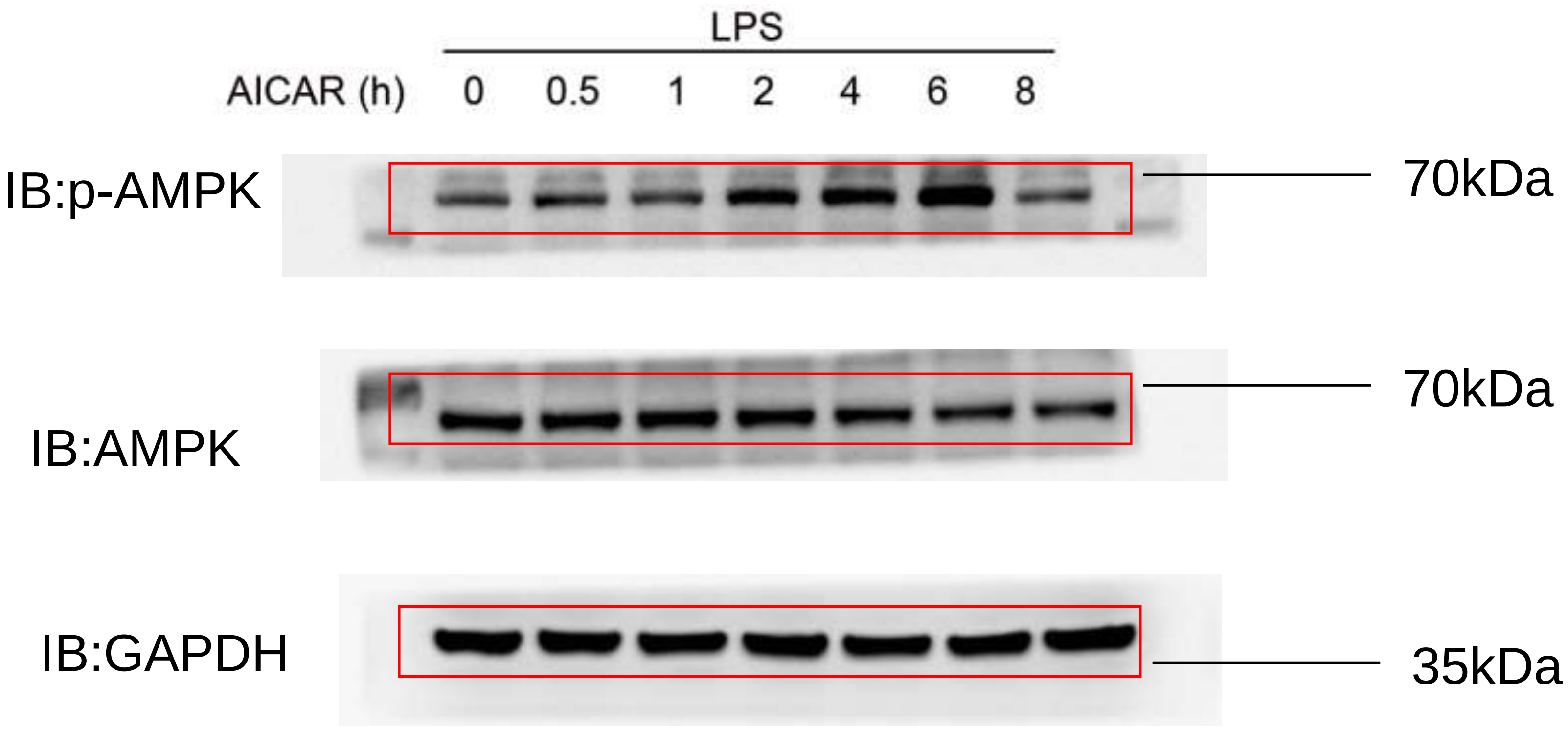

Uncropped Western blots for S F 2 C

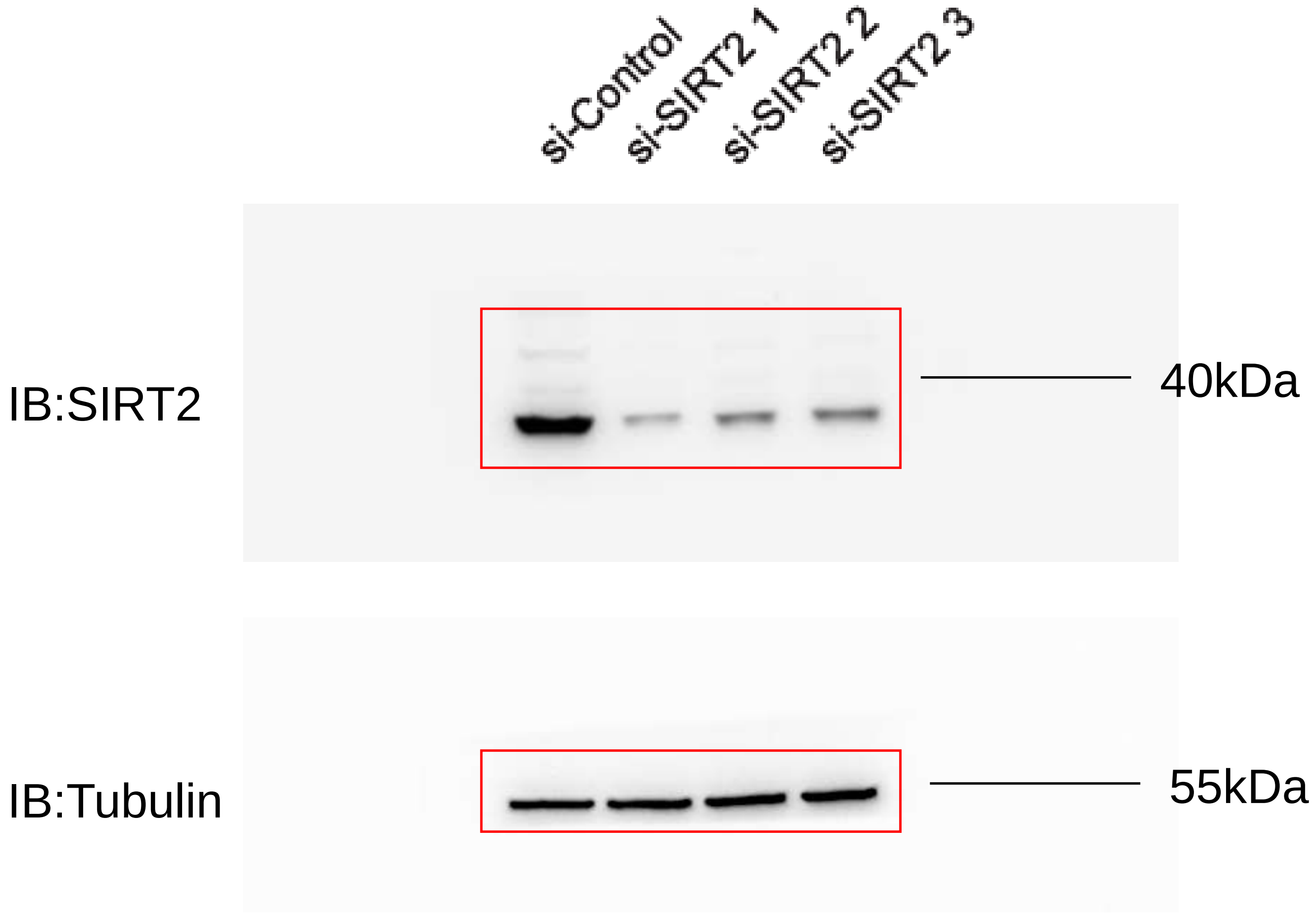

Supplement: Supplementary file 6 — Uncropped Western blots [file 41419_2025_7362_MOESM6_ESM.pdf]
